# Phenanthrenes from *Juncus articulatus* with Antibacterial and Biofilm Formation Inhibitory Activity

**DOI:** 10.1021/acs.jnatprod.4c00577

**Published:** 2024-08-09

**Authors:** Anita Barta, Agostina Salusso, Norbert Kúsz, Róbert Berkecz, Jan Schlauer, Dragica Purger, Judit Hohmann, Maria Cecilia Carpinella, Andrea Vasas

**Affiliations:** † Institute of Pharmacognosy, University of Szeged, Szeged 6720, Hungary; ‡ HUN-REN-USZ Biologically Active Natural Products Research Group, University of Szeged, Szeged 6720, Hungary; § Fine Chemical and Natural Products Laboratory, CIDIE CONICET-UCC, Universidad Católica de Córdoba, Córdoba X5016DHK, Argentina; ∥ Institute of Pharmaceutical Analysis, University of Szeged, Szeged 6720, Hungary; ⊥ The Center for Plant Molecular Biology, University of Tubingen, Tubingen 72076, Germany; # Department of Pharmacognosy, University of Pécs, Pécs 7624, Hungary

## Abstract

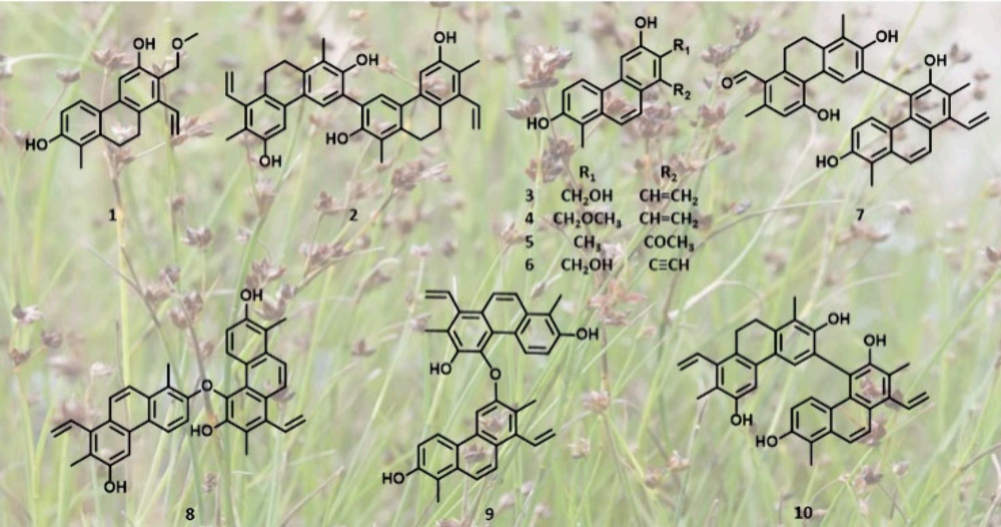

Continuing our search for bioactive compounds in species from the Juncaceae family, *Juncus articulatus* was investigated. Ten previously undescribed phenanthrenes—articulins A–J (**1**–**10**)—and ten known compounds—juncuenin B, dehydrojuncuenin B, juncatrin B, ensifolins E, F, H, I, K, juncuenin D, and luzulin A (**11**–**20**)—along with other compounds, have been isolated and identified. The isolated compounds were evaluated for antibacterial activity against *Escherichia coli*, *Pseudomonas aeruginosa*, methicillin-susceptible *Staphylococcus aureus* (MSSA), and methicillin-resistant *Staphylococcus aureus* (MRSA). Compounds **12** and **14** exhibited the most potent activity against planktonic and sessile MSSA and MRSA with minimum inhibitory concentration (MIC) values of 15.1 μM (**12** for both bacterial strains) and 15.3 μM (**14** for both bacterial strains). Compounds **15**, **17**, and **18** also exhibited activity against both strains, although to a lower extent, with MIC values ranging from 30.0 to 56.8 μM. The inhibition of biofilm formation of these compounds was observed at 15.1–114.3 μM. This study elucidates the phenanthrene composition of *J. articulatus* and the antibacterial effect of these compounds.

Since ancient times, bacterial infections caused high morbidities and mortality rates worldwide.^1,2^ Untreated infections or those where treatments fail due to ineffective antibiotic regimens or to unresponsive resistant bacteria may result in lethal conditions.^3−6^ Among these, sepsis is a major threat to healthcare systems with high economic costs.^7−10^ Another cause of therapeutic failures is the bacterial aggregates known as biofilms, whose structural matrices prevent antibacterial agents from reaching the cells.^11−13^ Consequently, the persistent bacterial communities become less sensitive to drugs and enhance the spread of antimicrobial tolerance.^14^ Under this scenario, considerable efforts are needed to develop effective therapeutic agents targeting planktonic, sessile-sensitive and resistant bacteria.

Plant-derived products are considered as promising sources for finding new bioactive compounds.^15,16^ Owing to biotic stress, plants produce biologically relevant secondary metabolites, many of which have antibacterial activity.^17^ The vast diversity of plants provide chemical entities with an array of chemical frameworks and pharmacophores that contribute to drug discovery pipelines.^18−22^ Among the natural compounds with outstanding pharmacological perspectives phenanthrenes have emerged as promising compounds with diverse biological properties, such as antiproliferative, anti-inflammatory, antioxidant, antialgal, and antimicrobial activities, including inhibition of MRSA growth.^23−25^

Phenanthrenoids are the main bioactive components of the Juncaceae species, categorized primarily into monophenanthrenes and diphenanthrenes.^25^ As a part of our ongoing research on isolating bioactive metabolites from the Juncaceae species, we investigated *Juncus articulatus* (*J. articulatus*), a rhizomatous perennial plant with terete culms that often roots at the nodes. It forms tufts or swards, often tinged with red, and ranges in height from 15 cm to about 50 cm. It grows in wet mud or sand in shallow along the margins of drains, still or slow-moving water, and wetlands.^26−28^ The plant may form dense mats that restrict water flow.^29^*J. articulatus* is native to Europe, Asia, North Africa, and Northern America and has been introduced in South Africa, Australia, and New Zealand.^30^ This species has not been previously investigated from phytochemical or pharmacological perspective.

This study focuses on isolating and determining the structures of phenanthrenes from *J. articulatus* and evaluating their effects on pathogenic bacteria. Ten previously undescribed phenanthrenes—articulins A–J (**1**–**10**)—and ten known phenanthrenes (**11**–**20**) were described from the plant. The antibacterial activity of the isolated compounds was evaluated against *Escherichia coli* (*E. coli*), *Pseudomonas aeruginosa* (*P. aeruginosa*), methicillin-susceptible *Staphylococcus aureus* (MSSA), and methicillin-resistant *Staphylococcus aureus* (MRSA) as well as on sessile cells of both *Staphylococcus aureus* (*S. aureus*) strains.

## RESULT AND DISCUSSION

The CHCl_3_ fraction of the MeOH extract prepared from the air-dried whole plant of *J. articulatus* was fractionated using a silica gel column and Sephadex LH-20 gel chromatography and purified by high-performance liquid chromatography (HPLC).

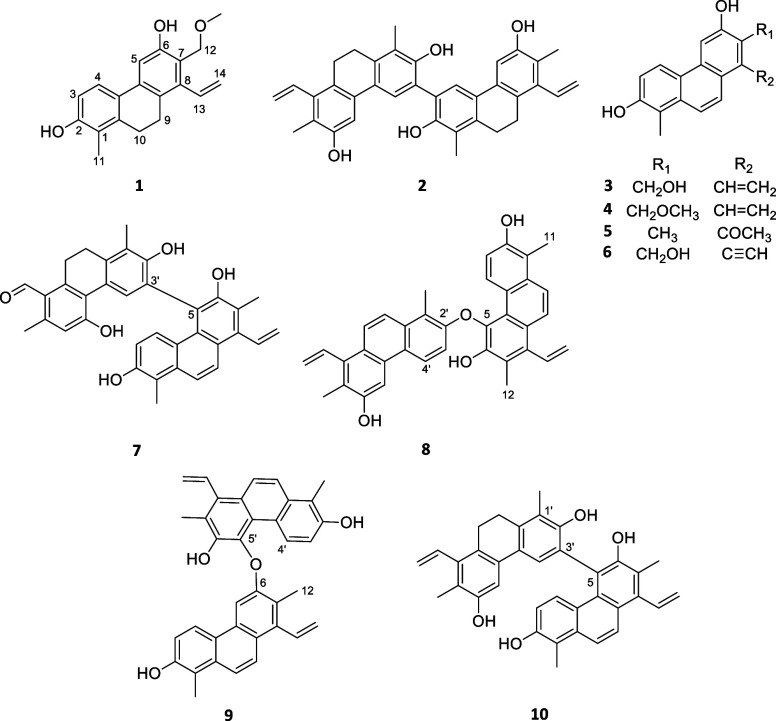



**Figure 1:**
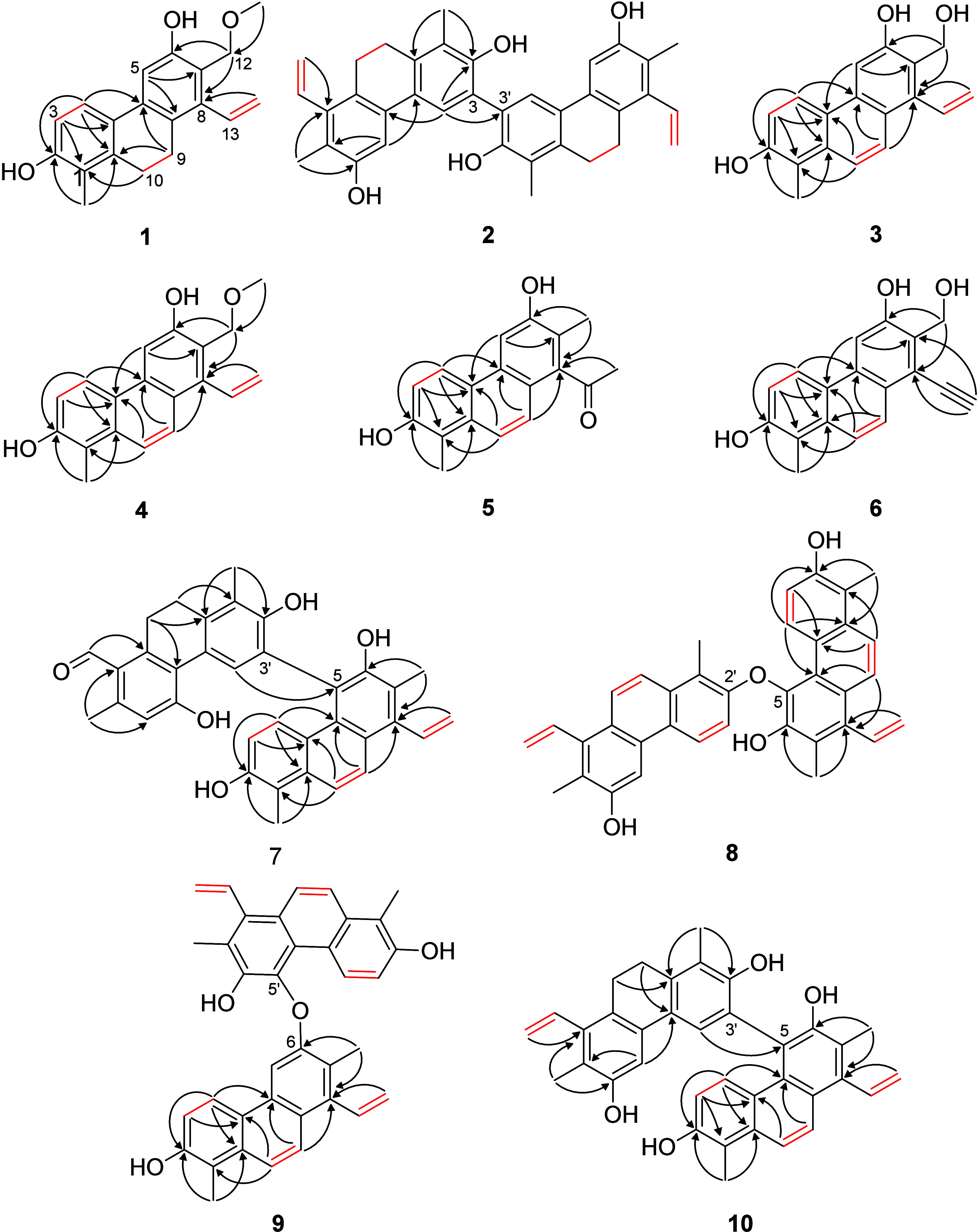
^1^H–^1^H COSY (**—**) and diagnostic HMBC (H → C) correlations of compounds **1**–**10**.

Compound **1** was obtained as an amorphous solid. Its molecular formula was determined as C_19_H_20_O_3_ by (−)-HRESIMS analysis (*m*/*z* 295.1342 [M–H]^−^, calcd for C_19_H_19_O_3_ 295.1340). The ^1^H NMR spectrum (Table 1) displayed signals of two *ortho*-coupled aromatic protons [δ_H_ 6.71 (1H, d, *J* = 8.4 Hz, H-3), 7.37 (1H, d, *J* = 8.4 Hz, H-4)], one aromatic proton as a singlet [δ_H_ 7.09 (1H, s, H-5)], a vinyl group [δ_H_ 6.86 (1H, dd, *J* = 17.8, 11.5  Hz, H-13), 5.28 (1H, d, *J* = 17.8  Hz, H-14a), and 5.58 (1H, d, *J* = 11.6 Hz, H-14b)], one methyl [δ_H_ 2.18 (3H, s, Me-11)], three aliphatic methylenes (including one oxygenated) [δ_H_ 2.70 (2H, m, H-9), 2.73 (2H, m, H-10) and 4.55 (2H, s, H-12)], and signals of protons belonging to one methoxy [δ_H_ 3.37 (3H, s, OCH_3_-12)] and two hydroxy groups. In the ^13^C NMR JMOD (*J*-modulated spin–echo experiment) spectrum, 19 carbon signals were detected, including two methyl groups, three aliphatic methylenes, an olefinic methylene, four aromatic or olefinic methines, and nine aromatic quaternary carbons (Table 1). The compound was indicated as a 9,10-dihydrophenanthrene derivative based on two methylene signals at δ_H_ 2.73 and 2.70 (2 × 2H) in the ^1^H NMR spectrum. In the ^1^H–^1^H COSY spectrum, correlations were observed between δ_H_ 6.71 and 7.37 d (H-3/H-4), and between δ_H_ 6.86 dd and 5.28 dd and 5.58 dd (H-13/H_2_-14) (Figure 1).

**Table 1:** ^1^H (500 MHz) and ^13^C (125 MHz) NMR Data of Compounds **1** and **3** in MeOH-*d*_4_ and Compound **2** in CDCl_3_ (δ in ppm)

	**1**		**2**		**3**	
position	δ_C_, type	δ_H_ (*J* in Hz)	δ_C_, type	δ_H_ (*J* in Hz)	δ_C_, type	δ_H_ (*J* in Hz)
1	122.3, C		122.7, C		118.4, C	
1a	138.9, C		138.6,^a^ C		133.9, C	
2	156.2, C		151.2, C		154.6, C	
3	113.9, CH	6.71 d (8.4)	120.6, C		117.0, CH	7.14 d (9.0)
4	123.4, CH	7.37 d (8.4)	123.8, CH	7.46 s	122.7, CH	8.30 d (9.0)
4a	127.7, C		126.8, C		124.6, C	
5a	138.0, C		133.5, C		133.6, C	
5	110.2, C	7.09 s	109.4, CH	7.06 s	106.3, CH	7.93 s
6	156.2, C		152.6, C		156.5, C	
7	120.6, C		120.8, C		125.4,^b^ C	
8	140.5, C		138.6,^a^ C		139.5, C	
8a	126.4, C		128.4, C		123.7, C	
9	26.5, CH_2_	2.70 m	25.8, CH_2_	2.86 m	125.4,^b^ CH	7.90 d (9.5)
10	26.6, CH_2_	2.73 m	25.8, CH_2_	2.82 m	120.5, CH	7.68 d (9.5)
11	11.5, CH_3_	2.18 s	12.0, CH_3_	2.33 s	11.0, CH_3_	2.51 s
12	68.0, CH_2_	4.55 s	12.9, CH_3_	2.23 s	58.8, CH_2_	4.91 s
13	135.9, CH	6.86 dd (17.8, 11.5)	135.2, CH	6.77 dd (17.9, 11.4)	135.3, CH	7.20 dd (17.9, 11.5)
14	120.7, CH_2_	5.28 d (17.8)	120.5, CH_2_	5.22 dd (17.9, 1.9)	122.2, CH_2_	5.47 dd (17.9, 2.1)
		5.58 d (11.6)		5.62 dd (11.4, 1.9)		5.79 dd (11.5, 2.1)
12-OCH_3_	57.9, CH_3_	3.37 s				

a Overlapping signals.

b Overlapping signals.

**Figure 2:**
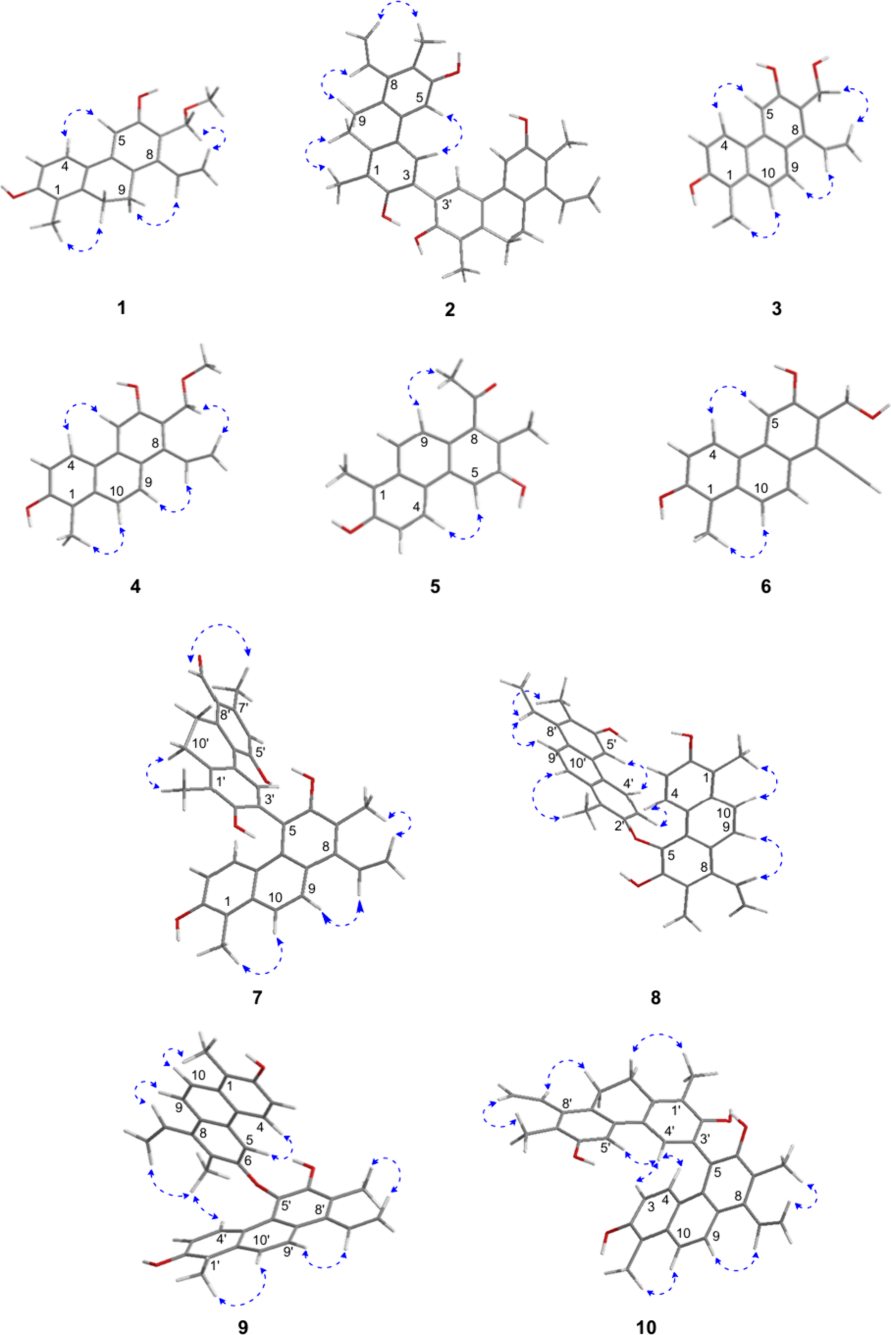
Key NOESY correlations (↔) for compounds **1**–**10**.

Heteronuclear long-range correlations (HMBC) of H-3 and H_2_-10 with C-4a, H-4 and H_2_-9 with C-5a, H-4, H_2_-9, and H_3_-11 with C-1a, and H_2_-10 with C-8a were used to establish a 9,10-dihydrophenanthrene skeleton for compound **1**. Furthermore, HMBC cross-peaks of H-3, H-4, and H_3_-11 with C-2 (δ_C_ 156.2), and H-5 and H_2_-12 with C-6 (δ_C_ 156.2), showed two hydroxy groups at C-2 and C-6. Based on the H-13/C-8a/, H-13/C-7/, and H_2_-14/C-8/ correlations, the vinyl moiety was placed at C-8, and its location at C-7 was confirmed by the strong cross-peak of the methoxy group (δ_H_ 3.37 s) and the methylene at δ_C_ 68.0 (Figure 1). In addition, the NOESY cross-peaks of H-4/H-5, H-9/H-13, H-10/H_3_-11, and H_2_-12/H_2_-14 further confirmed the structure of articulin A (**1**) (Figure 2). Compound **1** differs from juncuenin B (**11**), a phenanthrene previously isolated from several Juncaceae species,^25^ in having a methoxymethylene group in **1** instead of the methyl group (in **11**) at C-7. Finally, compound **1** was identified as 7-methoxymethylene-1-methyl-8-vinyl-9,10-dihydrophenanthren-2,6-diol and named articulin A.

Articulin B (**2**) was isolated as an amorphous solid. Analysis of ^1^H NMR, JMOD and HSQC data of **2** (Table 1) revealed two aromatic protons (δ_H_ 7.46 and 7.06 s; δ_C_ 123.8 and 109.4), two aromatic methyl groups (δ_H_ 2.33 and 2.23 s; δ_C_ 12.0 and 12.9), a vinyl system at δ_H_ 6.77 dd/δ_C_ 135.2 as well as 5.62 and 5.22 dd/δ_C_ 120.5 (H-13, H_2_-14), and two methylene groups (δ_H_ 2.86 and 2.82 m/δ_C_ 2 × 25.8). In addition to these data, the presence of methylene signals (H_2_-9 and H_2_-10) indicated that this compound was a 9,10-dihydrophenanthrene derivative. Since the HRESIMS indicated the molecular formula C_36_H_34_O_4_, (*m*/*z* 529.2378 [M–H]^−^, calcd for C_36_H_33_O_4_, 529.2379) a dimeric nature of compound **2** was assumed. Therefore, compound **2** was considered as a dihydrophenanthrenoid dimer comprising two juncuenin B (**11**) monomers. In compound **2**, the methine group at C-3 of juncuenin B was replaced by a quaternary carbon (δ_C_ 120.6). Given that compound **2** showed only 18 carbon signals instead of 36 in the JMOD spectrum, it was proposed that compound **2** is a symmetrical dimer of two juncuenin B units linked via C-3 and C-3′. This hypothesis was further confirmed by analysis of ^1^H–^1^H COSY and HMBC data (Figure 1). The HMBC long-range correlations observed from H-11 and H-4 to oxycarbon C-2 and from H-11 to C-1 and C-1a indicated the C-2 and C-1 positions of a hydroxyl and a methyl group at ring A, respectively. The correlation from H-4 to a quaternary carbon at δ_C_ 120.6 highlighted the linking site at C-3. The HMBC correlations from H-5 to C-7 and C-4a, H_3_-12 to C-6 and C-8, and H_2_-14 to C-8 indicated the hydroxyl, methyl and vinyl groups at ring C of the juncuenin B unit (Figure 1), respectively, thus establishing the structure of **2** as 1,1′,7,7′-tetramethyl-8,8′-divinyl-9,9′,10,10′-tetrahydro-[3,3′-biphenanthrene]-2,2′,6,6′-tetraol, named articulin B. Analysis of NOESY data showed cross-peaks of H-4/H-5, H_2_-9/H-13, H_2_-10/H_3_-11, and H_3_-12/H-13 that further confirmed the structure of articulin B (**2**) (Figure 2).

Based on the protonated molecular peak in the HRESIMS at *m*/*z* 281.1173 [M+H]^+^ (calcd for C_18_H_17_O_3_, 281.1172), the molecular formula of C_18_H_16_O_3_ was assigned to compound **3** (articulin C). The signals in the ^1^H NMR spectrum were similar to those of dehydrojuncuenin B (**12**), except for the replacement of a methyl group with an *O*-substituted methylene signal at δ_H_ 4.91 s.^31^ The methyl group at C-7 was oxidized into a hydroxymethyl side chain in compound **3** (Table 1). This assumption was substantiated by observing the HMBC correlations H_2_-12/C-6, H_2_-12/C-7, H_2_-12/C-8, H-5/C-7, and H_2_-14/C-8 (Figure 1). In addition, the NOESY cross-peaks of H-4/H-5, H-9/H-13, and H-10/H_3_-11 further confirmed the structure of articulin C (**3**) (Figure 2).

The HRESIMS data provided the molecular formula of C_19_H_18_O_3_ for articulin D (**4**) through a peak at *m*/*z* 293.1188 [M–H]^−^ (calcd for C_19_H_17_O_3_, 293.1183). The signals in the ^1^H NMR spectrum closely resembled those of articulin A (**1**) except for the replacement of its saturated H_2_-9/H_2_-10 structural part by two mutually coupled olefinic protons (δ_H_ 7.92 and 7.70, each 1H, *J* = 9.4 Hz) (Table 2). The double bond between C-9 and C-10 was supported by HMBC correlations between H-9 (δ_H_ 7.92) and C-1a, C-5a, and C-8 and between H-10 (δ_H_ 7.70) and C-1, C-4a, and C-8a (Figure 1). The H-4/H-5, H-9/H-13, and H-10/H_3_-11 NOE cross-peaks confirmed the structure of compound **4** (Figure 2).

**Table 2:** ^1^H (500 MHz) and ^13^C (125 MHz) NMR Data of Compounds **4**–**6** in MeOH-*d*_4_ (δ in ppm)

	**4**		**5**		**6**	
position	δ_C_, type	δ_H_ (*J* in Hz)	δ_C_, type	δ_H_ (*J* in Hz)	δ_C_, type	δ_H_ (*J* in Hz)
1	118.5, C		118.7, C		118.8, C	
1a	134.0,^a^ C		133.5, C		134.1, C	
2	154.7, C		154.6, C		154.9, C	
3	117.1, CH	7.15 d (9.0)	117.4, CH	7.16 d (9.0)	117.3, CH	7.17 d (9.0)
4	122.7, CH	8.30 d (9.0)	122.4, CH	8.28 d (9.0)	122.6, CH	8.30 d (9.0)
4a	124.6, C		124.5, C		124.4, C	
5a	134.1,^a^ C		132.5, C		133.5, C	
5	106.3, CH	7.93 s^b^	106.7, CH	7.94 s	108.6, CH	8.00 s
6	156.4, C		156.0, C		156.2, C	
7	122.8, C		120.5,^c^ C		130.8, C	
8	140.9, C		142.7, C		121.7, C	
8a	123.7, C		120.6,^c^ C		126.5, C	
9	125.4, CH	7.92 d (9.4)^b^	123.4, CH	7.38 d (9.4)	125.7, CH	8.19 d (9.4)
10	120.6, CH	7.70 d (9.4)	121.5, CH	7.74 d (9.4)	121.9, CH	7.80 d (9.4)
11	11.0, CH_3_	2.52 s	11.0, CH_3_	2.51 s	11.0, CH_3_	2.53 s (3H)
12	68.3, CH_2_	4.74 s	13.0, CH_3_	2.26 s	60.4, CH_2_	5.12 s (2H)
13	135.3, CH	7.20 dd (17.9, 11.5)	211.0, C		80.7, C	
14	122.1, CH_2_	5.49 dd (17.9, 2.1)	33.4, CH_3_	2.64 s	87.9, CH	4.12 s
		5.79 dd (11.5, 2.1)				
12-OCH_3_	58.1, CH_3_	3.43 s				

a Interchangeable signals.

b Overlapping signals.

c Interchangeable signals.

Articulin E (**5**) was obtained as a light-yellow amorphous solid. The HRESIMS suggested the molecular formula of C_18_H_16_O_3_, through a peak at *m*/*z* 281.1175 [M+H]^+^ (calcd for C_18_H_17_O_3_, 281.1172). The signals in the ^1^H NMR spectrum closely resembled those of juncatrin A, isolated previously from *J. atratus*, except for the replacement of its saturated H_2_-9/H_2_-10 structural part by two olefinic protons (δ_H_ 7.74 and 7.38, each 1H, *J* = 9.4 Hz) (Table 2).^32^ The double bond between C-9 and C-10 was supported by HMBC correlations between H-9 (δ_H_ 7.38) and C-1a, C-5a, and C-8, and H-10 (δ_H_ 7.74) and C-1, C-4a, and C-8a (Figure 1). The NOESY cross-peaks of H-4/H-5, and H-10/H_3_-11 further confirmed the structure of articulin E (**5**) (Figure 2).

The HRESIMS data provided the molecular formula of C_18_H_14_O_3_ for compound **6** (articulin F) through a peak at *m*/*z* 277.0870 [M–H]^−^ (calcd for C_18_H_13_O_3_, 277.0870). Comparing its 1D NMR data with those of ensifolin H, a 9,10-dihydrophenanthrene described from *J. ensifolius* by our research group,^33^ revealed two olefinic protons (δ_H_ 8.19 and 7.80, each 1H, *J* = 9.4 Hz) in the molecule instead of the saturated H_2_-9/H_2_-10 structural part (Table 2). This double bond was substantiated by HMBC correlations detected between H-9 (δ_H_ 8.19) and C-1a and C-5a, and between H-10 (δ_H_ 7.80) and C-1, C-4a, and C-8a (Figure 1). In addition, the NOESY cross-peaks of H-4/H-5, and H-10/H_3_-11 further confirmed the structure of articulin F (**6**) (Figure 2).

**Figure 3:**
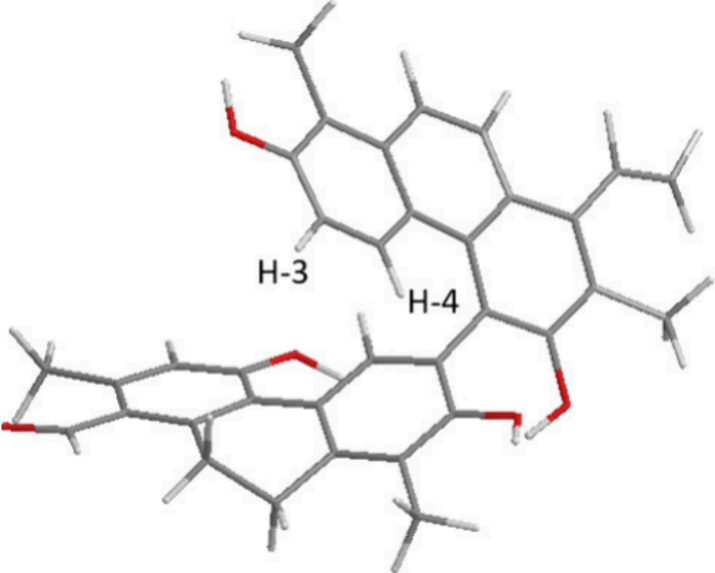
Calculated molecular structure of articulin G (**7**). It is shown that shielded H-3 and H-4 of the A monomer are situated within the shielding cone of the aromatic ring of the B monomer.

Articulin G (**7**), obtained as a light-yellow amorphous powder, is a phenanthrene heterodimer with the molecular formula of C_35_H_30_O_5_, determined by HRESIMS analysis (*m*/*z* 529.2021 [M–H]^−^, calcd for C_35_H_29_O_5_, 529.2021) and 35 carbon resonances detected in the ^13^C JMOD NMR spectrum (Table 3). One of the building blocks of compound **7** was identified as dehydrojuncuenin B (**12**) by analysis of 2D NMR data and comparison with assignments reported in the literature.^31^ In the 1D NMR spectra, the absence of resonances for another vinyl group along with a singlet signal at δ_H_ 10.51 and a carbonyl carbon at δ_C_ 193.9 indicated the biosynthetic conversion of the vinyl moiety of the second monomeric portion to an aldehyde moiety. Its position at C-8′ (δ_C_ 125.5) was assigned by observing HMBC correlations from H_3_-12′, H-6′, and H_2_-9′ to C-8′ (Figure 1). Careful analysis of the 2D NMR spectra concluded that this part of the molecule is a derivative of ensifolin E.^33^ Given the absence of H-5 in the dehydrojuncuenin B moiety and the correlation between a nonprotonated carbon at δ_C_ 126.9 (C-5) and the deshielded signal assigned to H-4′ (δ_H_ 7.90, 1H, s) of the other monomer, it was concluded that the monomers are connected through a C–C bond formed between C-5 of dehydrojuncuenin B and C-3′ of the other portion. The significantly shielded signal of H-3 (δ_H_ 6.53) and H-4 (δ_H_ 7.54) compared to that of the corresponding monomer dehydrojuncuenin B [H-3 (δ_H_ 7.12) and H-4 (δ_H_ 8.25)] further confirmed the proposed structure. This phenomenon was likely caused by the anisotropic effect of the aromatic ring of monomer B, indicating that H-3 and H-4 are located in the shielding cone of this ring (Figure 3).

**Table 3:** ^1^H (600 MHz) and ^13^C (150 MHz) NMR Data of Compounds **7** and **8** in MeOH-*d*_4_ (δ in ppm)

	**7**				**8**			
	unit A		unit B		unit A		unit B	
position	δ_C_, type	δ_H_ (*J* in Hz)	δ_C_, type	δ_H_ (*J* in Hz)	δ_C_, Type	δ_H_ (*J* in Hz)	δ_C_, type	δ_H_ (*J* in Hz)
1	117.5, C		123.5, C		117.9, C		121.3, C	
1a	134.9, C		140.3, C		134.4, C		133.4, C	
2	153.4, C		152.8, C		154.0, C		154.0, C	
3	115.4, CH	6.53 d (9.4)	126.9, C		116.2, CH	6.82 d (9.4)	113.0, CH	6.42 d (9.4)
4	127.1, CH	7.54 d (9.4)	130.3, CH	7.90 s	127.1, CH	8.98 d (9.4)	125.6, CH	8.05 d (9.4)
4a	125.6, C		118.6, C		123.6, C		126.4, C	
5a	131.1, C		122.7, C		125.2, C		131.8, C	
5	126.9, CH		159.9, C		n.d.^a^		105.6, CH	7.74 s
6	153.4, C		118.5, CH	6.59 s	148.9, C		156.0, C	
7	123.5, C		143.2, C		124.9, C		124.2, C	
8	138.9, C		125.5, C		135.7, C		138.6, C	
8a	123.5, C		146.0, C		125.3, C		124.1, C	
9	125.8, CH	8.04 d (9.5)	26.5, CH	3.44 m	122.5, CH	8.06 d (9.6)	126.0, CH	8.02 d (9.6)
				3.11 m				
10	120.9, CH	7.73 d (9.5)	26.3, CH	2.96 m	121.4, CH	7.76 d (9.6)	120.0, CH	7.87 d (9.6)
				2.75 m				
11	11.2, CH_3_	2.47 s	12.4, CH_3_	2.35 s	11.2, CH_3_	2.47 s	11.9, CH_3_	3.03 s
12	14.5, CH_3_	2.45 s	20.8, CH_3_	2.53 s	14.2, CH_3_	2.46 s	13.6, CH_3_	2.32 s
13	136.9, CH	7.19 dd (17.9, 11.5)	193.9, C	10.51 s	136.5, CH	7.19 dd (17.9, 11.4)	136.3, CH	7.10 dd (17.9, 11.4)
14	121.8, CH_2_	5.85 dd (11.4, 2.1)			122.1, CH_2_	5.88 dd (11.3, 2.0)	121.6, CH_2_	5.81 dd (11.4, 2.0)
		5.41 dd (17.9, 2.1)				5.44 dd (17.8, 2.0)		5.37 dd (17.9, 2.0)

a n.d. not detected.

**Figure 4:**
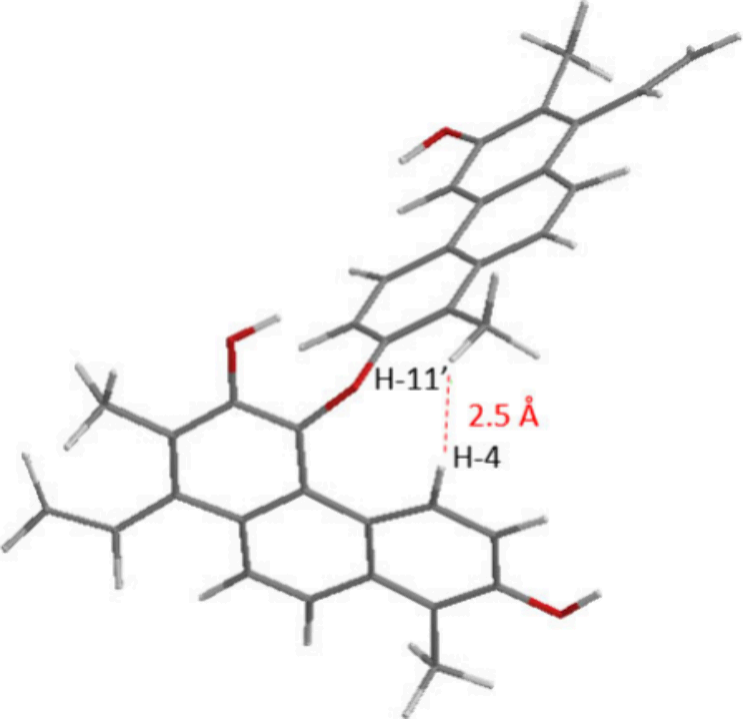
Energy-minimized 3D structure of articulin H (**8**) and atom distance between H-4 and H-11′.

Articulin H (**8**) was obtained as an amorphous solid. Its molecular formula C_36_H_30_O_4_ was established by HRMS analysis (*m*/*z* 525.2071 [M–H]^−^, calcd for C_36_H_29_O_4_, 525.2071). Its JMOD spectrum displayed 36 signals, suggesting that compound **8** is a phenanthrene dimer (Table 3). Analysis of the ^1^H NMR spectrum revealed that articulin H (**8**) comprises two dehydrojuncuenin B (**12**) monomers (Table 3). Unlike articulin B (**2**), compound **8** is not symmetrical as its 1D NMR data provided two sets of proton and carbon resonances assigned to the two subunits. The strong NOE from H_3_-11′ to H-4 indicated that the monomers are linked through an ether bond formed between the OH-2′ group of one dehydrojuncuenin B monomer and the C-5 carbon of the other monomer (Figures 2 and 4).

Compound **9** (articulin I) presented the molecular formula C_36_H_30_O_4_ compatible with its molecular peak at *m*/*z* 525.2074 [M–H]^−^ (calcd for C_36_H_29_O_4_, 525.2071). The 36 carbon signals in the ^13^C NMR JMOD spectrum suggested that compound **9** is a phenanthrene dimer. Analysis of its ^1^H NMR spectrum and ^1^H–^1^H COSY correlations, and comparison of spectral data with literature values showed two dehydrojuncuenin B (**12**) units in **9** (Table 4). Given the absence of proton signal at C-5′ in the ^1^H NMR spectrum, and the strong NOE correlation between H_3_-12 and H-4′, the monomers must be attached through an ether bond between C-6 and C-5′ (Figure 2).

**Table 4:** ^1^H (600 MHz) and ^13^C (150 MHz) NMR Data of Compounds **9** and **10** in MeOH-*d*_4_ (δ in ppm)

	**9**				**10**			
	unit A		unit B		unit A		unit B	
position	δ_C_, type	δ_H_ (*J* in Hz)	δ_C_, type	δ_H_ (*J* in Hz)	δ_C_, type	δ_H_ (*J* in Hz)	δ_C_, type	δ_H_ (*J* in Hz)
1	118.2, C		118.0, C		117.5, C		124.6, C	
1a	133.3, C		134.4, C		135.0, C		139.1, C	
2	154.2, C		154.0, C		153.4, C		153.3, C	
3	117.0, CH	6.83 d (9.3)	116.3, CH	6.86 d (9.6)	115.3, CH	6.53 d (9.4)	n.d.	
4	121.9, CH	7.36 d (9.3)	126.9, CH	8.97 d (9.6)	127.4, CH	7.57 d (9.4)	125.6, CH	7.19 s
4a	124.6, C		123.6, C		125.6, C		126.2, C	
5a	131.5, C		125.0, C		131.2, C		109.8, C	
5	103.4, CH	7.22 s	137.8, C		118.9, C		134.5, CH	6.78 s
6	155.5, C		148.7, C		153.5, C		154.8, C	
7	124.9, C		125.2, C		123.5, C		121.9, C	
8	139.1, C		135.8, C		138.8, C		139.2, C	
8a	125.1, C		125.5, C		125.2, C		130.4, C	
9	125.1, CH	7.96 d (9.8)	125.6, CH	8.12 d (9.8)	125.8, CH	8.04 d (9.5)	27.0, CH_2_	2.89 m
10	121.3, CH	7.67 d (9.8)	121. 5, CH	7.77 d (9.8)	120.9, CH	7.74 d (9.5)	26.9, CH_2_	2.85 m
11	10.9, CH_3_	2.41 s	11.2, CH_3_	2.47 s	11.2, CH_3_	2.48 s	12.4, CH_3_	2.36 s
12	14.4, CH_3_	2.85 s	14.3, CH_3_	2.49 s	14.5, CH_3_	2.44 s	13.1, CH_3_	2.11 s (3H)
13	136.5,^a^ CH	7.25 dd^c^^,^^d^ (18.3, 11.6)	136.2,^a^ CH	7.22 dd^c^^,^^d^ (18.3, 11.6)	136.9, CH	7.20 dd (17.4, 11.4)	136.8, CH	6.79 dd (18.0, 11.4)
14	122.2,^b^ CH_2_	5.48 dd^e^^,^^f^(18.3, 2.0)	122.1,^b^ CH_2_	5.49 dd^e^^,^^f^ (18.3, 2.0)	121.8, CH_2_	5.40 dd (17.4, 2.2)	120.3, CH_2_	5.60 dd (11.4, 2.2)
		5.91 dd^g^^,^^h^ (11.6, 2.0)		5.91 dd^g^^,^^h^ (11.6, 2.0)		5.86 dd (11.4, 2.2)		5.17 dd (18.0, 2.2)

a Interchangeable signals.

b Interchangeable signals.

c Interchangeable signals.

d Overlapping signals, n.d. not detected.

e Interchangeable signals.

f Overlapping signals, n.d. not detected.

g Interchangeable signals.

h Overlapping signals, n.d. not detected.

The molecular formula of compound **10** (articulin J) is C_36_H_32_O_4_ based on HRESIMS analysis (*m*/*z* 527.2224 [M–H]^−^, calcd for C_36_H_31_O_4_, 527.2222). Analysis of ^1^H NMR, ^13^C NMR JMOD, and HSQC data of this compound revealed a heterodimeric phenanthrenoid structure comprising a dihydrophenanthrene and a phenanthrene unit (Table 4). The ^1^H and ^13^C resonances of the phenanthrene part closely resembled those of unit A of articulin G (**7**), and the dihydrophenanthrene part could be elucidated as a 3-substituted juncuenin B (**11**). The C-5–C-3′ connection of the two monomers was proved by observing HMBC correlations of H-4′ (δ_H_ 7.19 s) and H_3_-12 (δ_H_ 2.44 s) with C-5 (δ_C_ 118.9) (Figure 1) and NOESY correlations of H-4′ (δ_H_ 7.19 s) with H-3 (δ_H_ 6.53 d, *J* = 9.4 Hz) and H-4 (δ_H_ 7.57 d, *J* = 9.4 Hz) (Figure 2). The anisotropic effect of the aromatic ring of monomer B, which caused shielding of H-3 and H-4 signals, can be observed similarly in articulins I (**9**) and J (**10**), as mentioned for **7**.

In addition to the ten new articulins A–J (**1**–**10**), ten known phenanthrenes were also isolated, including juncuenin B (**11**),^31^ dehydrojuncuenin B (**12**),^31^ juncatrin B (**13**),^32^ juncuenin D (**15**),^31^ luzulin A (**16**),^34^ ensifolins E (**18**), F (**19**), H (**17**), I (**14**),^33^ ensifolin K (**20**),^33^ as well as apigenin and luteolin,^35^ 1,3-*O*-diferuloylglycerol,^36^ (+)-*S*-dehydrovomifoliol,^37^ vanillin,^38^ and *p*-hydroxybenzaldehyde.^38^

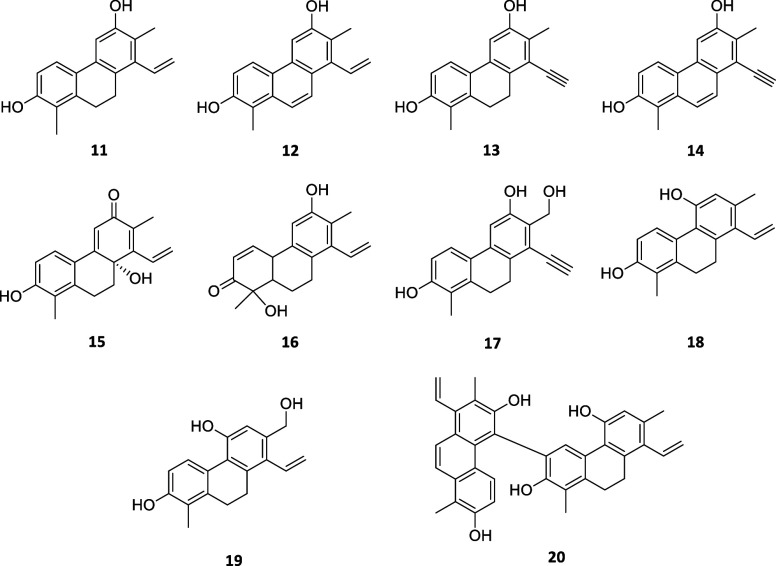



Given that juncuenin B (**11**) and dehydrojucuenin B (**12**) were isolated in high amounts, they are considered the main phenanthrenes of *J. articulatus*, and the isolated phenanthrenes are mainly derivatives of these two compounds. Compounds **1** and **2**, juncatrin B (**13**), juncuenin D (**15**), luzulin A (**16**), and ensifolin H (**17**) derive from juncuenin B (**11**), while compounds **3**–**10** and ensifolin I (**14**) originate from dehydrojuncuenin B (**12**). In articulins H (**8**) and I (**9**), two dehydrojuncuenin B (**12**) units are connected through an ether bridge, while articulin J (**10**) is the dimer originating from a juncuenin B (**11**) and a dehydrojuncuenin B (**12**) unit.

Considering that the MeOH extract of *J. articulatus* was previously reported to inhibit the growth of MRSA,^39^ the isolated compounds were tested for their inhibitory effects on the growth of planktonic *E. coli*, *P. aeruginosa*, MSSA, and MRSA as well as on sessile cells of both *S. aureus* strains. Compounds **2**, **3**, **6**, **9**, **12**–**15**, and **17**–**20** inhibited the growth of MSSA and MRSA with minimum inhibitory concentration (MIC) values below 64 μg/mL. Compounds **12**, **14**, **15**, **17**, and **18** exerted activity with MIC values ranging from 4 to 16 μg/mL (Table 5). Compounds **12** and **14** were the most potent, exhibiting MIC values of 15.1 and 15.3 μM, respectively. The activity of these phenanthrenes partly explain the inhibition of MRSA growth observed for *J. articulatus*.^39^ None of the target metabolites inhibited Gram-negative bacteria. Previous studies demonstrated that dehydrojuncuenin B (**12**) inhibited MRSA growth with an MIC value of 25 μg/mL, while juncuenin D (**15**) did the same at 12.5 μg/mL,^40^ with the latter being more active than compound **12**, contrary to the observations in this work. To our knowledge, this is the first report of the antibacterial activity of compounds **13**, **17**–**20**, and, particularly of compound **14**. The latter phenanthrene inhibited the proliferation of cervical cancer (HeLa), doxorubicin-sensitive colonic adenocarcinoma (COLO 205), and multidrug-resistant colonic adenocarcinoma (COLO 320/MDR-LRP) cell lines, with IC_50_ values ranging from 18.2 to 24.1 μM. Compounds **17**–**20** showed IC_50_ values ranging from 12.3 to 93.7 μM.^33^ In addition, compounds **14** and **17**–**20** exhibited strong synergism with doxorubicin.^33^ However, compound **13** demonstrated antiproliferative effects against SiHa cells with an IC_50_ value of 25.3 μM.^32^

**Table 5:** Antibacterial Activity of Phenanthrenes Isolated from *J. articulatus*^a^

	MIC							
	*E. coli*		*P. aeruginosa*		MSSA		MRSA	
Compound	(μg/mL)	(μM)	(μg/mL)	(μM)	(μg/mL)	(μM)	(μg/mL)	(μM)
**1**	>128	>413	>128	>413	128	413.0	128	413.0
**2**	>64	>121	>64	>121	32	60.5	32	60.5
**3**	>64	>229	>64	>229	32	114.3	32	114.3
**4**	>128	>435	>128	>435	64	217.5	64	217.5
**5**	>128	>457	>128	>457	128	457.0	128	457.0
**6**	>64	>230	>64	>230	16	57.5	32	115.0
**7**	>64	>121	>64	>121	32	60.5	64	121.0
**8**	>128	>243	>128	>243	64	121.5	64	121.5
**9**	>64	>121	>64	>121	32	60.5	32	60.5
**10**	NT	NT	NT	NT	NT	NT	NT	NT
**11**	>128	>481	>128	>481	128	481.0	128	481.0
**12**	>128	>484	>128	>484	4	15.1	4	15.1
**13**	>128	>485	>128	>485	32	121.3	32	121.3
**14**	>128	>488	>128	>488	4	15.3	4	15.3
**15**	>128	>454	>128	>454	16	56.8	16	56.8
**16**	>128	>458	>128	>458	64	229.0	64	229.0
**17**	>128	>457	>128	>457	16	57.1	16	57.1
**18**	>128	>480	>128	>480	8	30.0	8	30.0
**19**	>128	>454	>128	>454	32	113.5	32	113.5
**20**	>128	>242	>128	>242	32	60.5	32	60.5
ciprofloxacin	0.0078	0.02	0.125	0.37	0.125–0.25	0.37–0.74	0.125–0.25	0.37–0.74
vancomycin	256	176.7	2560	1766.7	1	0.69	2	1,38

a MIC: Minimum Inhibitory Concentration. MSSA: methicillin-susceptible *Staphylococcus aureus*. MRSA: methicillin-resistant *Staphylococcus aureus* (MRSA). NT: not tested.

Although, a clear correlation between structures and activities was not observed a preliminary structure–activity relationship analysis revealed that the double bond at C-9–C-10 increased the antibacterial activity, as observed when comparing compounds **1** and **4**, **11**, and **12**, and **13** and **14**. The hydroxylation of the CH_3_-11 in compound **19** was detrimental to the antibacterial effect compared to the activity observed for compound **18** (Table 5).

The monomeric phenanthrenes **12**, **14**, **15**, **17**, and **18** showed lower MIC values, ranging from 4 to 16 μg/mL, than the dimers **7**, **8**, **9**, and **20**, which displayed MIC values equal to or higher than 32 μg/mL (Table 5). This tendency was observed when comparing the MIC values of diphenanthrene **12** (MIC = 15.1 μM) and its monomers **8** and **9** (MIC = 121.5 and 60.5 μM, respectively). Similarly, diphenanthrene **20** showed an MIC value of 60.5 μM, formed by combining of compounds **12** and **18**, with the latter having an MIC value of 30.0 μM. The presence of an alkyne bond in compounds **6** and **14** instead of an alkene bond in compounds **3** and **12** had no impact on the activity.

Considering biofilms as protective barriers for bacteria against the controlling effects of antibiotics, new molecules targeting their establishment or eradicating preformed biofilms are greatly needed. Therefore, we investigated the antibiofilm properties of the isolated phenanthrenes, which showed effectiveness against planktonic *S. aureus*. The activity of the investigated phenanthrenes against sessile cells was evaluated by interfering with biofilm formation or eradication. Most of the tested compounds were observed to effectively prevent MSSA and MRSA biofilm formation, with compounds **12** and **14** being the most active, with a minimum biofilm prevention concentration (MBPC) value of 15.1 μM for **12**, and 15.3 μM for **14**, respectively (Table 6). In some cases, the active concentrations were higher than those observed for the inhibition of planktonic *S*. *aureus*. Notably, that this is the first time the antibiofilm activity of the known phenanthrenes **11**–**20**, including compounds **12** and **15** previously studied, has been described. The most potent compounds (**12** and **14**) with biofilm prevention effects were further evaluated for their ability to eradicate preformed biofilms; however, no effect was observed in this assay (Table 6).

**Table 6:** Effects of Isolated Compounds on Biofilm Formation and Disruption of Preformed Biofilms of MSSA and MRSA

	MBPC^a^				MBEC^b^			
	MSSA		MRSA		MSSA		MRSA	
Compound	(μg/mL)	(μM)	(μg/mL)	(μM)	(μg/mL)	(μM)	(μg/mL)	(μM)
**2**	64	121.0	128	242.0				
**3**	32	114.3	32	114.3				
**6**	32	115.0	32	114.3				
**9**	64	121.0	64	121.0				
**12**	4	15.1	4	15.1	>128	>484	>128	>484
**14**	4	15.3	4	15.3	>128	>488	>128	>488
**15**	32	113.5	32	113.5				
**17**	32	114.3	32	114.3				
**18**	16	60.0	16	60.0				
**19**	64	227.0	64	227.0				
**20**	32	60.5	32	60.5				
ciprofloxacin	0.25–0.5	0.75–1.51	0.5	1.51	>128^c^	>386.7	>128^c^	>386.7
vancomycin	1	0.69	2	1.38	>128	>88.3	>128	>88.3

a MBPC: minimum biofilm prevention concentration.

b MBEC: minimum biofilm eradication concentration.

c These values are in agreement to those found by Cruz et al. (2018).^41^

Our research led to the isolation of 20 phenanthrenes (**1**–**20**), including 10 new ones (**1**–**10**), and 6 other compounds from *J. articulatus*. Previously a disc diffusion assay established that the CHCl_3_ extract of the plant exhibited antibacterial activity against MRSA with a 9.3 mm inhibition zone.^39^ The antibacterial activity of the compounds isolated from this extract was tested against *E. coli*, *P. aeruginosa*, MSSA, and MRSA (Table 5). Compounds **15**, **17**, **18**, and particularly **12** and **14**, demonstrated the ability to inhibit the growth of sessile MSSA and the resistant MRSA strain. Furthermore, these compounds inhibited biofilm formation by these pathogens. We have provided preliminary SAR conclusions based on the identified target activities. Generally, phenanthrenes with a double bond at C-9–C-10 demonstrated stronger activity than dihydrophenanthrenes. Moreover, monophenanthrenes showed more potent effectiveness than diphenanthrenes.

## Experimental Section

### General Experimental Procedures

A Shimadzu UV-1800 spectrophotometer was used to obtain UV spectra. NMR spectra were recorded in MeOH-*d*_4_ and CDCl_3_ on a Bruker Avance DRX 500 spectrometer (Bruker, Germany) at 500 and 600 MHz (^1^H) and 125 and 150 MHz (^13^C). The signals of the deuterated solvents (δ_H_ 4.78 and 3.31, and δ_C_ 49.2 for CD_3_OD, and δ_H_ 7.26 and δ_C_ 77.16 for CDCl_3_) were used as reference. High-resolution MS spectra were acquired on a Thermo Scientific Q-Exactive Plus Orbitrap mass spectrometer equipped with ESI ion source in either positive or negative ionization mode. The data were acquired and processed using MassLynx software. HPLC was performed out on a Shimadzu HPLC using normal (Phenomenex, Kinetex Luna silica, 3 μm, 100 A, 150 × 4.6 mm) and RP (Phenomenex, Kinetex Phenyl-Hexyl, 5 μm 100 Å, 150 × 4.6 mm) columns. Vacuum liquid chromatography (VLC) was performed on silica gel (GF_254_, 15 μm, Merck, Darmstadt, Germany). Sephadex LH-20 (25–100 μm, Sigma-Aldrich, Budapest, Hungary) was used for gel filtration. Preparative thin-layer chromatography (prep. TLC) was performed on silica gel plates (TLC silica gel 60 F_254_, Merck, Darmstadt, Germany) and on reversed-phase (RP) silica gel plates (TLC silica gel 60 RP-18 F_254_, Merck, Darmstadt, Germany. All solvents used for column chromatographies were of analytical grade (VWR Ltd., Szeged, Hungary).

### Plant Material

*J. articulatus* L. (whole plant, 2.6 kg) was collected during the flowering period in June 2020 from the sandy area of the dried lakebed near Barcs, Hungary (GPS coordinates: 45° 58′ 30.817″ N, 17° 32′ 17.764″ E). Botanical identification of the plant material was conducted by Dragica Purger (Department of Pharmacognosy, University of Pécs, Hungary). A voucher specimen (No. 892) was deposited at the Department of Pharmacognosy, University of Szeged, Szeged, Hungary.

### Extraction and Isolation

The air-dried whole plant of *J. articulatus* (2.6 kg) was ground and percolated with MeOH (20 L) at room temperature. The MeOH extract was concentrated (350 g) under reduced pressure. The extract was dissolved in 50% aqueous MeOH after evaporation, and solvent–solvent partitions were performed using *n*-hexane (8 × 0.5 L), CHCl_3_ (10 × 0.5 L), and EtOAc (9 × 0.5 L).

The concentrated CHCl_3_-soluble fraction (20.5 g) was separated via VLC on silica gel using a gradient system of cyclohexane–EtOAc–MeOH [from 98:2:0 to 1:1:0 (1500 mL/eluent) and finally with MeOH; the volume of collected fractions was 100 mL]. The fractions were combined into 12 main fractions (Fr. 1–12) based on TLC analysis using UV light at 254 and 366 nm, and visualized with vanillin sulfuric acid reagent followed by heating at 110 °C for 1 min.

Almost all fractions (Fr. 1–11) were purified by Sephadex LH-20 gel chromatography using CH_2_Cl_2_–MeOH (1:1) as the eluent. Fraction 12 was separated via VLC on silica gel with a gradient system of CHCl_3_–MeOH [from 1:0 to 0:1 (200 mL/eluent); the volume of the collected fractions was 10 mL] yielding 12 fractions (Fr. 12/1–12/12). The fractions were combined based on their TLC patterns. Fraction 12/12 was pure and yielded luteolin (306.4 mg). Fraction 2/2 was separated by normal-phase (NP)-HPLC under gradient conditions, using cyclohexane–EtOAc (from 80:20 to 55:45 in 10 min; flow rate 1 mL/min) as the mobile phase, yielding vanillin (*t*_R_ = 9.5 min, 33.2 mg). Fraction 2/3 was purified by RP-HPLC under gradient conditions using MeOH–H_2_O (from 82:18 to 85:15 in 10 min; flow rate 1 mL/min) as the mobile phase, yielding compounds **10** (0.8 mg, *t*_R_ = 6.4 min) and **20** (2.9 mg, *t*_R_ = 6.9 min). Fraction 3/3 was separated by prep. TLC on silica gel using cyclohexane–EtOAc–EtOH (60:30:3) as a solvent system. Subfraction 3/3/1 was further purified by RP-HPLC using MeOH–H_2_O gradient solvent system (from 8:2 to 1:0 in 9 min; flow rate 1 mL/min) as the mobile phase, yielding compound **8** (0.9 mg, *t*_R_ = 6.8 min). Fraction 4/3 was purified by RP-HPLC under gradient conditions using MeOH–H_2_O (from 5:5 to 1:0 in 10 min; flow rate 1 mL/min) as the mobile phase, yielding two subfractions. Fraction 4/3/2 was pure, resulting compound **1** (7.6 mg, *t*_R_ = 8.8 min). Fraction 4/4 was purified by NP-HPLC under isocratic conditions using cyclohexane–EtOAc (82:18 for 16 min; flow rate 1 mL/min) as the mobile phase. Subfraction 4/4/1 was further purified by RP-HPLC using MeCN–H_2_O solvent system (from 7:3 to 85:15 in 6 min; flow rate 1 mL/min) as the mobile phase, to yield compound **18** (1.2 mg, *t*_R_ = 2.5 min,) and compound **9** (1.5 mg, *t*_R_ = 4.2 min). Fraction 5/2 was purified by NP-HPLC using a cyclohexane–EtOAc solvent system (from 75:25 to 1:1 in 6 min; flow rate 1 mL/min) as the mobile phase, yielding compound **15** (3.3 mg, *t*_R_ = 10.3 min). Subfraction 5/2/1 was further separated by RP-HPLC under gradient conditions using MeCN–H_2_O (from 4:6 to 1:0 in 8 min; flow rate 1 mL/min) as the mobile phase, yielding *p*-hydroxybenzaldehyde (3.4 mg, *t*_R_ = 2.16 min). Fraction 5/3 was separated by prep. TLC on silica gel using cyclohexane–EtOAc–EtOH (60:30:3) as the solvent system, yielding compound **11** (100.6 mg). Fraction 6/5 was purified by prep. TLC on silica gel using cyclohexane–EtOAc–EtOH (60:30:3) as the solvent system. Subfraction 6/5/1 was further purified by RP-HPLC using a MeOH–H_2_O gradient (from 8:2 to 1:0 in 4 min; flow rate 1 mL/min) as the mobile phase, yielding compound **2** (1.4 mg, *t*_R_ = 7.10 min,). Fraction 6/6 was separated by RP-HPLC using a MeOH–H_2_O gradient (from 75:25 to 8:2 in 8 min; flow rate 1 mL/min), yielding compound **13** (3.4 mg, *t*_R_ = 4.8 min). Fraction 7/4 was purified by RP-HPLC using a gradient of MeCN–H_2_O (from 4:6 to 8:2 in 10 min; flow rate 1 mL/min) as the mobile phase to yield compound **16** (4.2 mg *t*_R_ = 6.7 min). Furthermore, fraction 7/5 was purified by the same RP-HPLC method used for fraction 7/4, yielding compound **7** (0.8 mg, *t*_R_ = 11.8 min). Fraction 7/6 was separated by prep. TLC on silica gel using cyclohexane–EtOAc–EtOH (60:30:3) as the solvent system to yield compound **12** (135.7 mg).

After gel filtration of fraction 9 on Sephadex LH-20, fraction 9/6 was pure and yielded compound **14** (5.8 mg). Fraction 9/2 was separated by prep. TLC on silica gel using CHCl_3_–MeOH (95:5) as the mobile phase, and subfraction 9/2/1 was further purified by RP-HPLC using MeOH–H_2_O gradient solvent system (from 1:1 to 1:0 in 7 min; flow rate 1 mL/min) to yield dehydrovomifoliol (1.5 mg, *t*_R_ = 3.5 min). Fraction 9/5 underwent further separation by RP-HPLC under gradient conditions using MeCN–H_2_O (from 4:6 to 1:0 in 9 min; flow rate 1 mL/min) as the mobile phase, yielding compounds **17** (3.9 mg, *t*_R_ = 3.7 min) and **5** (0.9 mg, *t*_R_ = 4.8 min). Fraction 10/4 was separated by prep. TLC on RP silica gel using MeOH–H_2_O (7:3) as the mobile phase, yielding two subfractions. Furthermore, subfraction 10/4/2 was further purified by RP-HPLC under gradient conditions, using MeOH–H_2_O (from 7:3 to 85:15 in 10 min; flow rate 1 mL/min) as the mobile phase, yielding compounds **3** (10.8 mg, *t*_R_ = 3.76 min) and **4** (1.3 mg, *t*_R_ = 5.9 min). Fraction 10/5 was purified by RP-HPLC using MeOH–H_2_O solvent system (from 65:35 to 75:25 in 9 min; flow rate 1 mL/min), and compound **6** (3.1 mg, *t*_R_ = 4.15 min) was isolated. In addition, fraction 11/5 was purified by RP-HPLC using a gradient solvent system comprising MeOH–H_2_O (from 65:35 to 8:2 in 8 min; flow rate 1 mL/min), yielding apigenin (1.0 mg, *t*_R_ = 4.1 min) and compound **19** (1.1 mg, *t*_R_ = 4.62 min). Fraction 12/4 was separated by prep. TLC on RP silica gel using MeOH–H_2_O (8:2) as the mobile phase, and subfraction 12/4/1 underwent further purification through RP-HPLC using an isocratic MeOH–H_2_O (48:52) eluent, at a flow rate of 1 mL/min, yielding 1,3-*O*-diferuloylglycerol (1.9 mg, *t*_R_ = 21.50 min).

#### Articulin A (**1**)

Light-yellow amorphous solid; UV (MeOH) λ_max_ (log ε) = 213 (4.35), 282 (4.08), 320 (3.82) nm; ^1^H and ^13^C NMR data, see Table 1; (−)-HRESIMS *m*/*z* 295.1340 [M–H]^−^ (calcd for C_19_H_19_O_3_, 295.1342)

#### Articulin B (**2**)

Light-yellow amorphous solid; UV (MeOH) λ_max_ (log ε) = 240 (4.12), 321 (3.46) nm; ^1^H and ^13^C NMR data, see Table 1; (−)-HRESIMS *m*/*z* 529.2378 [M–H]^−^ (calcd for C_36_H_33_O_4_, 529.2384).

#### Articulin C (**3**)

Yellow amorphous solid; UV (MeOH) λ_max_ (log ε) = 228 (4.08), 264 (4.19), 348 (2.81), 365 (2.85)nm; ^1^H and ^13^C NMR data, see Table 1; (+)-HRESIMS *m*/*z* 281.1173 [M+H]^+^ (calcd for C_18_H_17_O_3_, 281.1172).

#### Articulin D (**4**)

Light-yellow amorphous solid; UV (MeOH) λ_max_ (log ε) = 228 (4.14), 267 (4.23), 350 (2.80), 367 (2.84) nm; ^1^H and ^13^C NMR data, see Table 2; (−)-HRESIMS *m*/*z* 293.1188 [M–H]^−^ (calcd for C_19_H_17_O_3_, 293.1183).

#### Articulin E (**5**)

Light-yellow amorphous solid; UV (MeOH) λ_max_ (log ε) = 229 (3.85), 262 (3.95), 347 (2.53), 364 (2.56) nm; ^1^H and ^13^C NMR data, see Table 2; (+)-HRESIMS *m*/*z* 281.1175 [M+H]^+^ (calcd for C_18_H_17_O_3_, 281.1172).

#### Articulin F (**6**)

Yellow amorphous solid; UV (MeOH) λ_max_ (log ε) = 230 (4.44), 269 (4.63), 360 (3.91), 375 (3.86) nm; ^1^H and ^13^C NMR data, see Table 2; (−)-HRESIMS *m*/*z* 277.0870 [M–H]^−^ (calcd for C_18_H_13_O_3_, 277.0870).

#### Articulin G (**7**)

Light-yellow amorphous solid; UV (MeOH) λ_max_ (log ε) = 230 (3.66), 270 (3.74), 367 (2.26) nm; ^1^H and ^13^C NMR data, see Table 3; (−)-HRESIMS *m*/*z* 529.2021 [M–H]^−^ (calcd for C_35_H_29_O_5_, 529.2021).

#### Articulin H (**8**)

Light-yellow amorphous solid; UV (MeOH) λ_max_ (log ε) = 224 (4.09), 266 (4.06), 365 (2.51) nm; ^1^H and ^13^C NMR data, see Table 3; (−)-HRESIMS *m*/*z* 525.2071 [M–H]^−^ (calcd for C_36_H_29_O_4_, 525.2071).

#### Articulin I (**9**)

Light-yellow amorphous solid; UV (MeOH) λ_max_ (log ε) = 225 (4.21), 267 (4.29), 348 (2.78), 366 (2.82) nm; ^1^H and ^13^C NMR data, see Table 4; (−)-HRESIMS *m*/*z* 525.2074 [M–H]^−^ (calcd for C_36_H_29_O_4_, 525.2071).

#### Articulin J (**10**)

Yellow amorphous solid; UV (MeOH) λ_max_ (log ε) = 223 (3.92), 270 (3.83), 367 (2.48) nm; ^1^H and ^13^C NMR data, see Table 4; (−)-HRESIMS *m*/*z* 527.2224 [M–H]^−^ (calcd. for C_36_H_31_O_4_, 527.2222).

#### Dehydrojuncuenin B (**12**)

^1^H NMR (500 MHz, MeOH-*d*_4_) δ 8.25 d (1H, d, *J* = 8.9 Hz, H-4), 7.85 (1H, s, H-5), 7.83 (1H, d, *J* = 9.5 Hz, H-9), 7.57 (1H, d, *J* = 9.5 Hz, H-10), 7.12 (1H, d, *J* = 8.9 Hz, H-3), 7.00 (1H, dd, *J* = 17.9, 11.4 Hz, H-13), 5.73 (1H, dd, *J* = 11.4, 2.2 Hz, H-14a), 5.30 (1H, dd, *J* = 17.9, 2.2 Hz), 2.48 (3H, s, 11-CH_3_), and 2.32 (3H, s, 12-CH_3_); ^13^C NMR (125 MHz, MeOH-*d*_4_) δ 155.7 (C-6), 154.0 (C-2), 138.5 (C-8), 136.3 (C-13), 133.4 (C-1a), 131.9 (C-5a), 125.4 (C-9), 124.8 (C-4a), 123.7 (C-7), 123.6 (C-8a), 122.3 (C-4), 121.4 (C-14), 120.1 (C-10), 118.3 (C-1), 116.8 (C-3), 105.4 (C-5), 13.6 (C-12), and 11.0 (C-11).

### Antibacterial Activity

Compounds were tested against medically relevant bacterial strains, including *E. coli* ATCC 25922 and *P. aeruginosa* PAO1, selected as models for Gram-negative bacteria. Furthermore, MSSA (ATCC 6538) and MRSA (ATCC 33592) were used as models for Gram-positive strains. All cells were grown at 37 °C in Luria–Bertani media for 16 h with orbital shaking. Overnight cultures were then utilized and dissolved in Müller–Hinton media.

### Planktonic Bacteria Susceptibility Assays

Bacterial suspensions adjusted to 1 × 10^6^ CFU/mL were exposed, in triplicate, to varying concentrations of each target compound in wells containing Müller–Hinton medium. The compounds dissolved in filtered EtOH were added to achieve final concentrations ranging from 1 to 128 μg/mL. Plates were subsequently incubated at 37 °C with constant stirring for 24 h. Absorbance at 595 nm was measured using a Synergy HTX multimodal microplate reader (BioTek Instruments, Inc., USA) at the end of the incubation period. Ciprofloxacin and vancomycin were simultaneously used as positive controls. Moreover, MIC values were interpreted as the lowest concentrations exhibiting 90% or greater growth inhibition than the negative control comprising medium and 1% EtOH. Notably, no difference in growth was observed between these cells and viability control bacteria grown solely in Müller–Hinton medium. The results obtained from a minimum of three independent experiments were averaged.

### Reversion of Biofilm Formation and Eradication of Preformed Biofilm Assays

To determine whether the active compounds inhibit *S. aureus* biofilm formation and eradicate preformed biofilms, preexposure and postexposure experiments were performed, as previously described, with some modifications.^42,43^ Briefly, 96-well plates containing Luria–Bertani media and bacterial inoculum corresponding to 1 × 10^6^ CFU/mL cells harvested from exponentially growing cultures were incubated with compounds **2**, **3**, **6**, **9**, **12**, **14**, **15**, and **17**–**20** at 1 to 128 μg/mL. This incubation was at 37 °C for 24 h under static conditions to allow biofilm formation. Similarly, to determine the biofilm eradication ability of compounds **12** and **14**, which showed the highest effect in the previous experiment, the formed biofilms were treated with both molecules and incubated for an additional 24 h at 37 °C. Ciprofloxacin and vancomycin simultaneously served as positive controls. Following the treatment period, the supernatants were carefully discarded. For biofilm fixation, 200 μL per well of pure EtOH was added for 20 min. The cells were then stained with 200 μL of 0.1% crystal violet (H_2_O–MeOH 4:1) for 5 min and washed four times with sterile phosphate-buffered saline (PBS) solution to remove detached cells. The stained bacteria were suspended in 100 μL of EtOH, and absorbance was measured at 595 nm using a Synergy HTX multimodal microplate reader (BioTek Instruments, Inc., USA). Both MBPC and minimum biofilm eradication concentration (MBEC) were determined as the lowest concentrations inducing at least 90% growth inhibition compared to the negative control containing only the dissolution solvent. No difference was observed in the growth of the former cells compared to the viability control group containing only Luria–Bertani broth. At least three independent experiments were conducted, and the results were averaged.

## Data Availability

The NMR data for compounds **1**–**10** have been deposited in the Natural Products Magnetic Resonance Database (NP-MRD; www.np-mrd.org) and can be found at NP0333257 (**1**), NP0333258 (**2**), NP0333259 (**3**), NP0333260 (**4**), NP0333261 (**5**), NP0333262 (**6**), NP0333263 (**7**), NP0333264 (**8**), NP0333265 (**9**), and NP0333266 (**10**).

## Supporting Information Available

NMR, HRESIMS, and UV spectra for compounds **1**–**10** (PDF)
