# Supplementary material for: Phenanthrenes from Juncus articulatus with Antibacterial and Biofilm Formation Inhibitory Activity
Source: J Nat Prod. 2024 Aug 9;87(8):2068–80. doi: 10.1021/acs.jnatprod.4c00577 (PMC11348428; doi:10.1021/acs.jnatprod.4c00577)
Supplement: Supplementary file 1 — np4c00577_si_001.pdf [file np4c00577_si_001.pdf]

SUPPORTING INFORMATION

Phenanthrenes from *Juncus articulatus* with Antibacterial and Biofilm Formation Inhibitory Activity

Anita Barta, Agostina Salusso, Norbert Kúsz, Róbert Berkecz, Jan Schlauer, Dragica Purger, Judit Hohmann, Cecilia Carpinella,* Andrea Vasas*

Corresponding Authors

Andrea Vasas – *Institute of Pharmacognosy, University of Szeged, 6720 Szeged, Hungary; HUN-REN-USZ Biologically Active Natural Products Research Group, University of Szeged, Eötvös u. 6, H-6720 Szeged, Hungary;* orcid.org/0000-0002-1818-7702; E-mail: vasas.andrea@szte.hu

Cecilia Carpinella – *Fine Chemical and Natural Products Laboratory, CIDIE CONICET-UCC, Universidad Católica de Córdoba, Córdoba, Argentina*; orcid.org/0000-0001-5553-1851; E-mail: ceciliacarpinella@ucc.edu.ar.

Authors

Anita Barta – *Institute of Pharmacognosy, University of Szeged, 6720 Szeged, Hungary; HUN-REN-USZ Biologically Active Natural Products Research Group, University of Szeged, Eötvös u. 6, H-6720 Szeged, Hungary*

Agostina Salusso – *Fine Chemical and Natural Products Laboratory, CIDIE CONICET-UCC, Universidad Católica de Córdoba, Córdoba, Argentina*

Norbert Kúsz – *Institute of Pharmacognosy, University of Szeged, 6720 Szeged, Hungary*

Róbert Berkecz – *Institute of Pharmaceutical Analysis, University of Szeged, 6720 Szeged, Hungary*

Jan Schlauer – *The Center for Plant Molecular Biology, University of Tuebingen, D-72076 Tuebingen, Germany*

Dragica Purger – *Department of Pharmacognosy, University of Pécs, 7624 Pécs, Hungary*

Judit Hohmann – *Institute of Pharmacognosy, University of Szeged, 6720 Szeged, Hungary; HUN-REN-USZ Biologically Active Natural Products Research Group, University of Szeged, Eötvös u. 6, H-6720 Szeged, Hungary*

TABLE OF CONTENTS

Figure S1. ^{1}H NMR spectrum of compound **1** (500 MHz, in CD_3OD). 6

Figure S2. ^{13}C (JMOD) NMR spectrum of compound **1** (125 MHz, in CD_3OD). 6

Figure S3. HSQC spectrum of compound **1** (in CD_3OD). 7

Figure S4. ^{1}H-^{1}H COSY spectrum of compound **1** (in CD_3OD). 7

Figure S5. HMBC spectrum of compound **1** (in CD_3OD). 8

Figure S6. NOESY spectrum of compound **1** (in CD_3OD). 8

Figure S7. UV spectrum of compound **1** in MeOH. 9

Figure S8. (–)-HRESIMS spectrum of compound **1**. 9

Figure S9. ^{1}H NMR spectrum of compound **2** (500 MHz, in $CDCl_3$). 10

Figure S10. ^{13}C (JMOD) NMR spectrum of compound **2** (125 MHz, in $CDCl_3$). 10

Figure S11. HSQC spectrum of compound **2** (in $CDCl_3$). 11

Figure S12. ^{1}H-^{1}H COSY spectrum of compound **2** (in $CDCl_3$). 11

Figure S13. HMBC spectrum of compound **2** (in $CDCl_3$). 12

Figure S14. NOESY spectrum of compound **2** (in $CDCl_3$). 12

Figure S15. UV spectrum of compound **2** in MeOH. 13

Figure S16. (–)-HRESIMS spectrum of compound **2**. 13

Figure S17. ^{1}H NMR spectrum of compound **3** (500 MHz, in CD_3OD). 14

Figure S18. ^{13}C (JMOD) NMR spectrum of compound **3** (125 MHz, in CD_3OD). 14

Figure S19. HSQC spectrum of compound **3** (in CD_3OD). 15

Figure S20. ^{1}H-^{1}H COSY spectrum of compound **3** (in CD_3OD). 15

Figure S21. HMBC spectrum of compound **3** (in CD_3OD). 16

Figure S22. NOESY spectrum of compound **3** (in CD_3OD). 16

Figure S23. UV spectrum of compound **3** in MeOH. 17

Figure S24. (+)-HRESIMS spectrum of compound **3**. 17

Figure S25. ^{1}H NMR spectrum of compound **4** (500 MHz, in CD_3OD). 18

Figure S26. ^{13}C (JMOD) NMR spectrum of compound **4** (125 MHz, in CD_3OD). 18

Figure S27. HSQC spectrum of compound **4** (in CD_3OD). 19

Figure S28. ^{1}H-^{1}H COSY spectrum of compound **4** (in CD_3OD). 19

Figure S29. HMBC spectrum of compound **4** (in CD_3OD). 20

Figure S30. NOESY spectrum of compound **4** (in CD_3OD). 20

Figure S31. UV spectrum of compound **4** in MeOH. 21

Figure S32. (–)-HRESIMS spectrum of compound **4**. 21

Figure S33. ^{1}H NMR spectrum of compound **5** (500 MHz, in CD_3OD). 22

Figure S34. ^{13}C (JMOD) NMR spectrum of compound **5** (125 MHz, in CD_3OD). 22

Figure S35. HSQC spectrum of compound **5** (in CD_3OD). 23
Figure S36. ^1H-^1H COSY spectrum of compound **5** (in CD_3OD). 23
Figure S37. HMBC spectrum of compound **5** (in CD_3OD). 24
Figure S38. NOESY spectrum of compound **5** (in CD_3OD). 24
Figure S39. UV spectrum of compound **5** in MeOH. 25
Figure S40. (+)-HRESIMS spectrum of compound **5**. 25
Figure S41. ^1H NMR spectrum of compound **6** (500 MHz, in CD_3OD). 26
Figure S42. ^{13}C (JMOD) NMR spectrum of compound **6** (125 MHz, in CD_3OD). 26
Figure S43. HSQC spectrum of compound **6** (in CD_3OD). 27
Figure S44. ^1H-^1H COSY spectrum of compound **6** (in CD_3OD). 27
Figure S45. HMBC spectrum of compound **6** (in CD_3OD). 28
Figure S46. NOESY spectrum of compound **6** (in CD_3OD). 28
Figure S47. UV spectrum of compound **6** in MeOH. 29
Figure S48. (–)-HRESIMS spectrum of compound **6**. 29
Figure S49. ^1H NMR spectrum of compound **7** (600 MHz, in CD_3OD). 30
Figure S50. ^{13}C (JMOD) NMR spectrum of compound **7** (150 MHz, in CD_3OD). 30
Figure S51. HSQC spectrum of compound **7** (in CD_3OD). 31
Figure S52. ^1H-^1H COSY spectrum of compound **7** (in CD_3OD). 31
Figure S53. HMBC spectrum of compound **7** (in CD_3OD). 32
Figure S54. NOESY spectrum of compound **7** (in CD_3OD). 32
Figure S55. UV spectrum of compound **7** in MeOH. 33
Figure S56. (–)-HRESIMS spectrum of compound **7**. 33
Figure S57. ^1H NMR spectrum of compound **8** (600 MHz, in CD_3OD). 34
Figure S58. ^{13}C (JMOD) NMR spectrum of compound **8** (150 MHz, in CD_3OD). 34
Figure S59. HSQC spectrum of compound **8** (in CD_3OD). 35
Figure S60. ^1H-^1H COSY spectrum of compound **8** (in CD_3OD). 35
Figure S61. HMBC spectrum of compound **8** (in CD_3OD). 36
Figure S62. NOESY spectrum of compound **8** (in CD_3OD). 36
Figure S63. UV spectrum of compound **8** in MeOH. 37
Figure S64. (–)-HRESIMS spectrum of compound **8**. 37
Figure S65. ^1H NMR spectrum of compound **9** (600 MHz, in CD_3OD). 38
Figure S66. ^{13}C (JMOD) NMR spectrum of compound **9** (150 MHz, in CD_3OD). 38
Figure S67. HSQC spectrum of compound **9** (in CD_3OD). 39
Figure S68. ^1H-^1H COSY spectrum of compound **9** (in CD_3OD). 39
Figure S69. HMBC spectrum of compound **9** (in CD_3OD). 40
Figure S70. NOESY spectrum of compound **9** (in CD_3OD). 40

Figure S71. UV spectrum of compound **9** in MeOH. 41
Figure S72. (–)-HRESIMS spectrum of compound **9**. 41
Figure S73. ^{1}H NMR spectrum of compound **10** (600 MHz, in CD_3OD). 42
Figure S74. ^{13}C (JMOD) NMR spectrum of compound **10** (150 MHz, in CD_3OD). 42
Figure S75. HSQC spectrum of compound **10** (in CD_3OD). 43
Figure S76. ^{1}H-^{1}H COSY spectrum of compound **10** (in CD_3OD). 43
Figure S77. HMBC spectrum of compound **10** (in CD_3OD). 44
Figure S78. NOESY spectrum of compound **10** (in CD_3OD). 44
Figure S79. UV spectrum of compound **10** in MeOH. 45
Figure S80. (–)-HRESIMS spectrum of compound **10**. 45

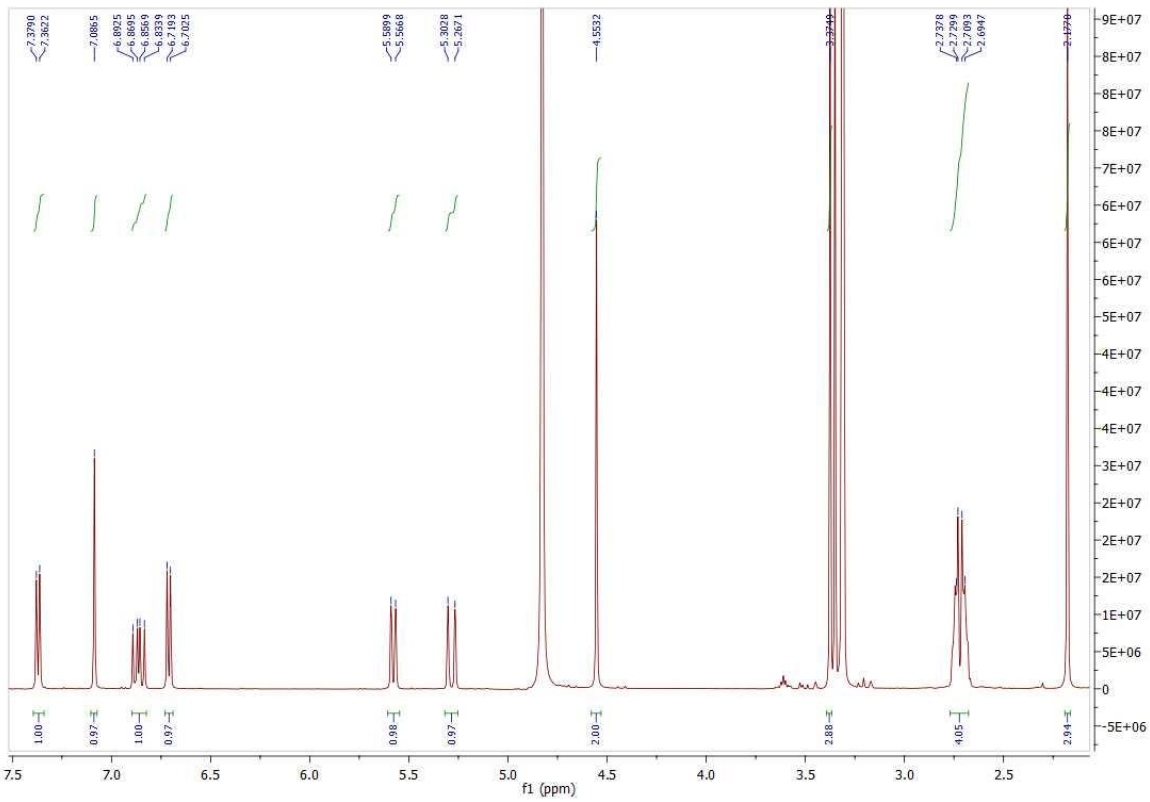

Figure S1. ^{1}H NMR spectrum of compound **1** (500 MHz, in CD_3OD).

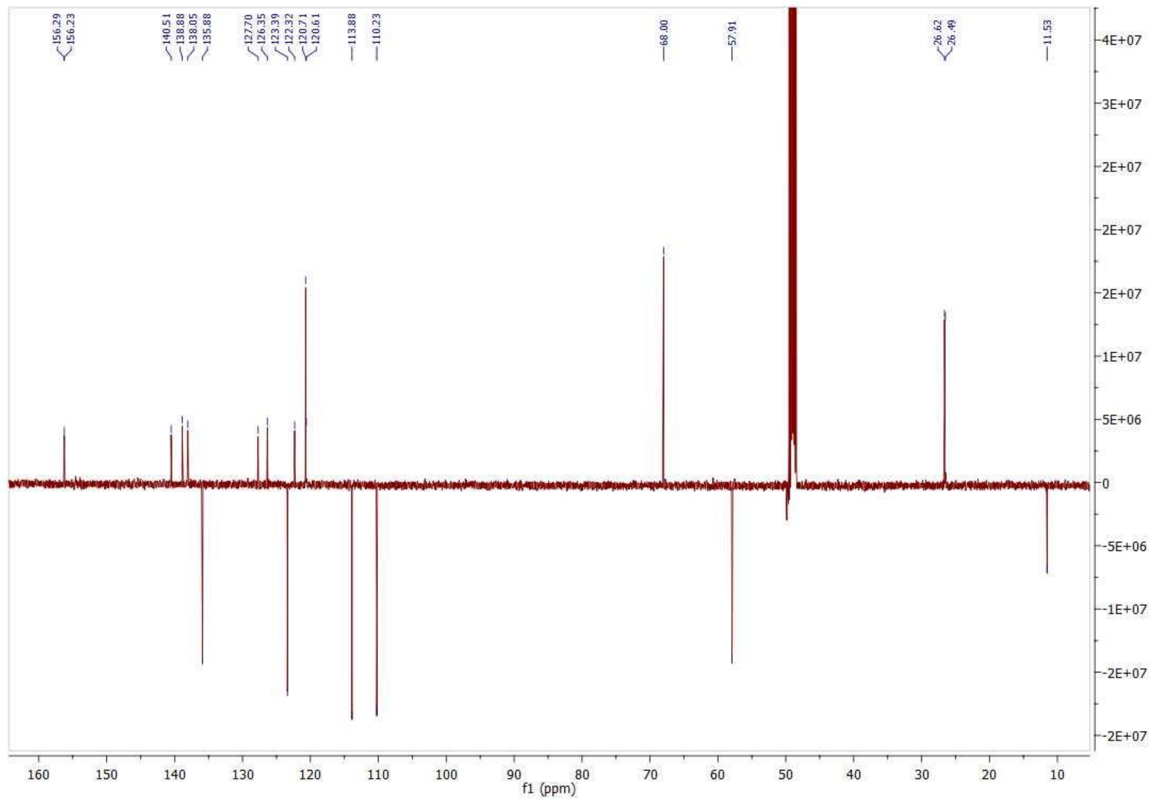

Figure S2. ^{13}C (JMOD) NMR spectrum of compound **1** (125 MHz, in CD_3OD).

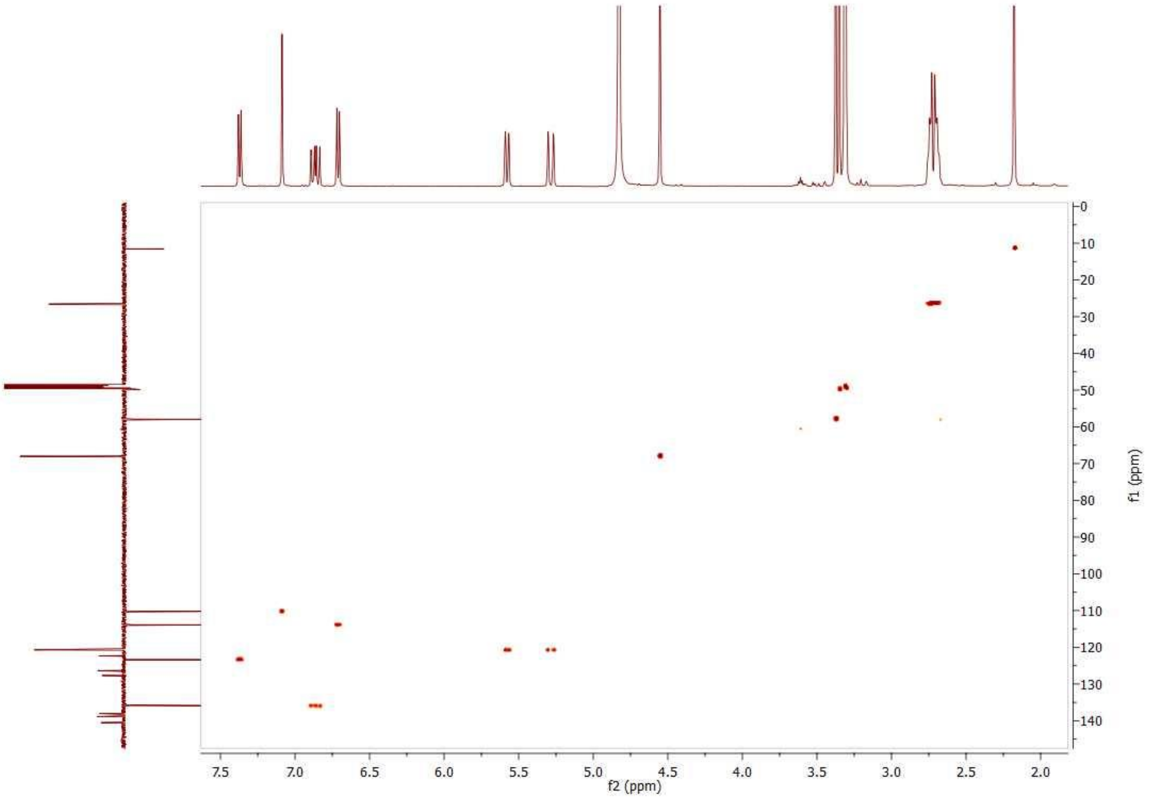

Figure S3. HSQC spectrum of compound **1** (in CD_3OD).

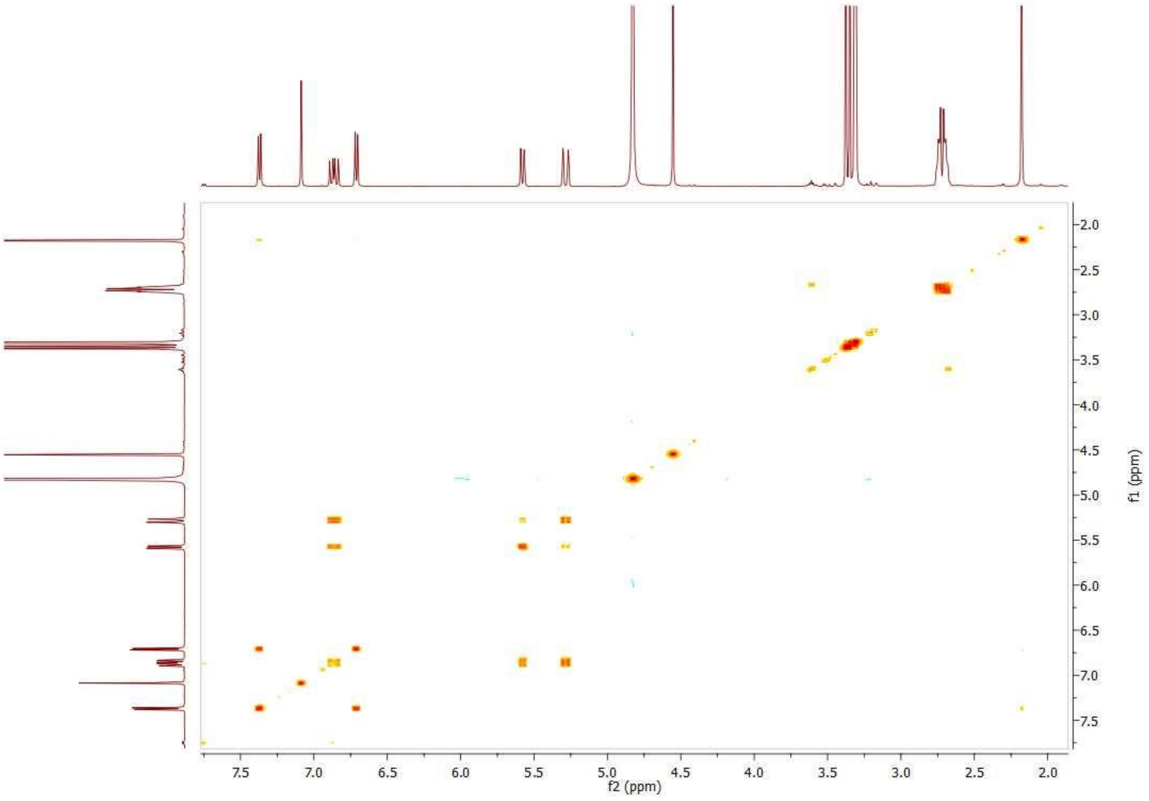

Figure S4. ^1H-^1H COSY spectrum of compound **1** (in CD_3OD).

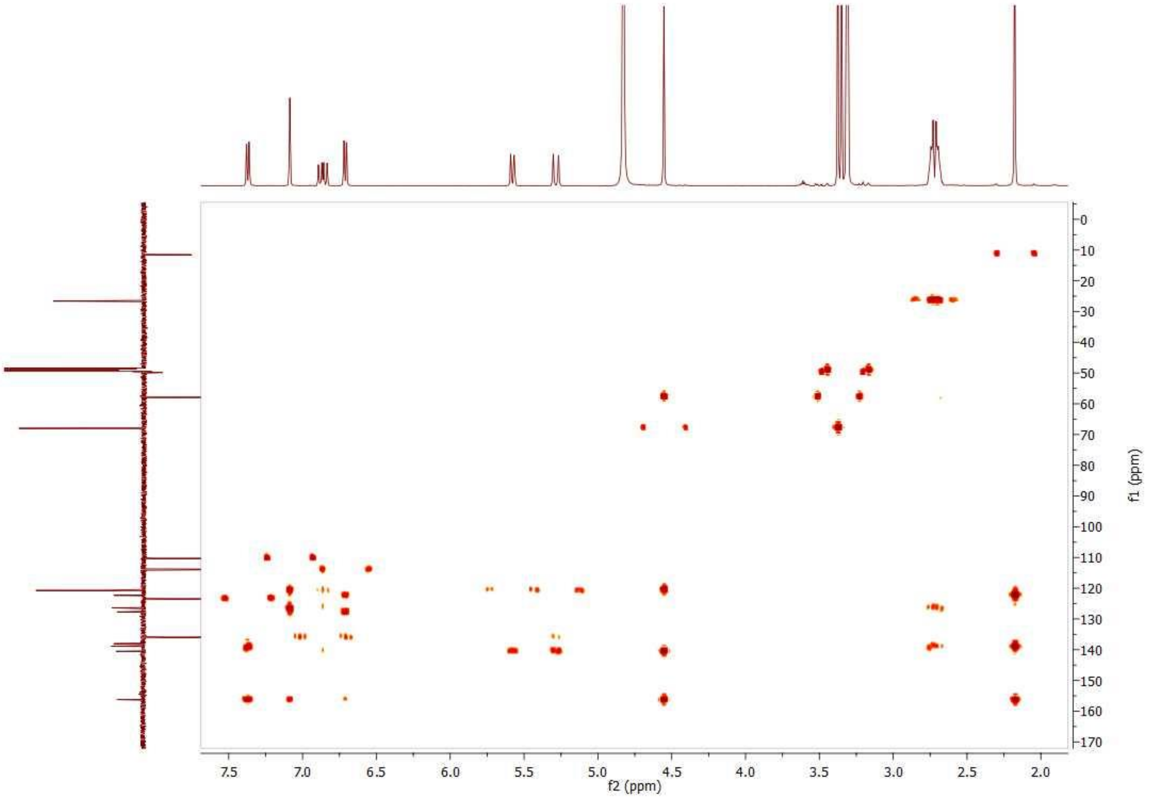

Figuráé S5. HMBC spectrum of compound **1** (in CD_3OD).

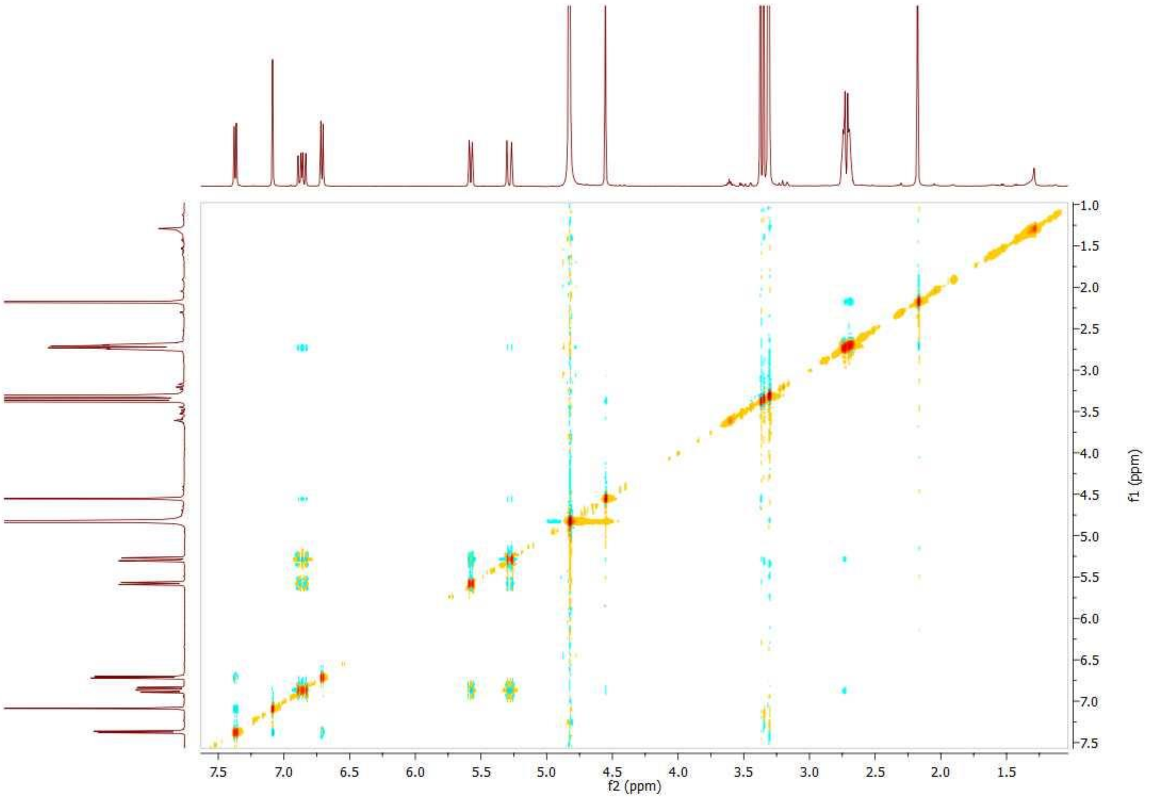

Figure S6. NOESY spectrum of compound **1** (in CD_3OD).

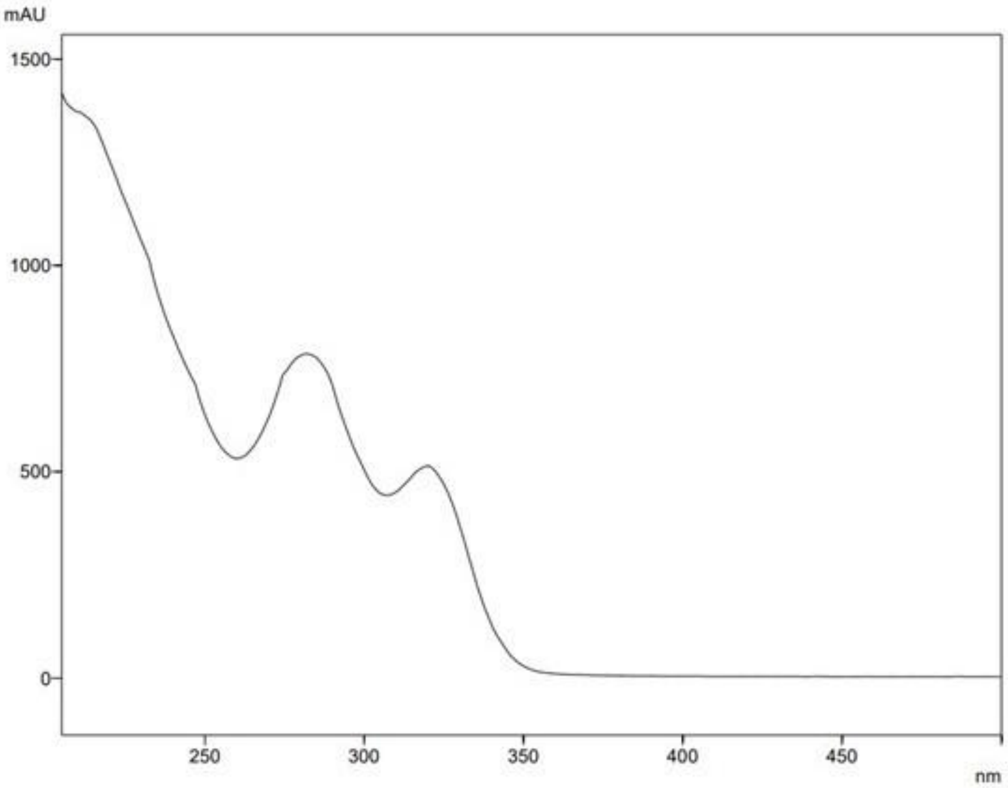

Figure S7. UV spectrum of compound **1** in MeOH.

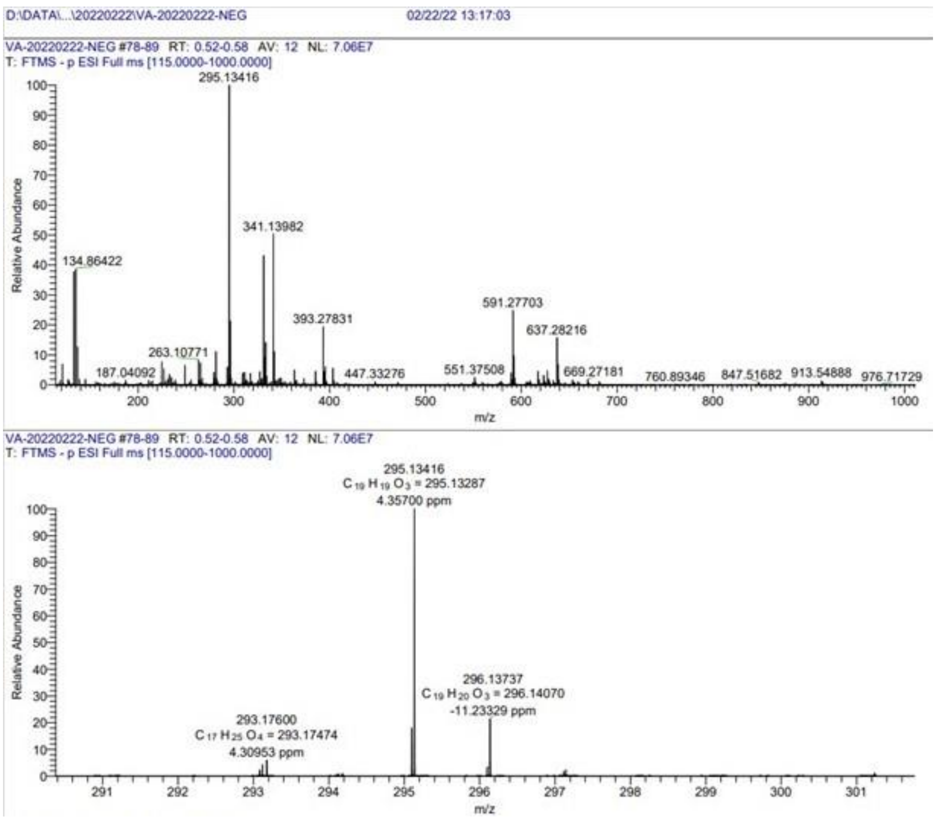

Figure S8. (–)-HRESIMS spectrum of compound **1**.

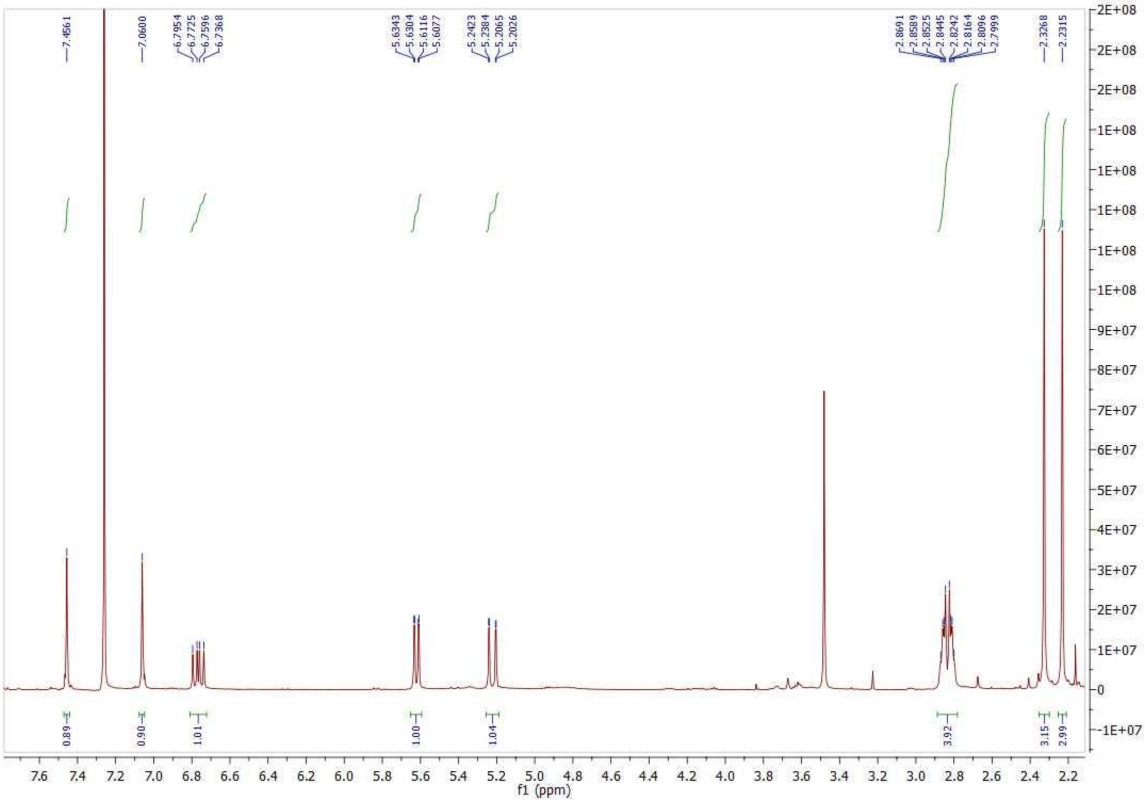

Figure S9. ^{1}H NMR spectrum of compound **2** (500 MHz, in $CDCl_3$).

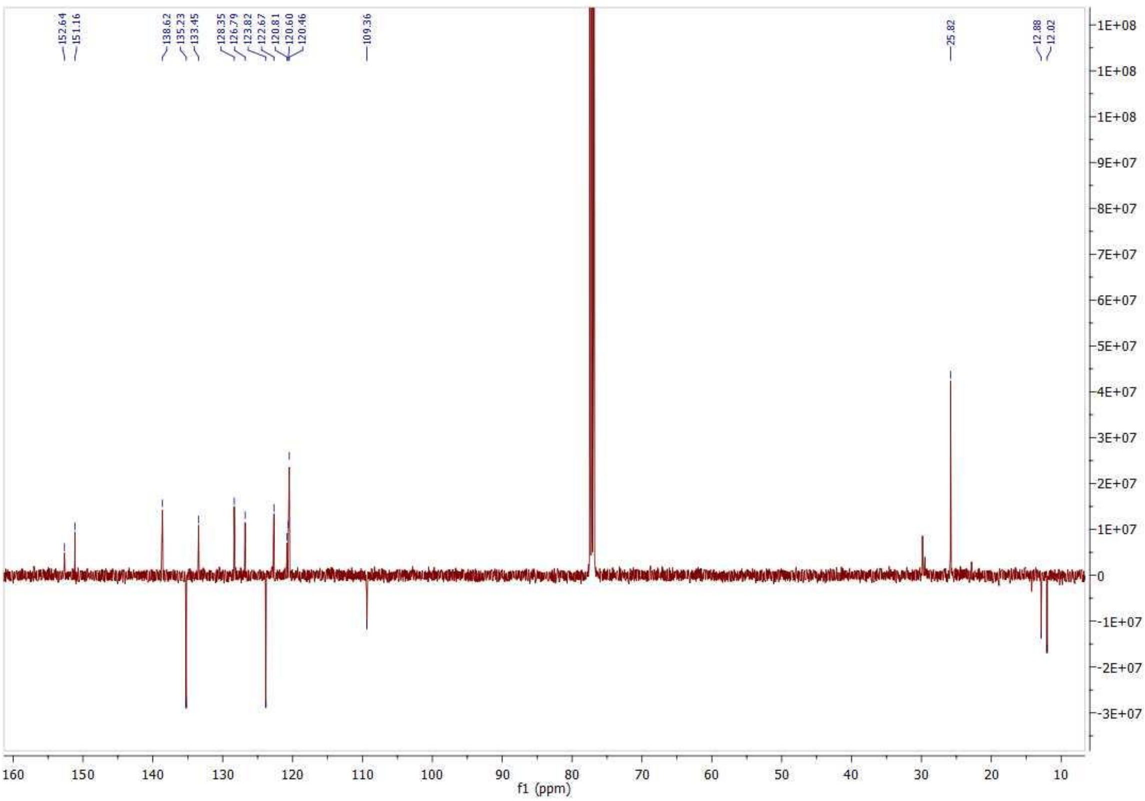

Figure S10. ^{13}C (JMOD) NMR spectrum of compound **2** (125 MHz, in $CDCl_3$).

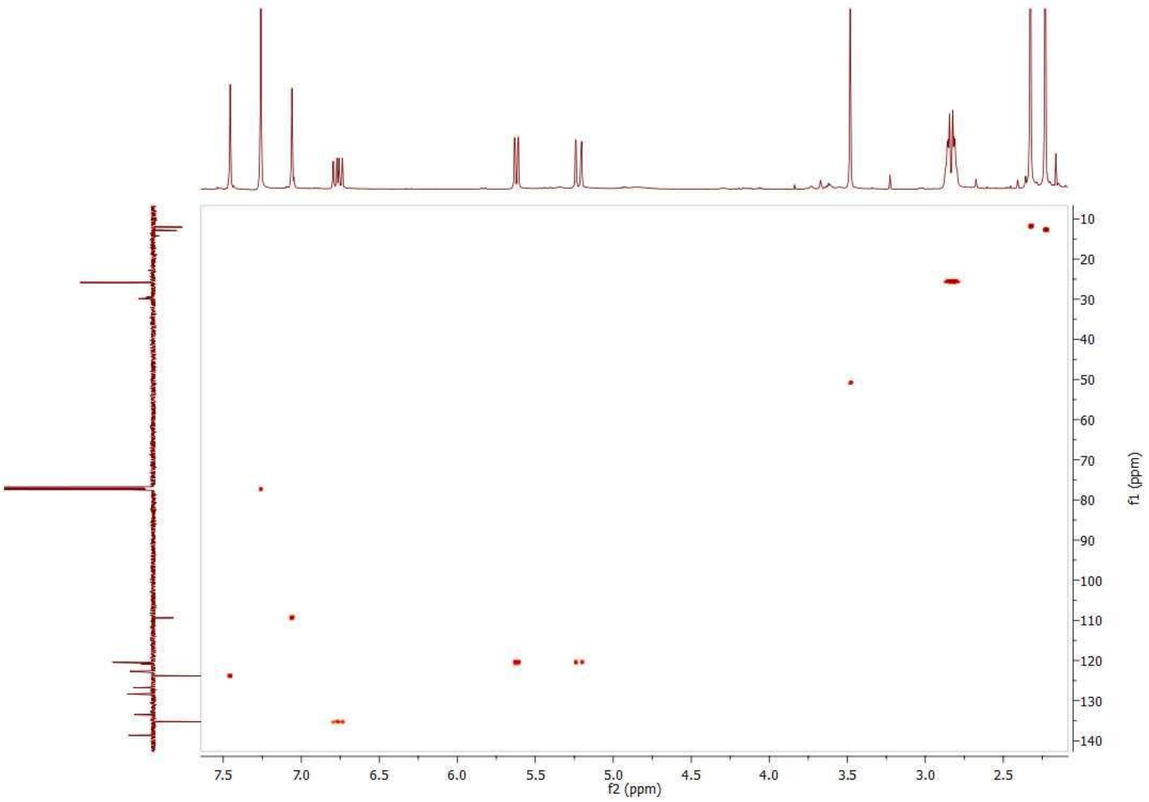

Figure S11. HSQC spectrum of compound **2** (in $CDCl_3$).

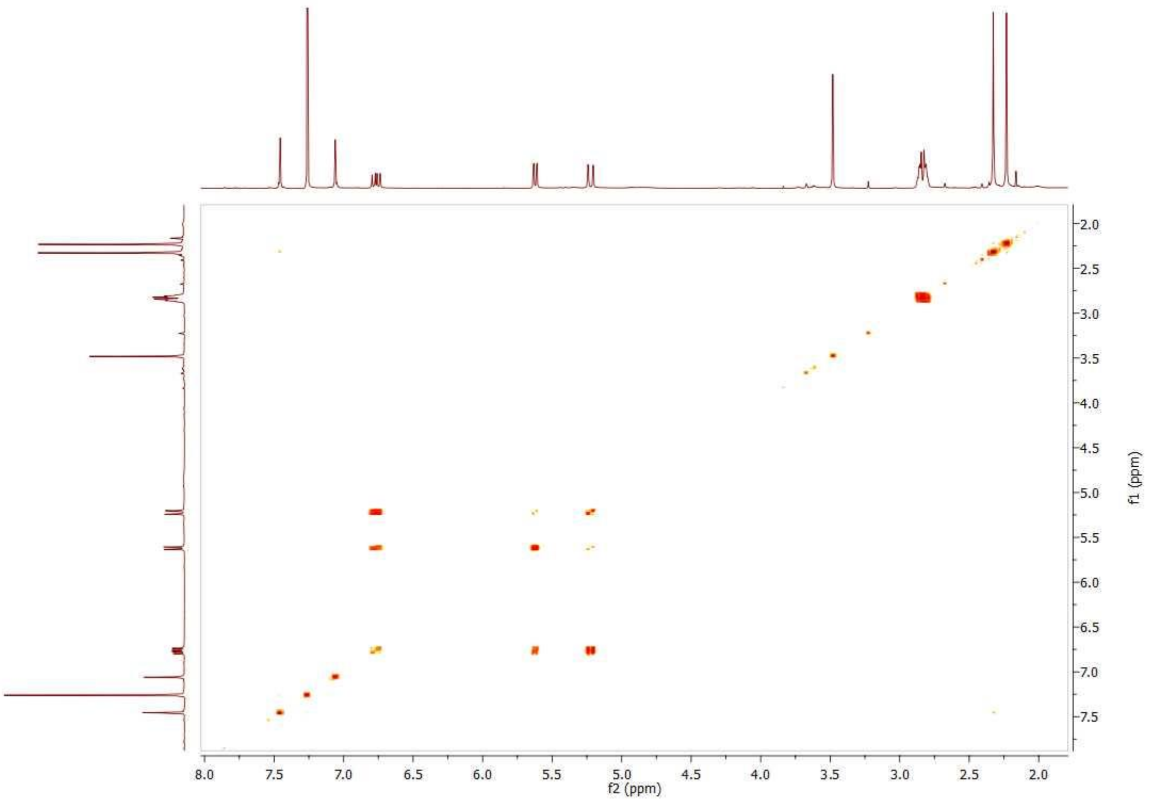

Figure S12. ^1H-^1H COSY spectrum of compound **2** (in $CDCl_3$).

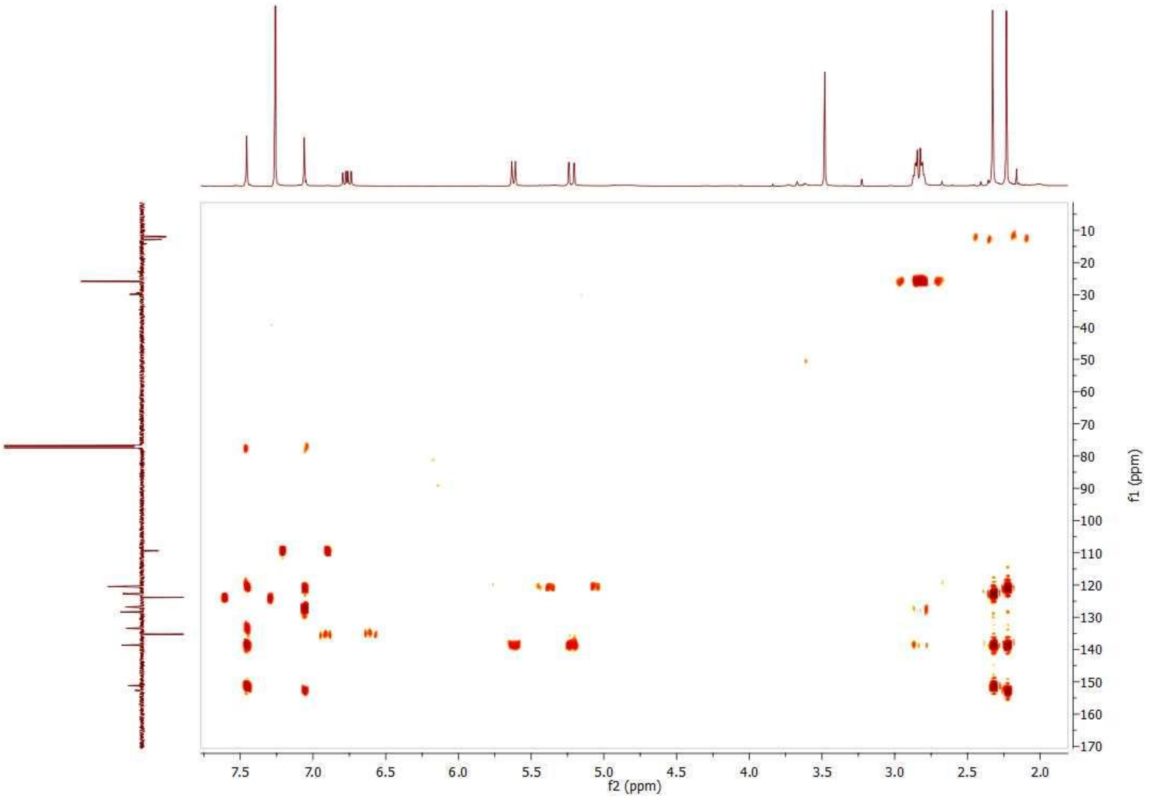

Figure S13. HMBC spectrum of compound **2** (in $CDCl_3$).

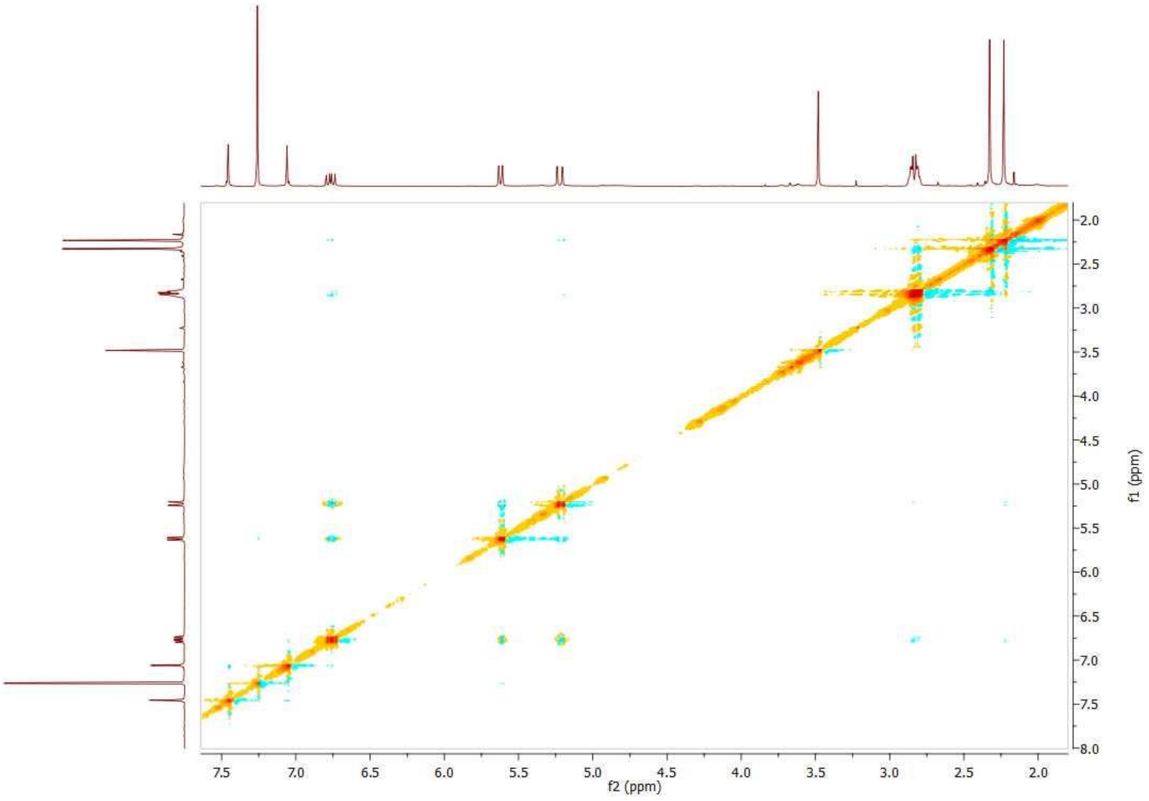

Figure S14. NOESY spectrum of compound **2** (in $CDCl_3$).

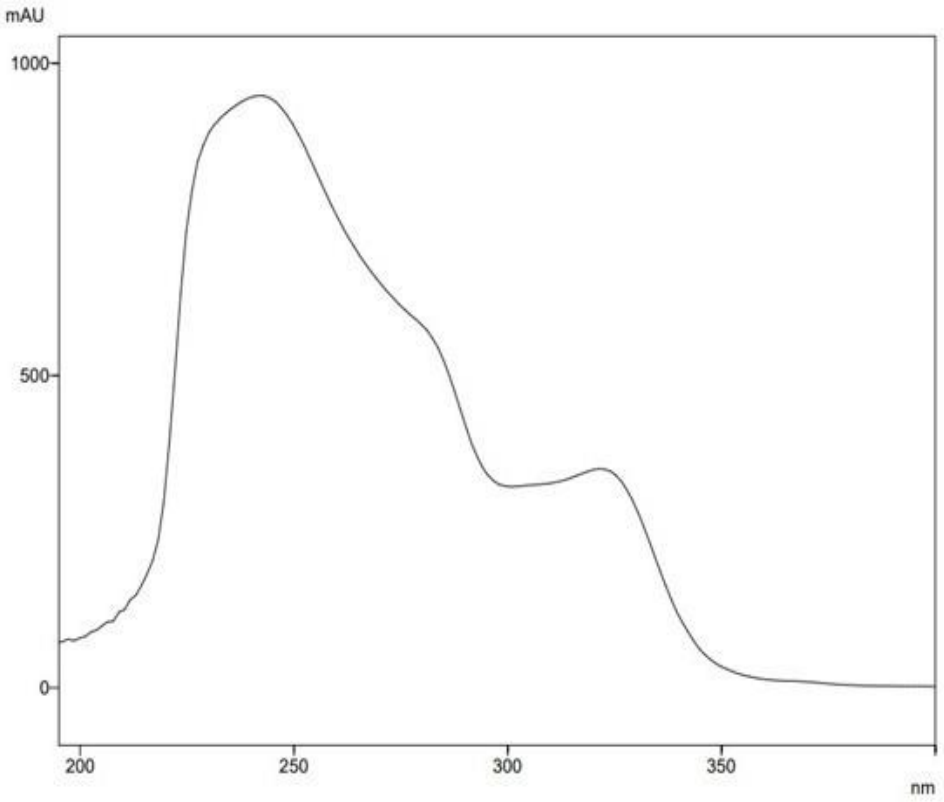

Figure S15. UV spectrum of compound **2** in MeOH.

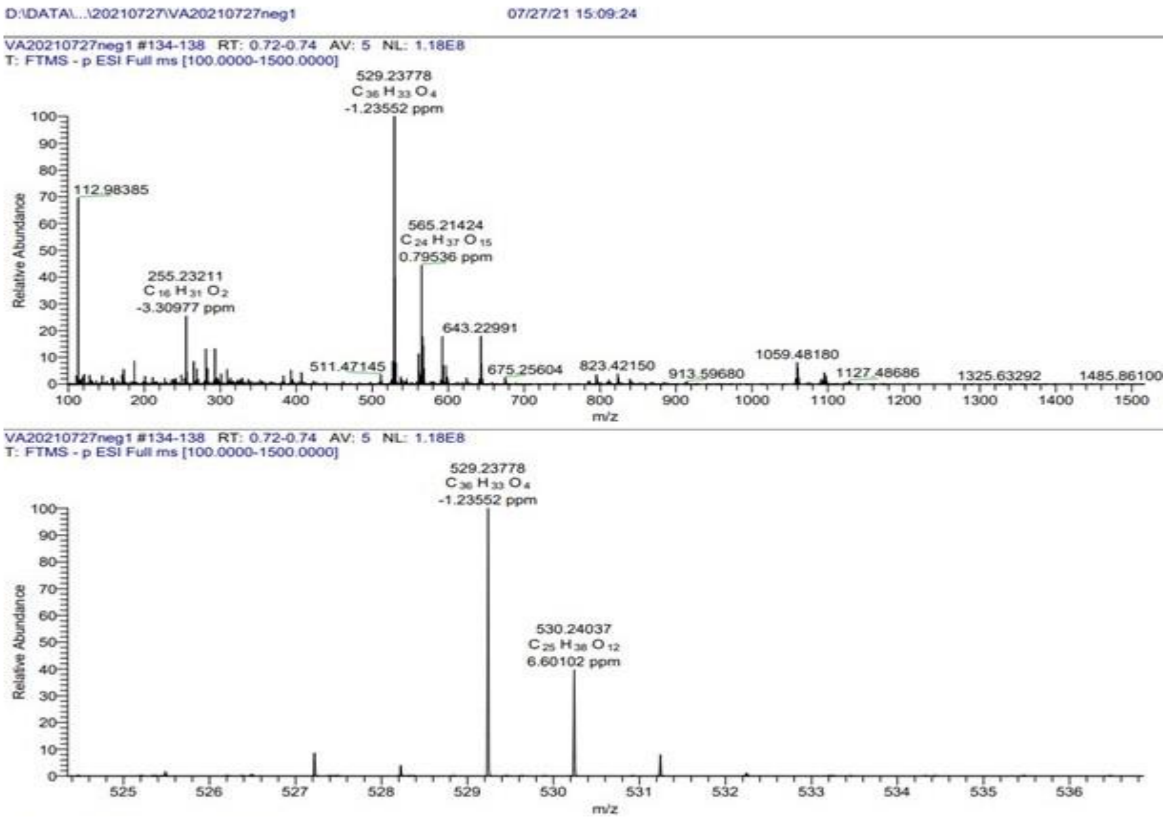

Figure S16. (−)-HRESIMS spectrum of compound **2**.

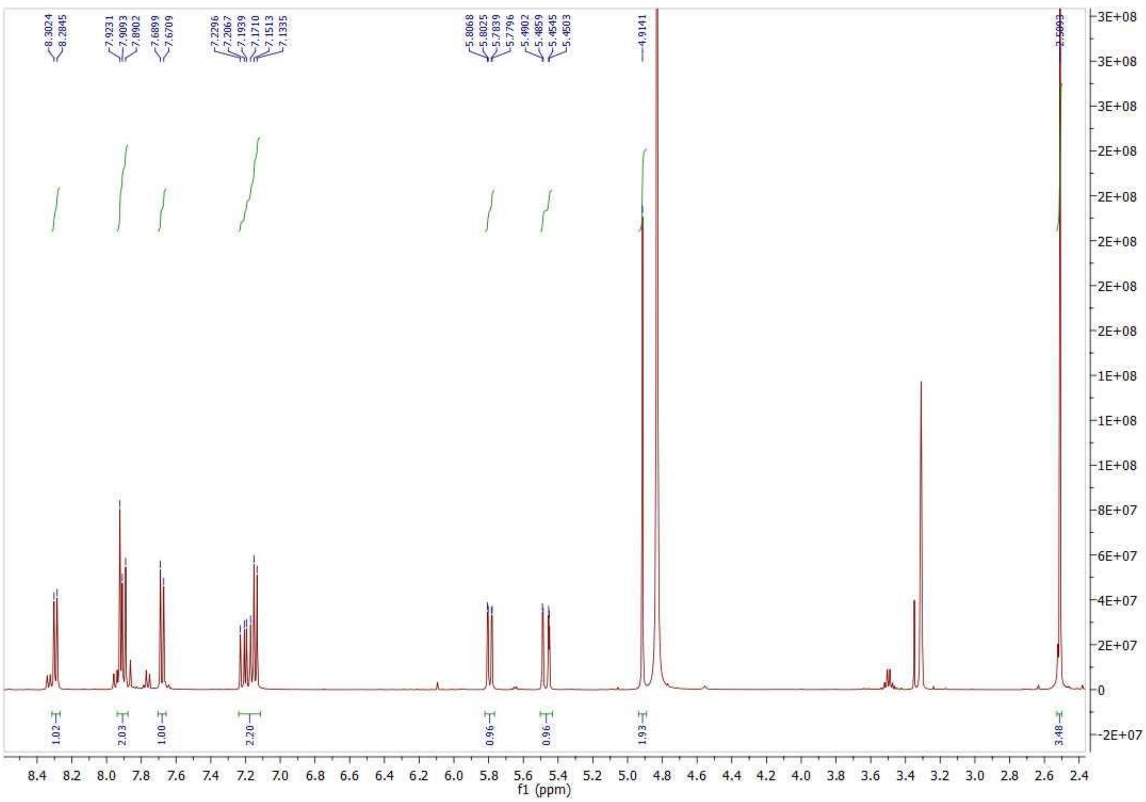

Figure S17. ^{1}H NMR spectrum of compound **3** (500 MHz, in CD_3OD).

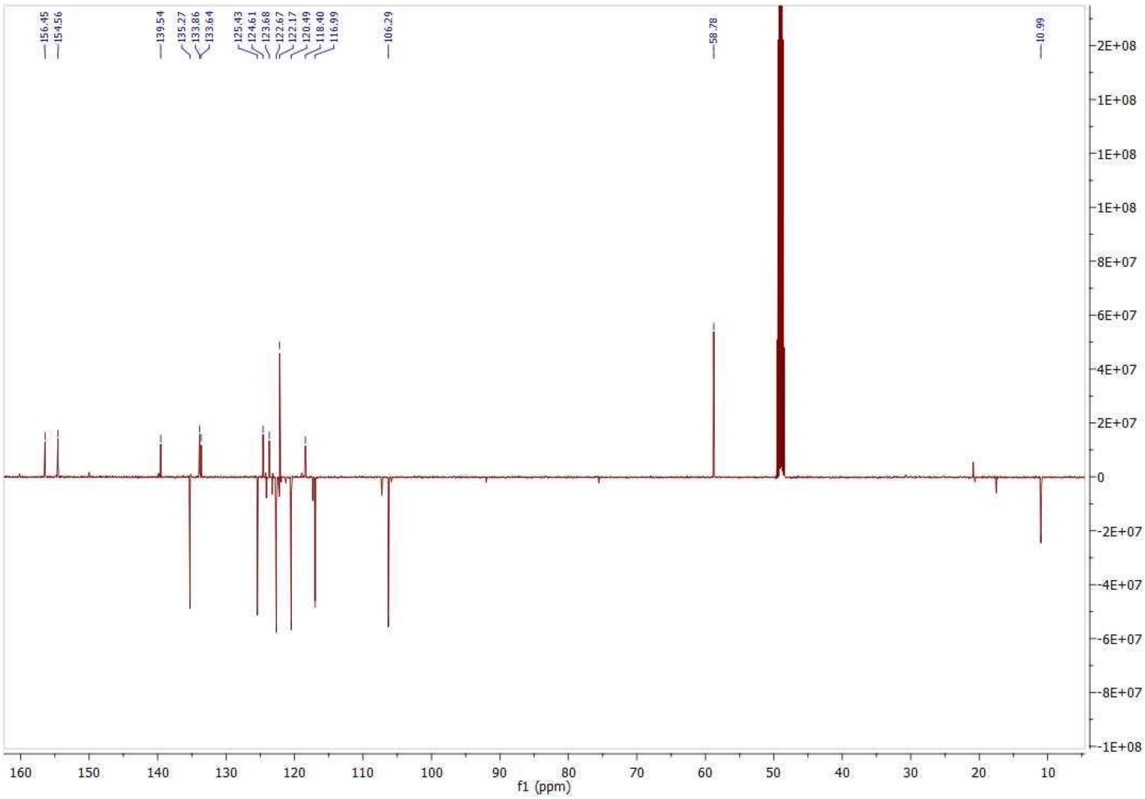

Figure S18. ^{13}C (JMOD) NMR spectrum of compound **3** (125 MHz, in CD_3OD).

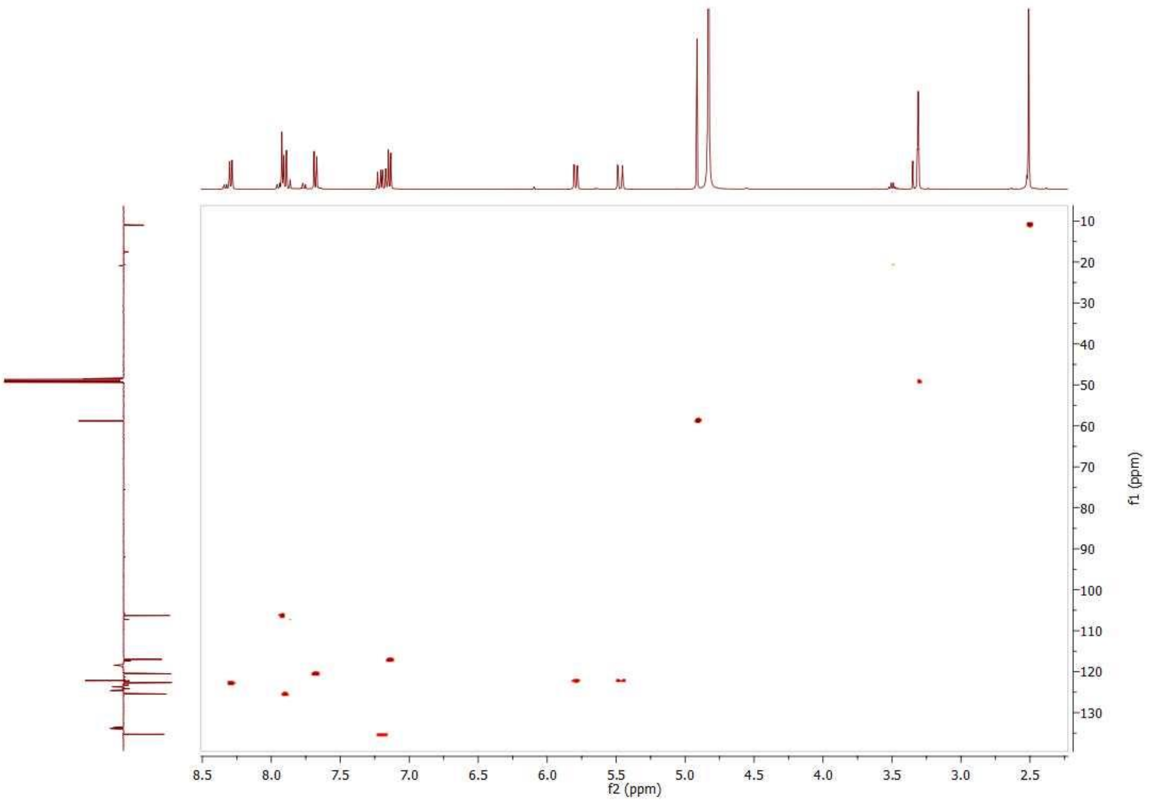

Figure S19. HSQC spectrum of compound **3** (in CD_3OD).

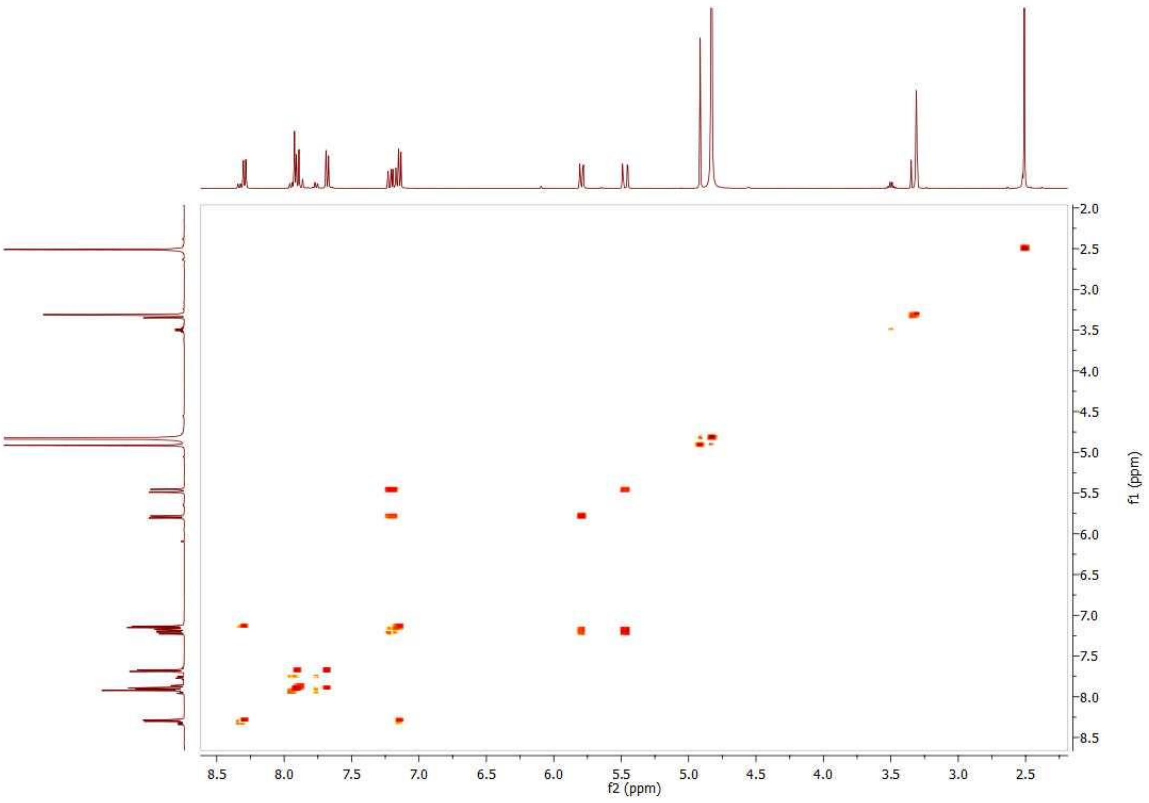

Figure S20. ^1H-^1H COSY spectrum of compound **3** (in CD_3OD).

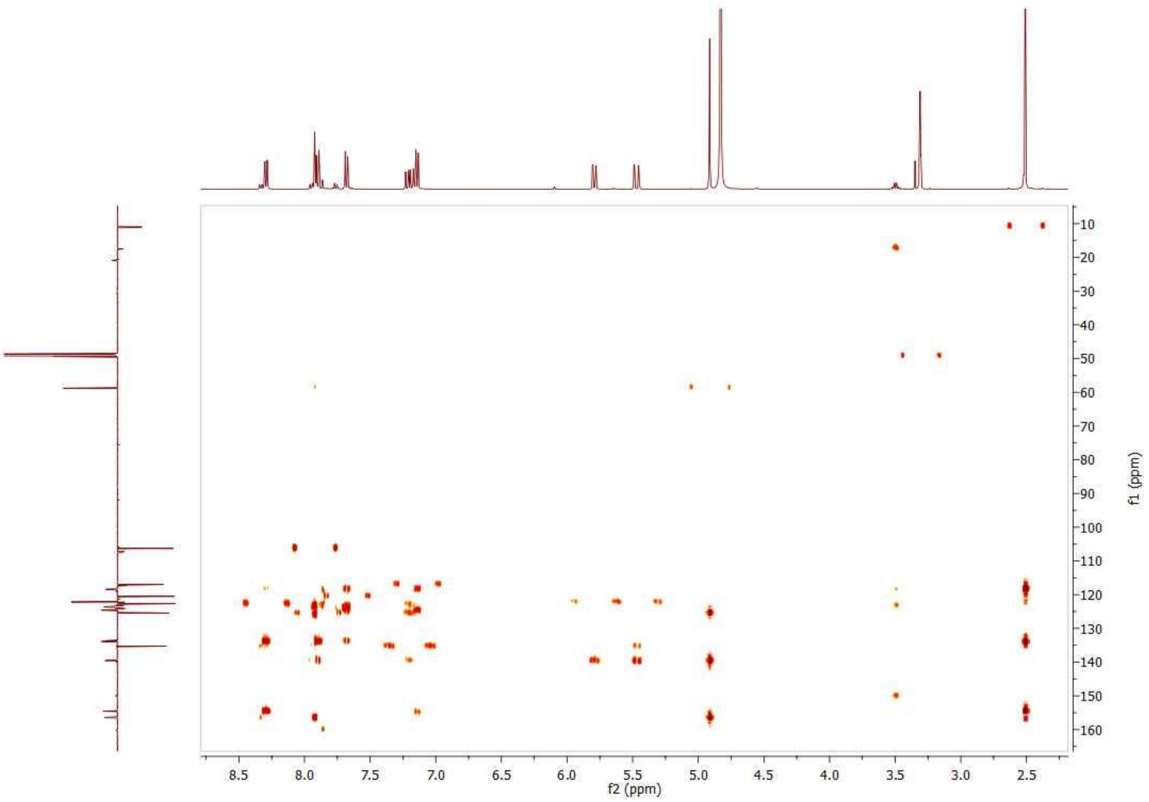

Figure S21. HMBC spectrum of compound **3** (in CD_3OD).

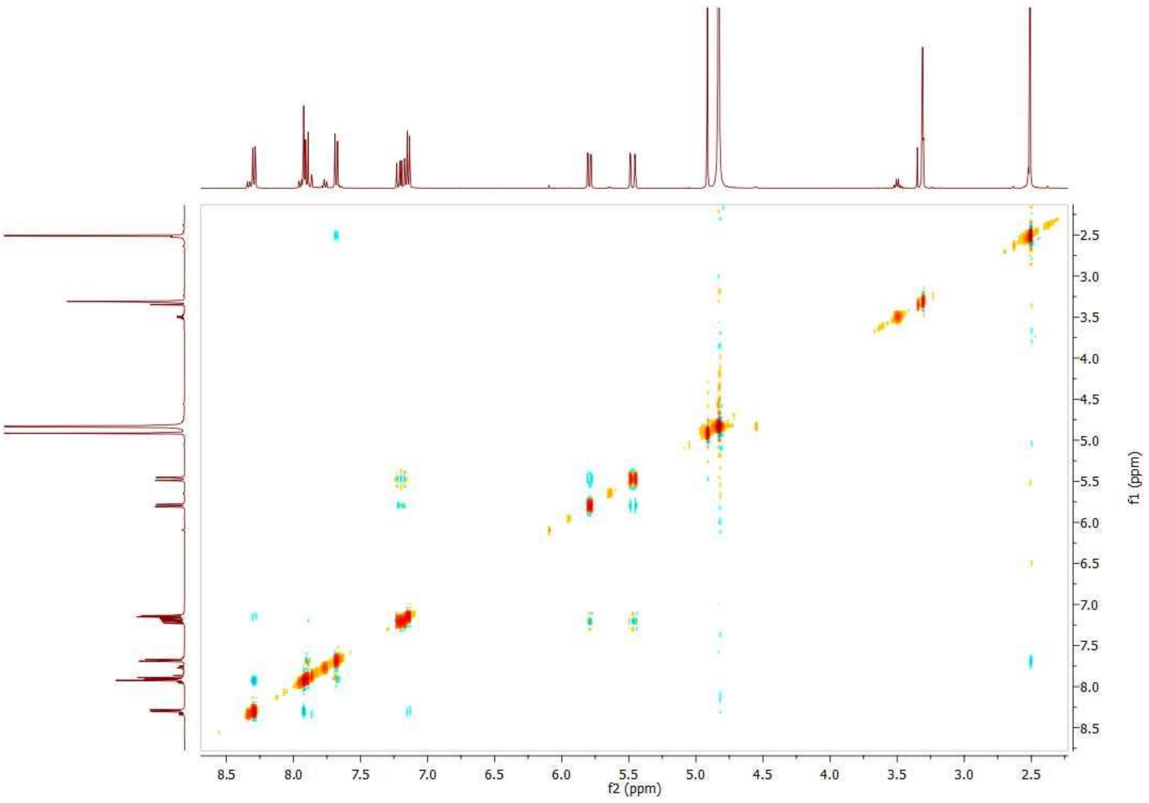

Figure S22. NOESY spectrum of compound **3** (in CD_3OD).

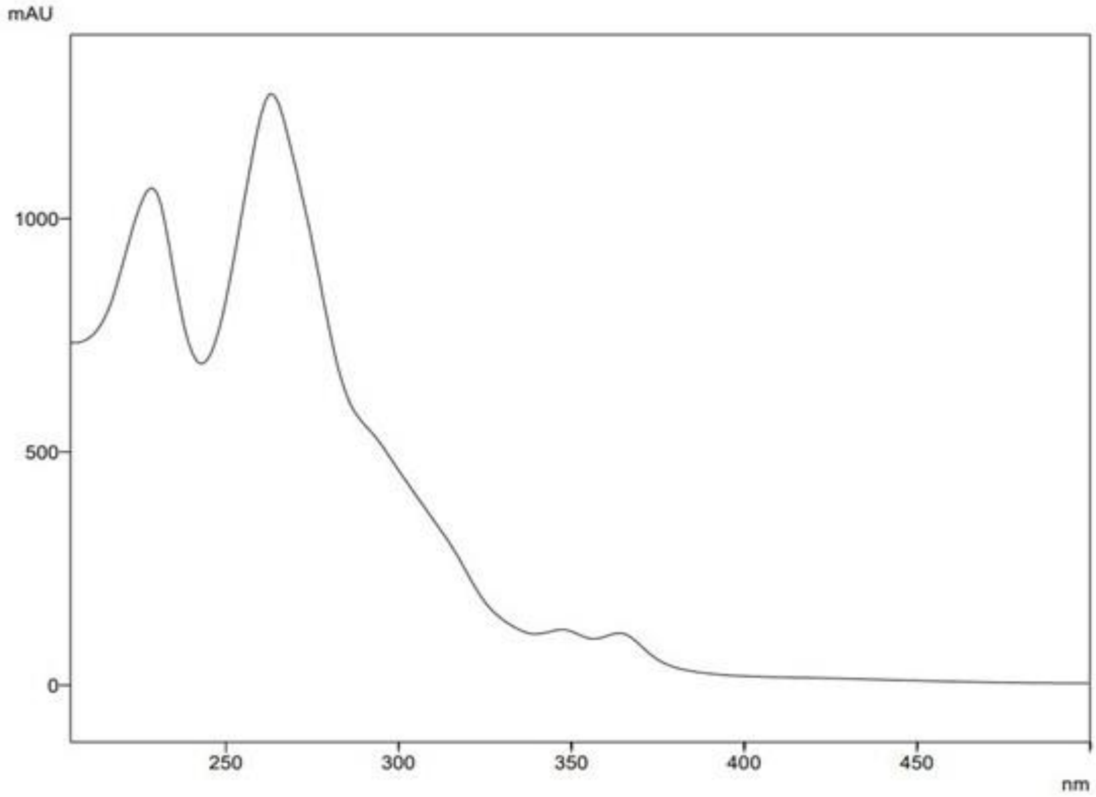

Figure S23. UV spectrum of compound **3** in MeOH.

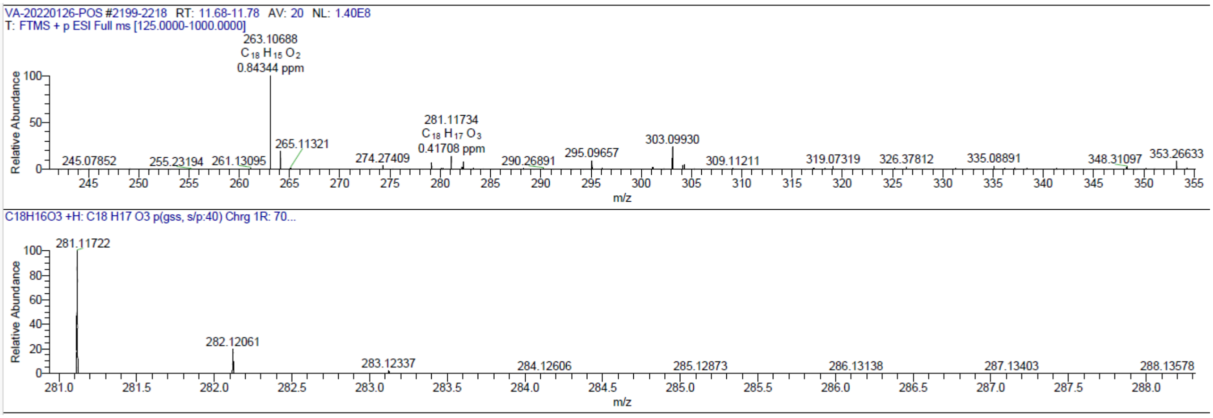

Figure S24. (+)-HRESIMS spectrum of compound **3**.

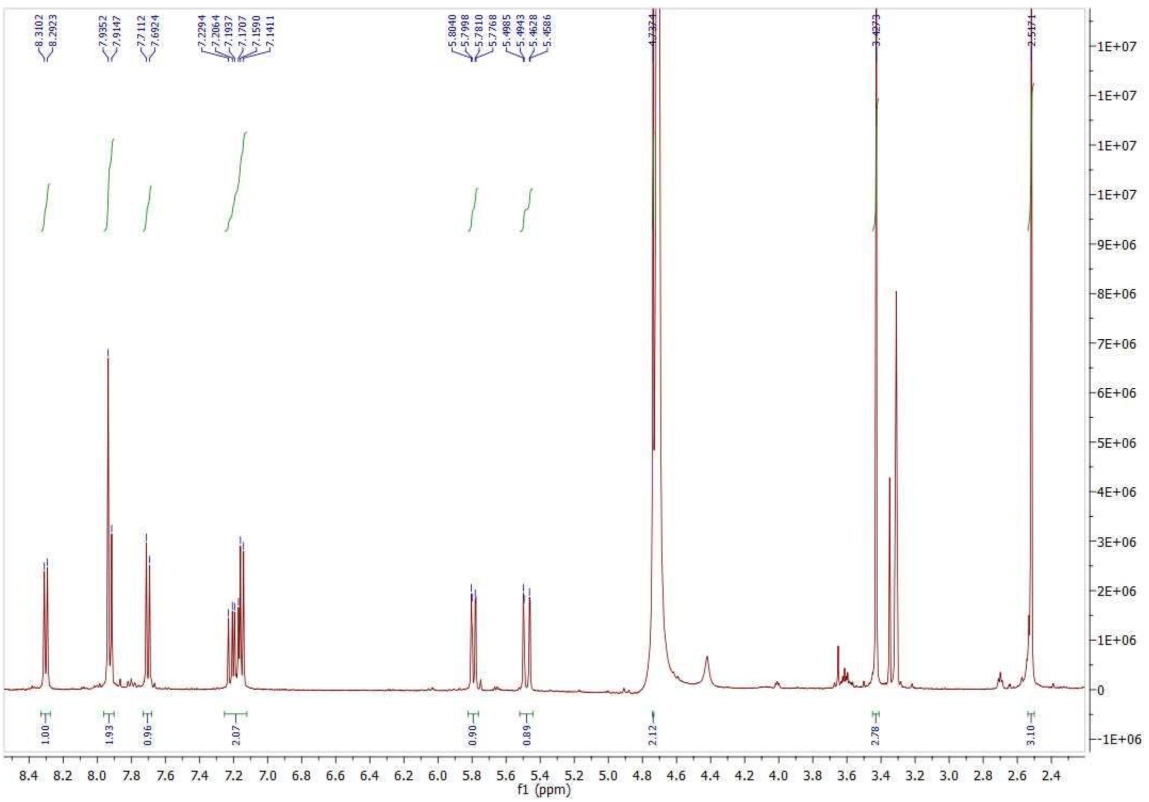

Figure S25. ^{1}H NMR spectrum of compound **4** (500 MHz, in CD_3OD).

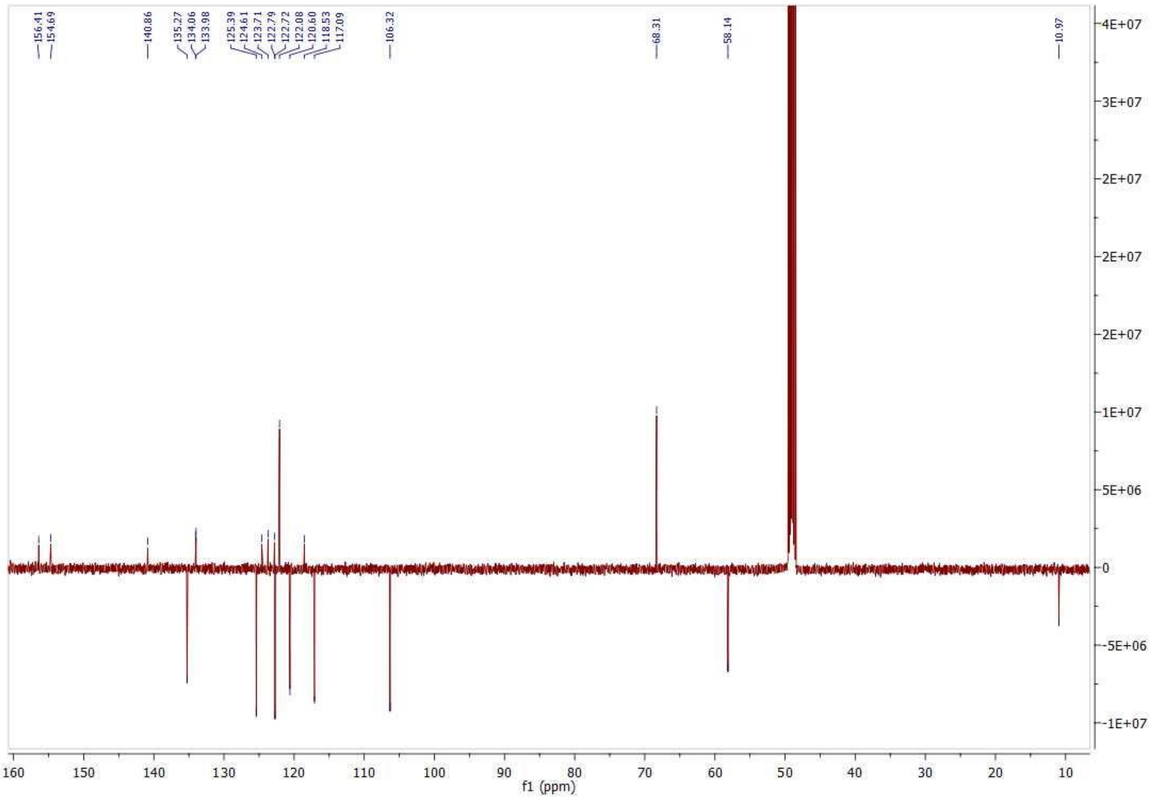

Figure S26. ^{13}C (JMOD) NMR spectrum of compound **4** (125 MHz, in CD_3OD).

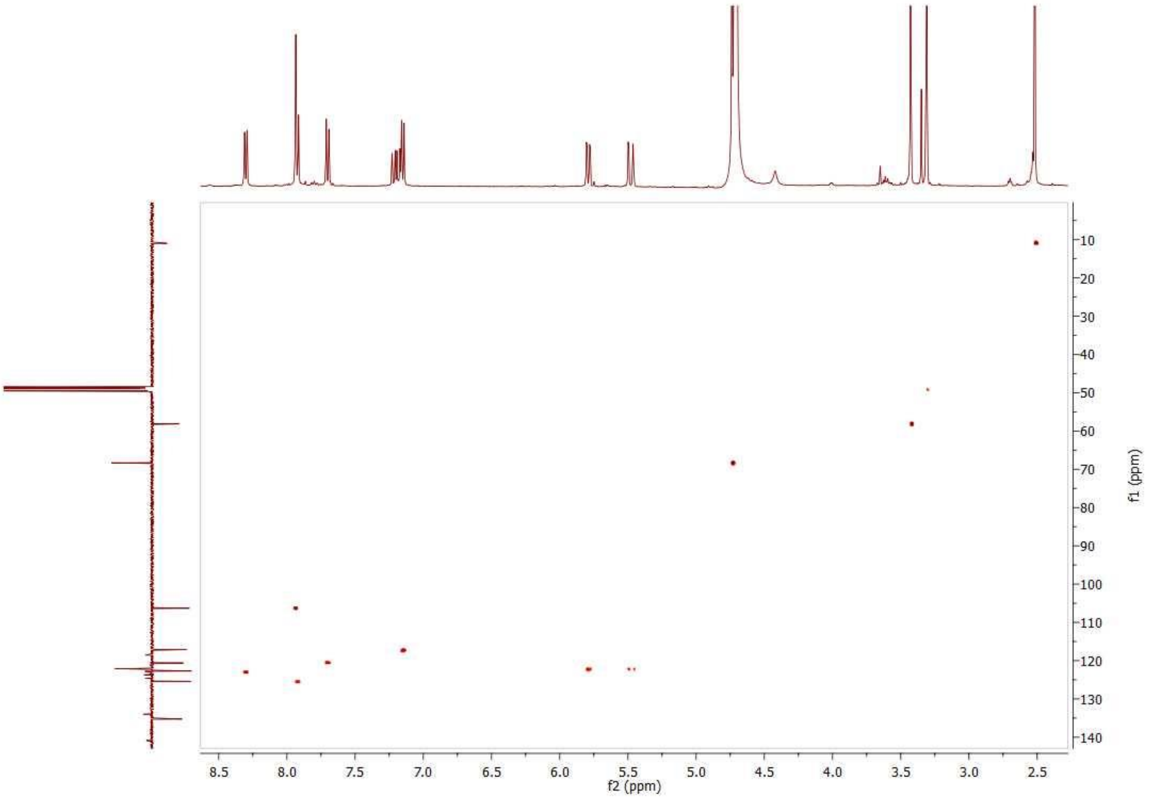

Figure S27. HSQC spectrum of compound **4** (in CD_3OD).

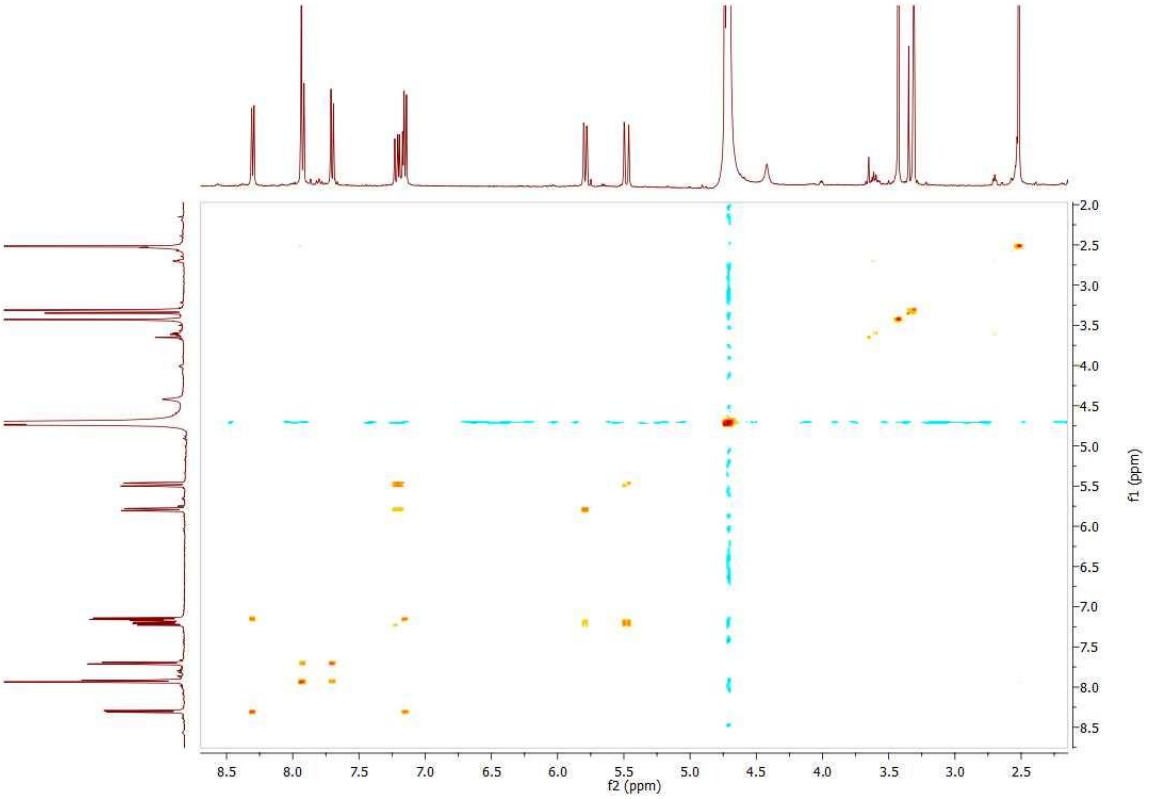

Figure S28. ^1H-^1H COSY spectrum of compound **4** (in CD_3OD).

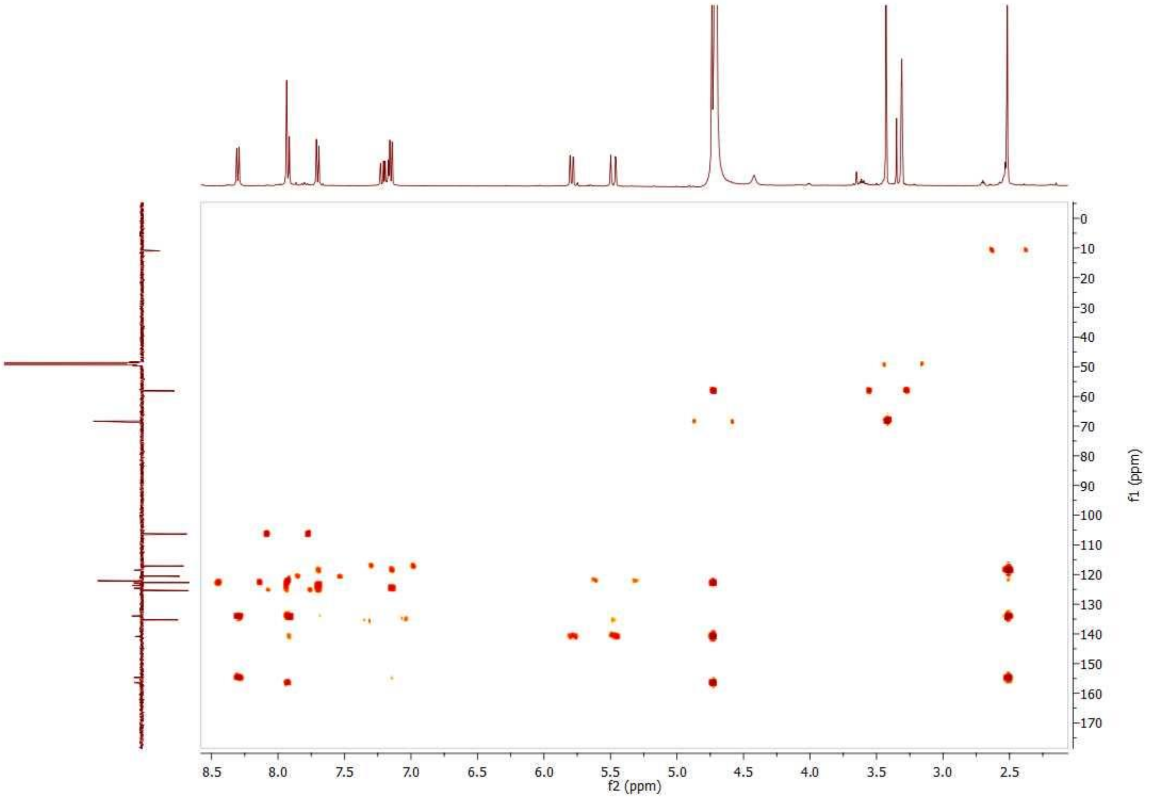

Figure S29. HMBC spectrum of compound **4** (in CD_3OD).

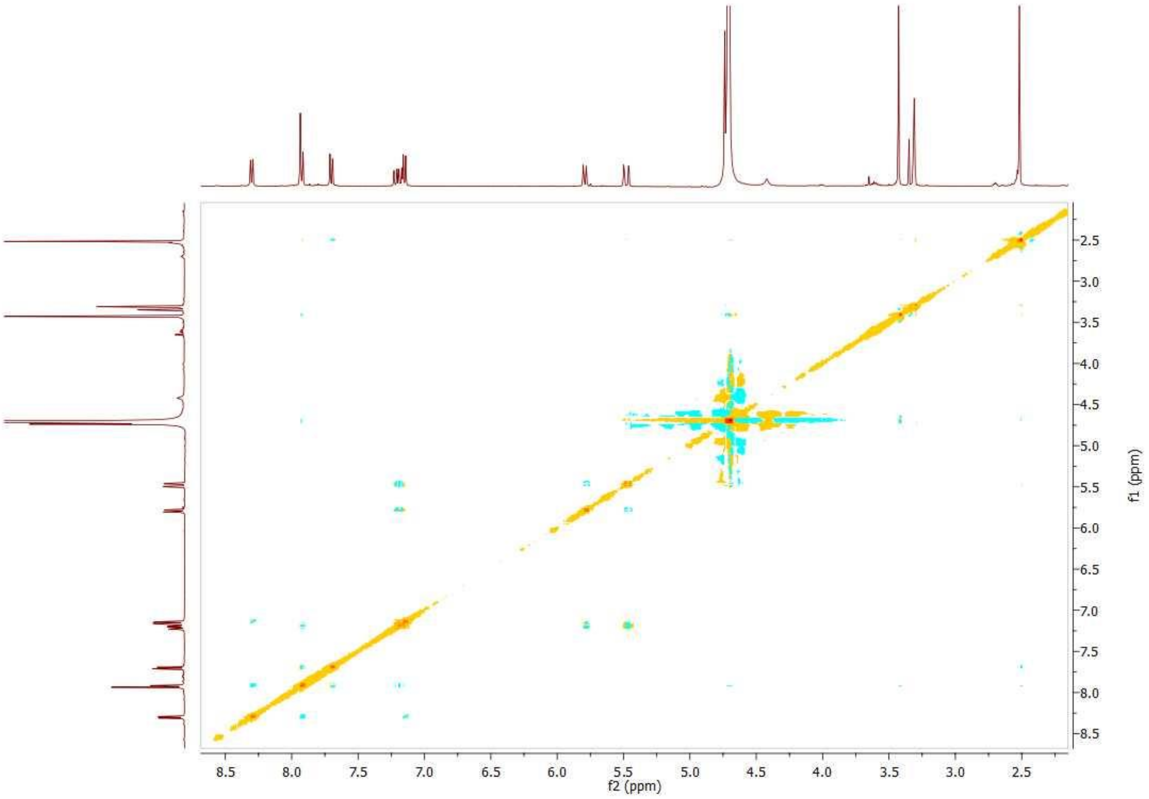

Figure S30. NOESY spectrum of compound **4** (in CD_3OD).

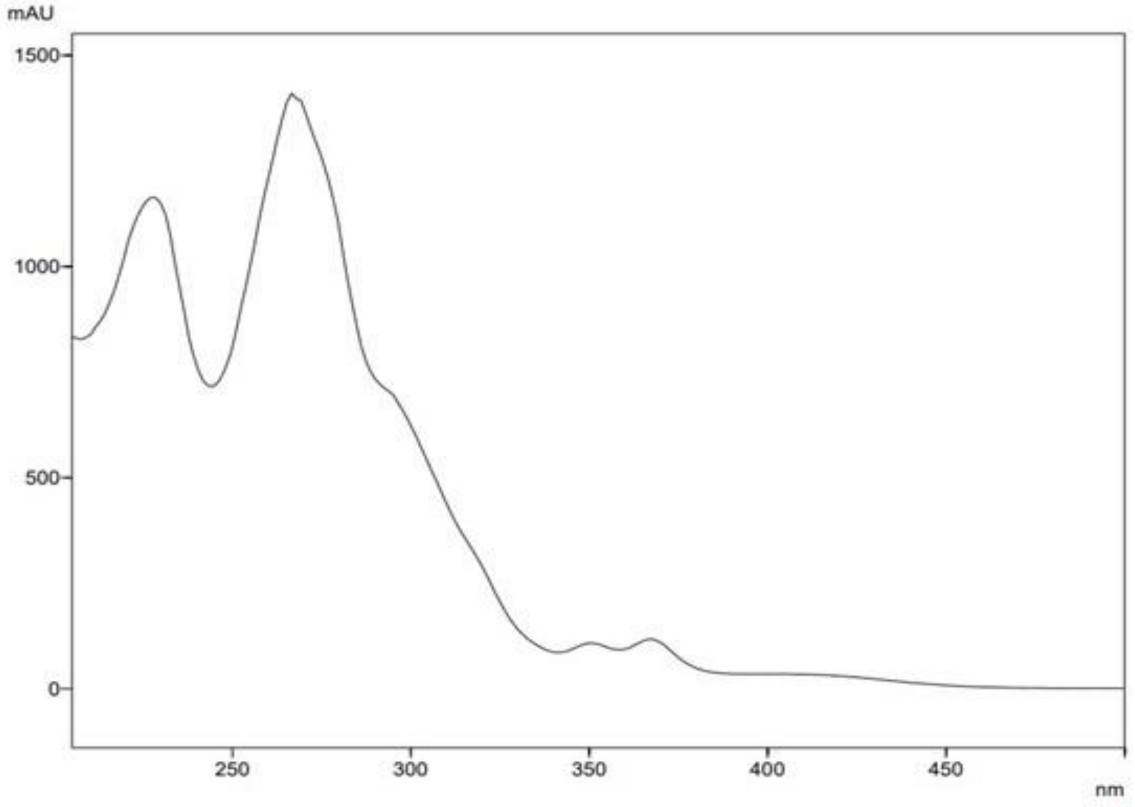

Figure S31. UV spectrum of compound **4** in MeOH.

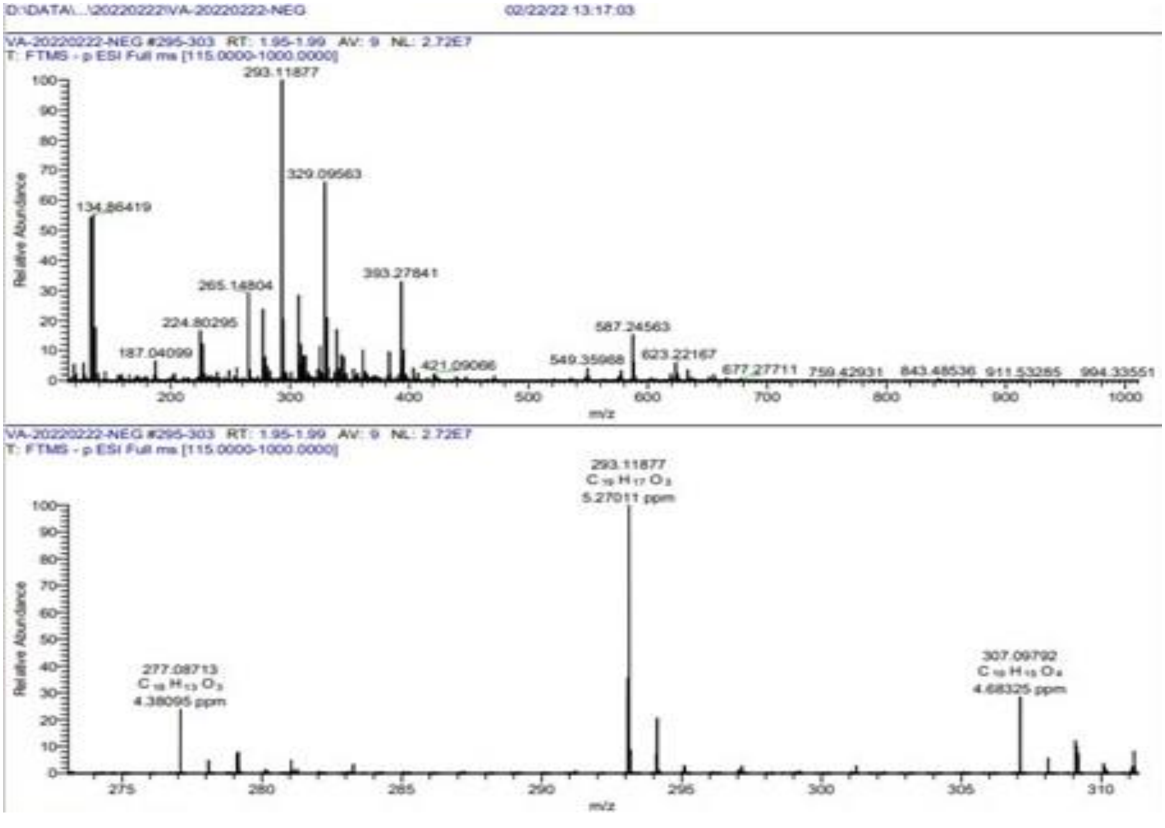

Figure S32. (−)-HRESIMS spectrum of compound **4**.

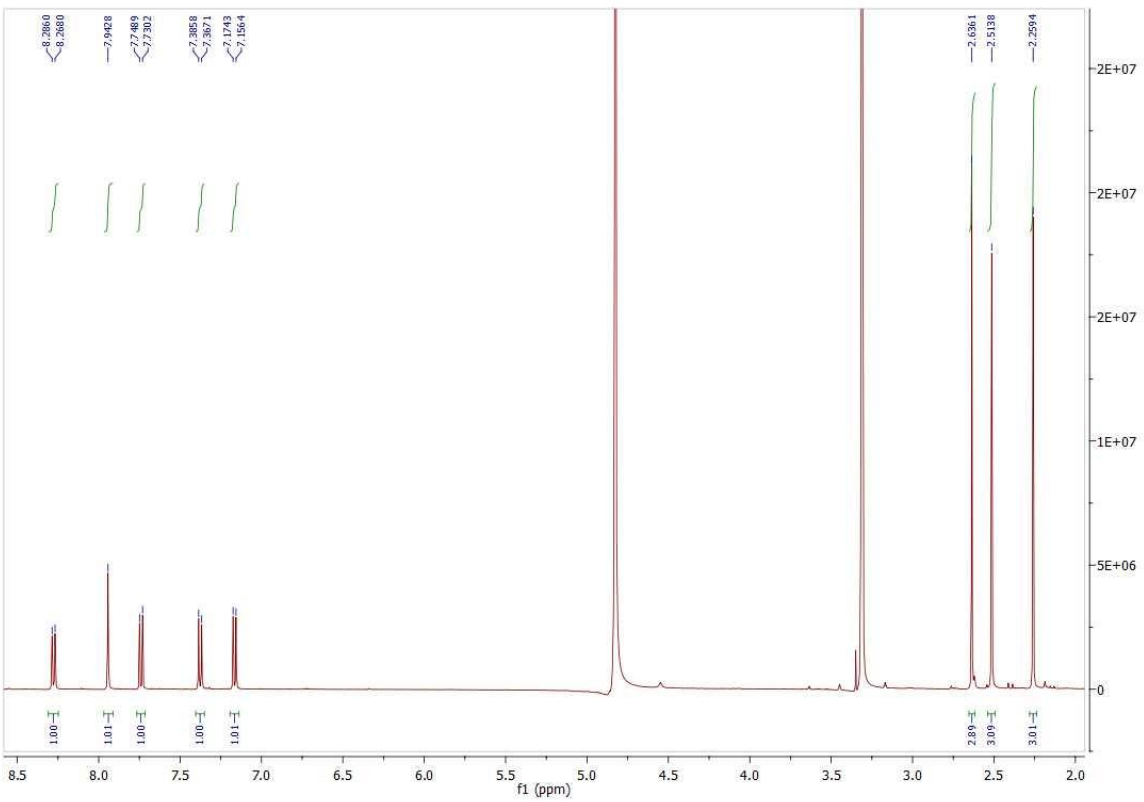

Figure S33. ^{1}H NMR spectrum of compound **5** (500 MHz, in CD_3OD).

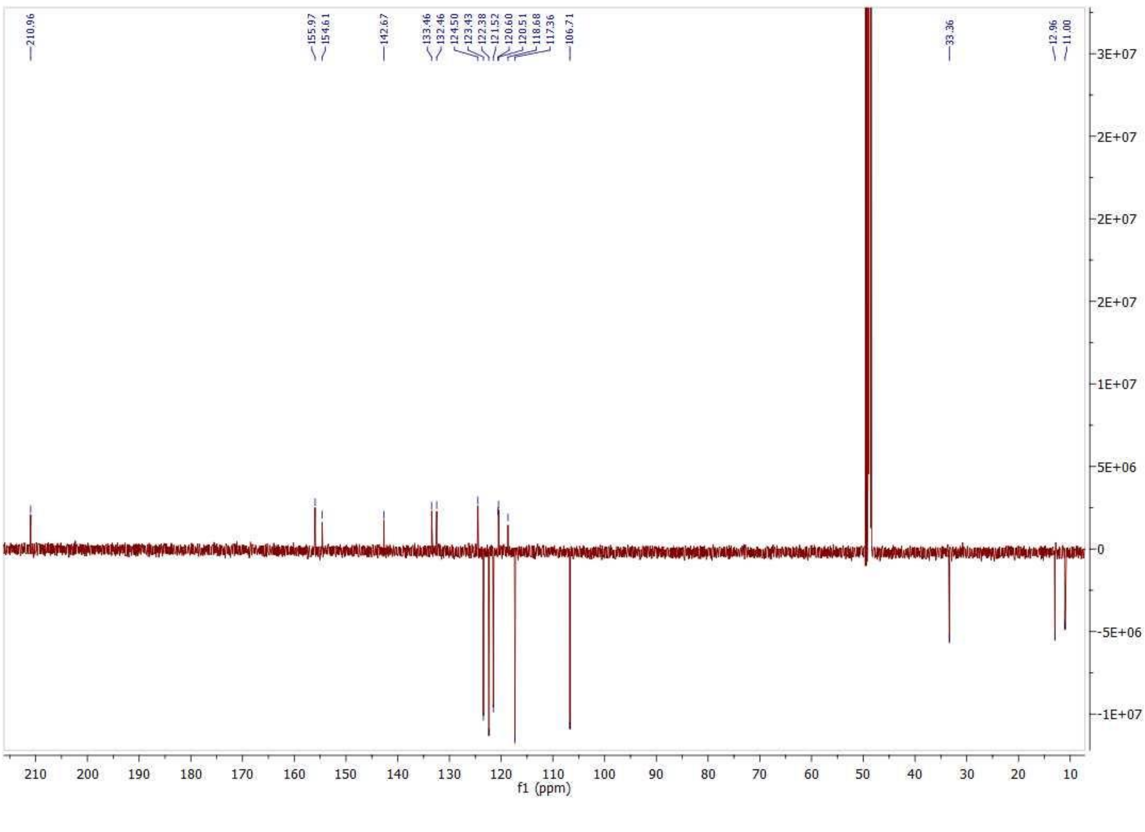

Figure S34. ^{13}C (JMOD) NMR spectrum of compound **5** (125 MHz, in CD_3OD).

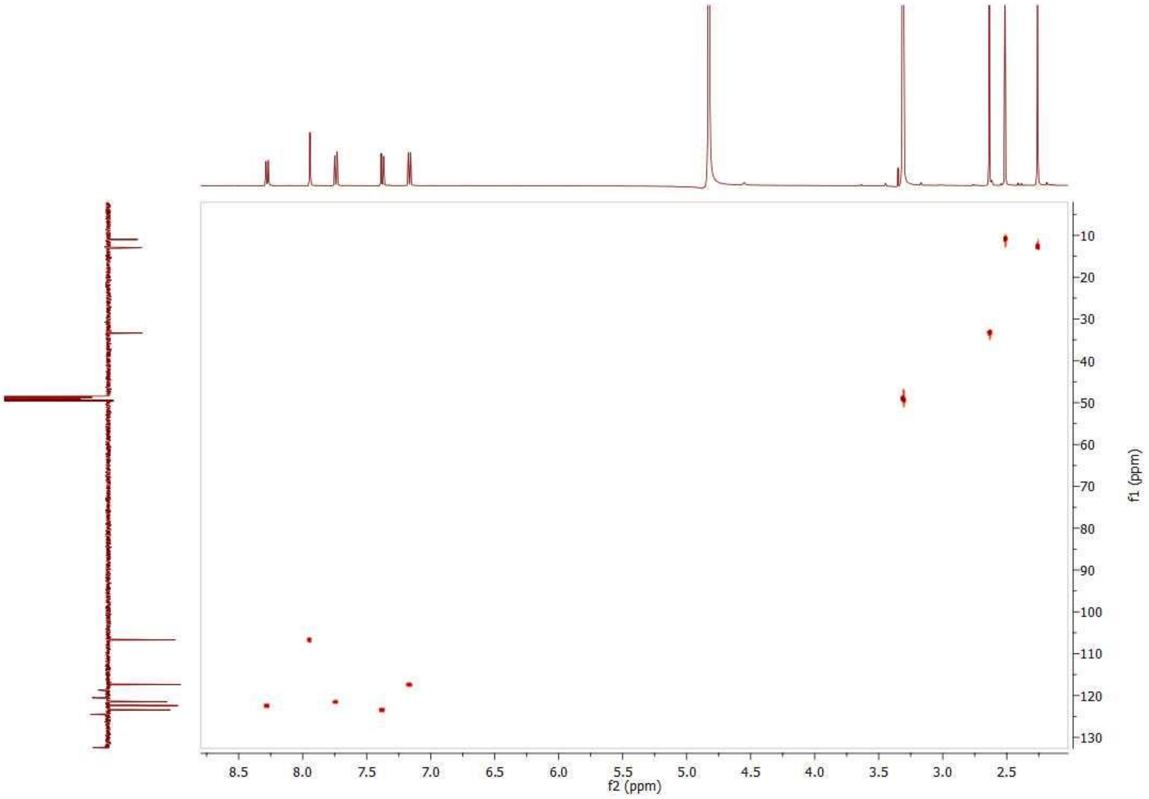

Figure S35. HSQC spectrum of compound **5** (in CD_3OD).

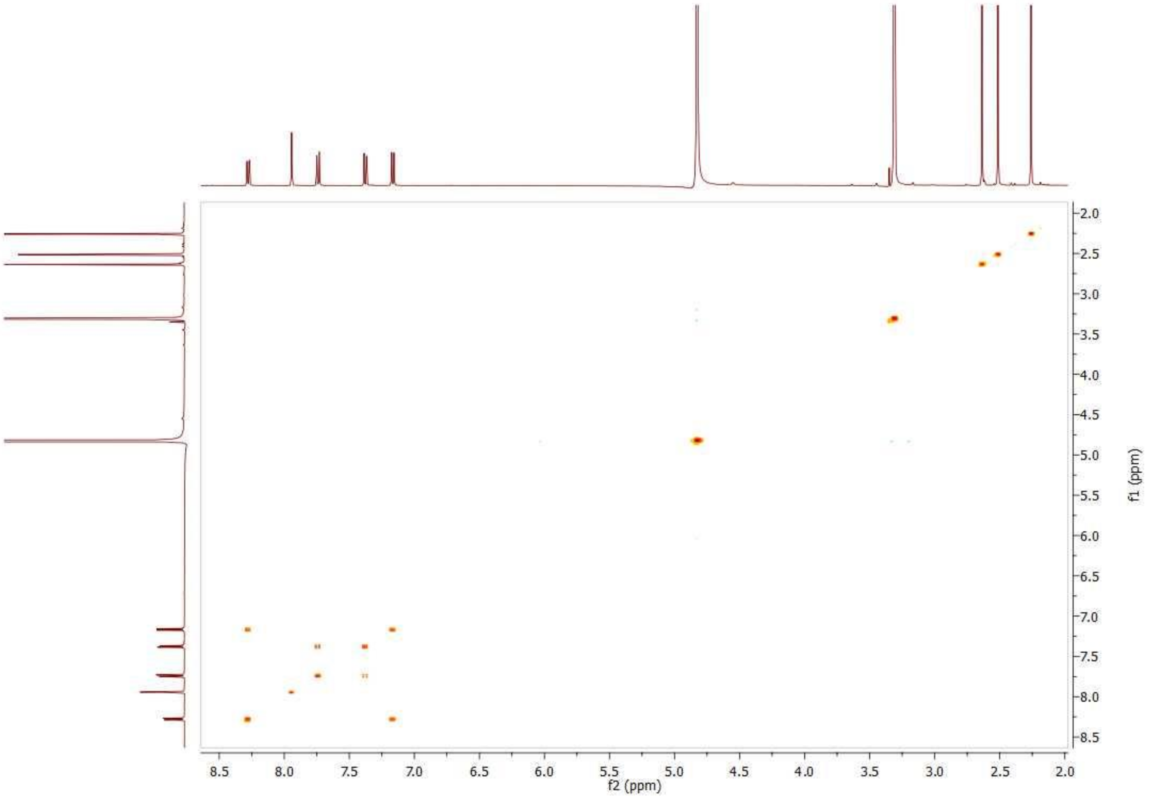

Figure S36. ^1H-^1H COSY spectrum of compound **5** (in CD_3OD).

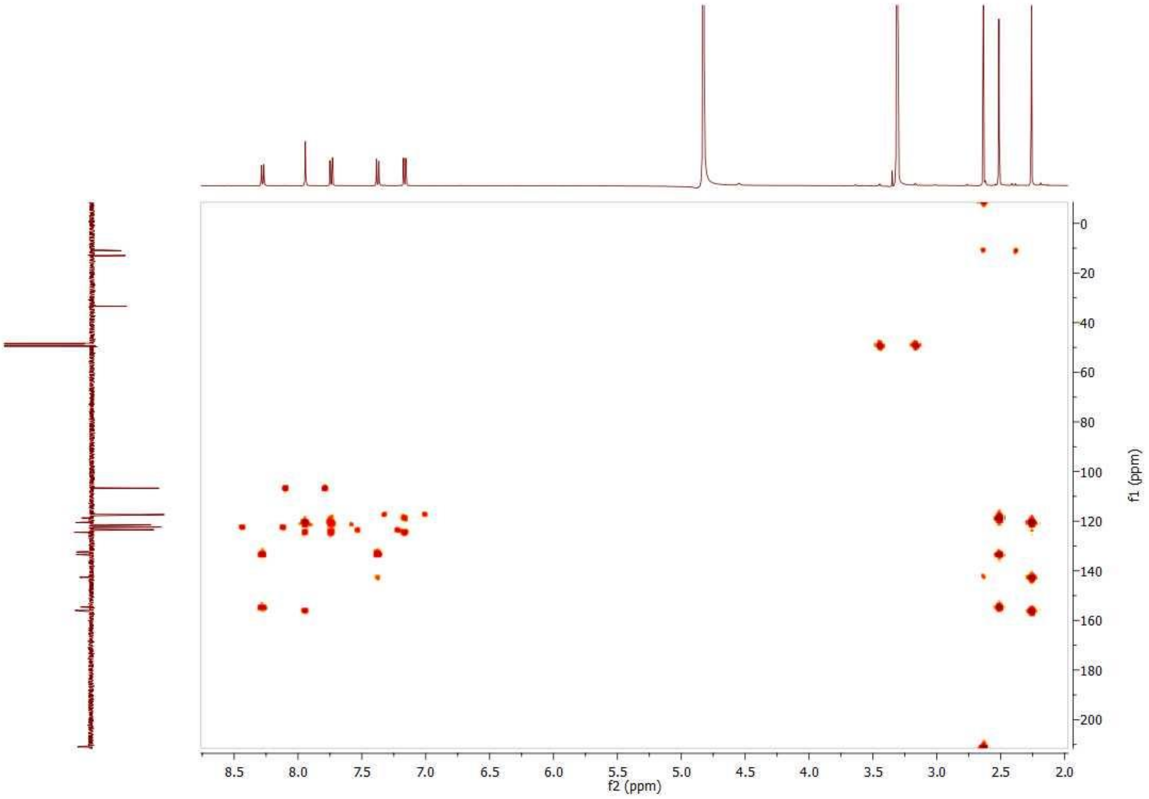

Figure S37. HMBC spectrum of compound **5** (in CD_3OD).

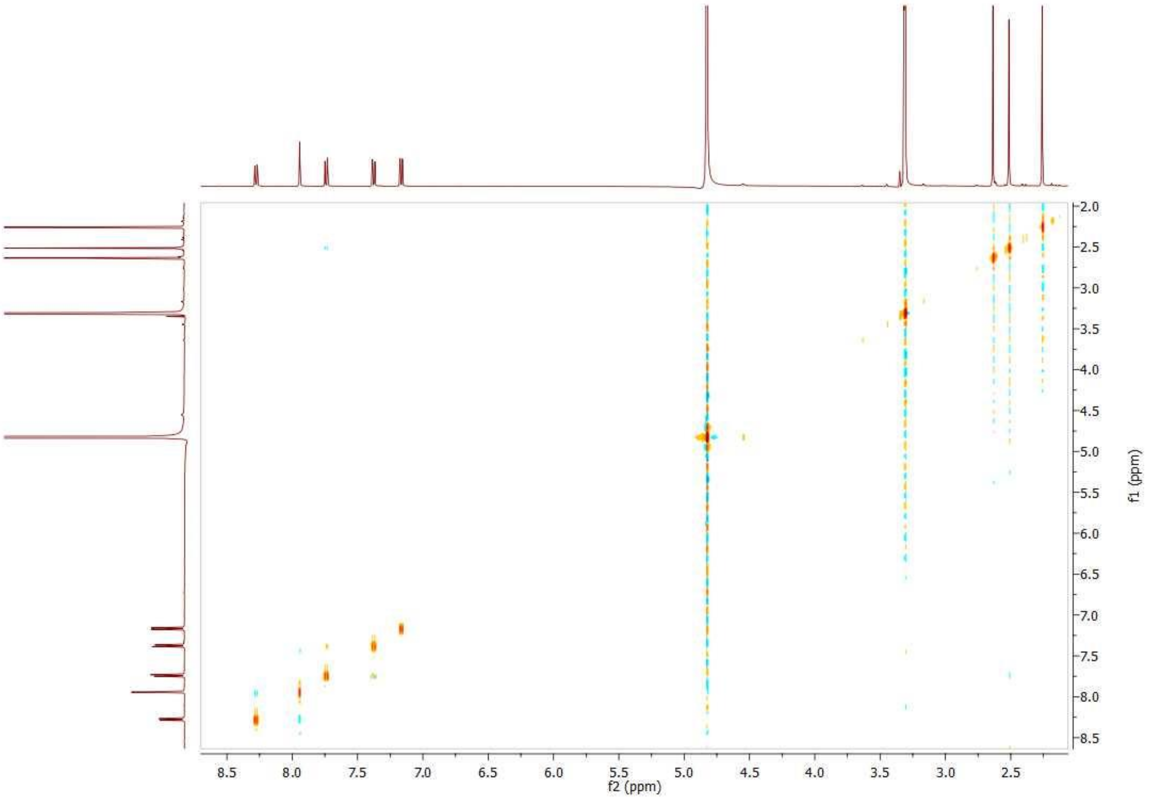

Figure S38. NOESY spectrum of compound **5** (in CD_3OD).

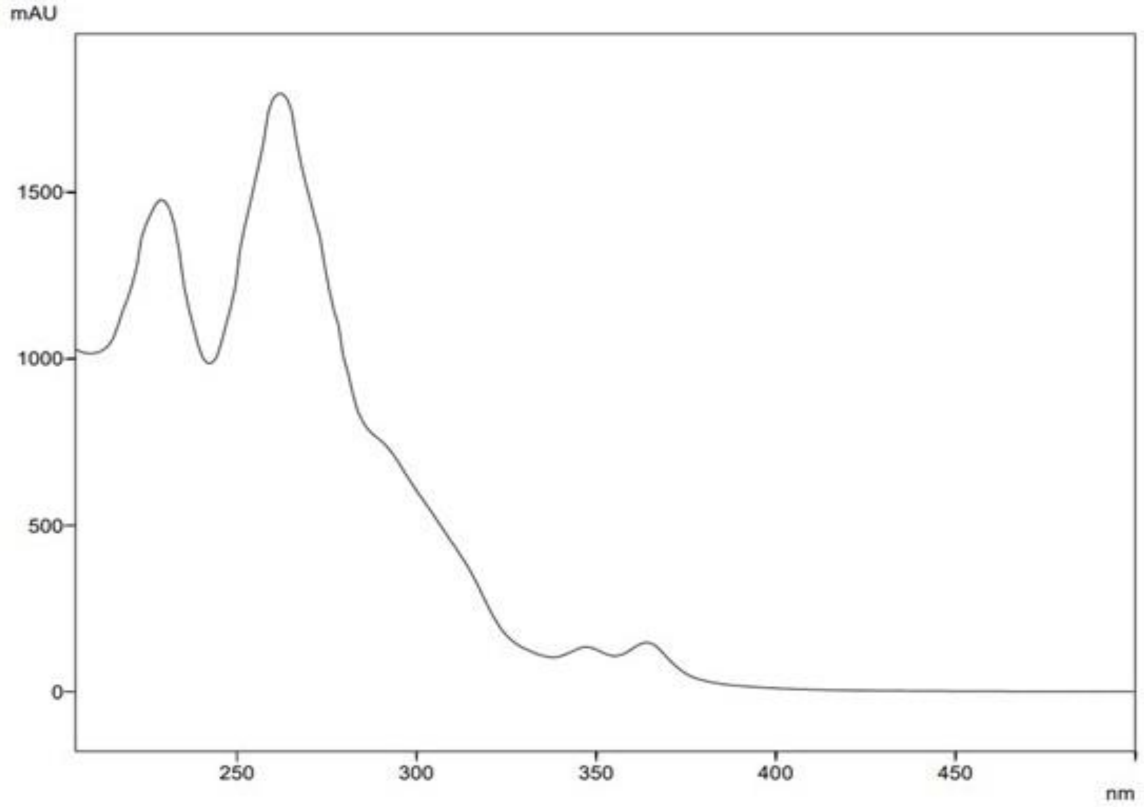

Figure S39. UV spectrum of compound **5** in MeOH.

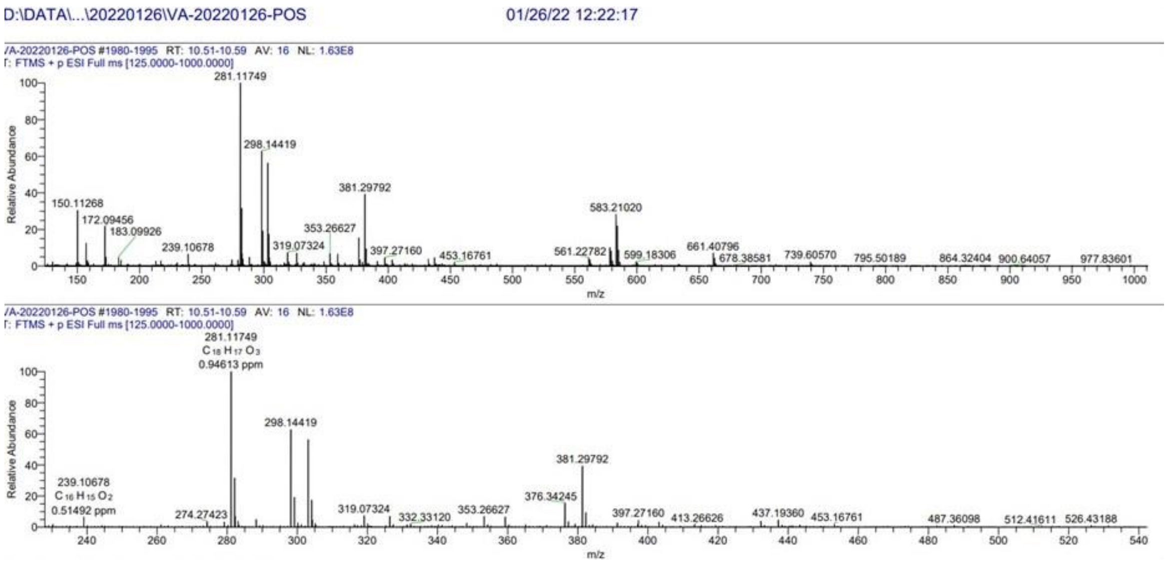

Figure S40. (+)-HRESIMS spectrum of compound **5**.

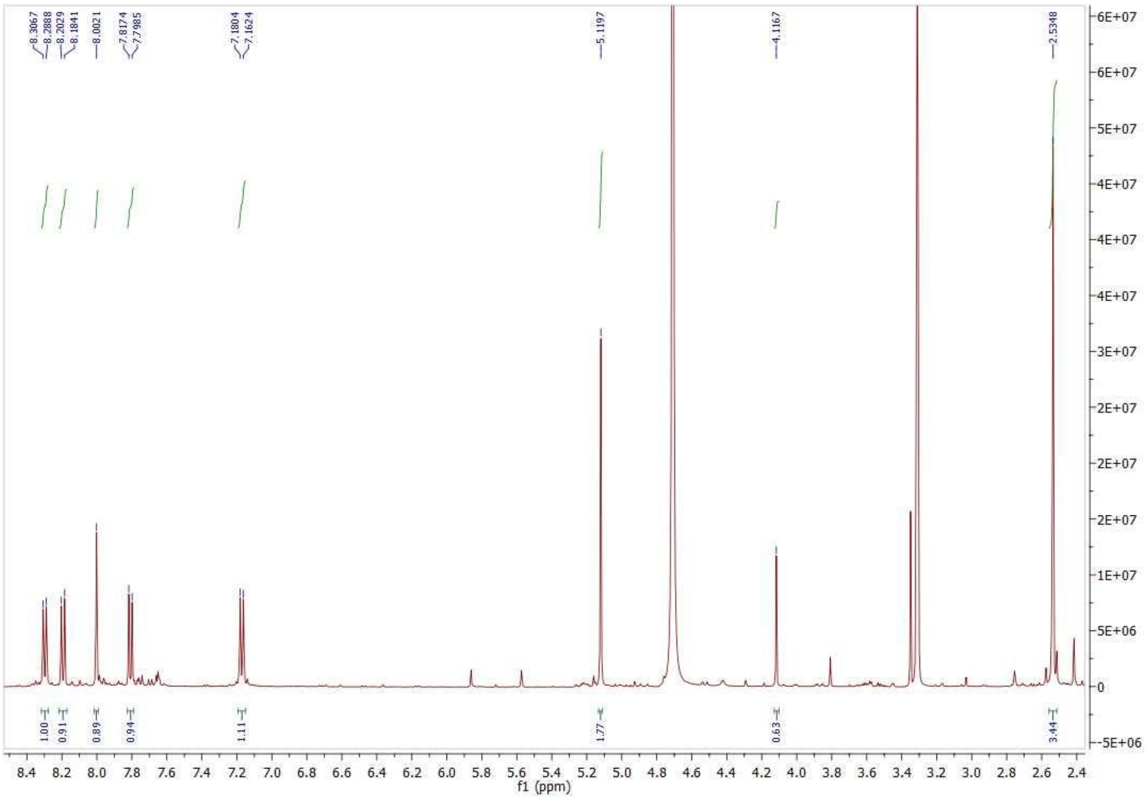

Figure S41. ^{1}H NMR spectrum of compound **6** (500 MHz, in CD_3OD).

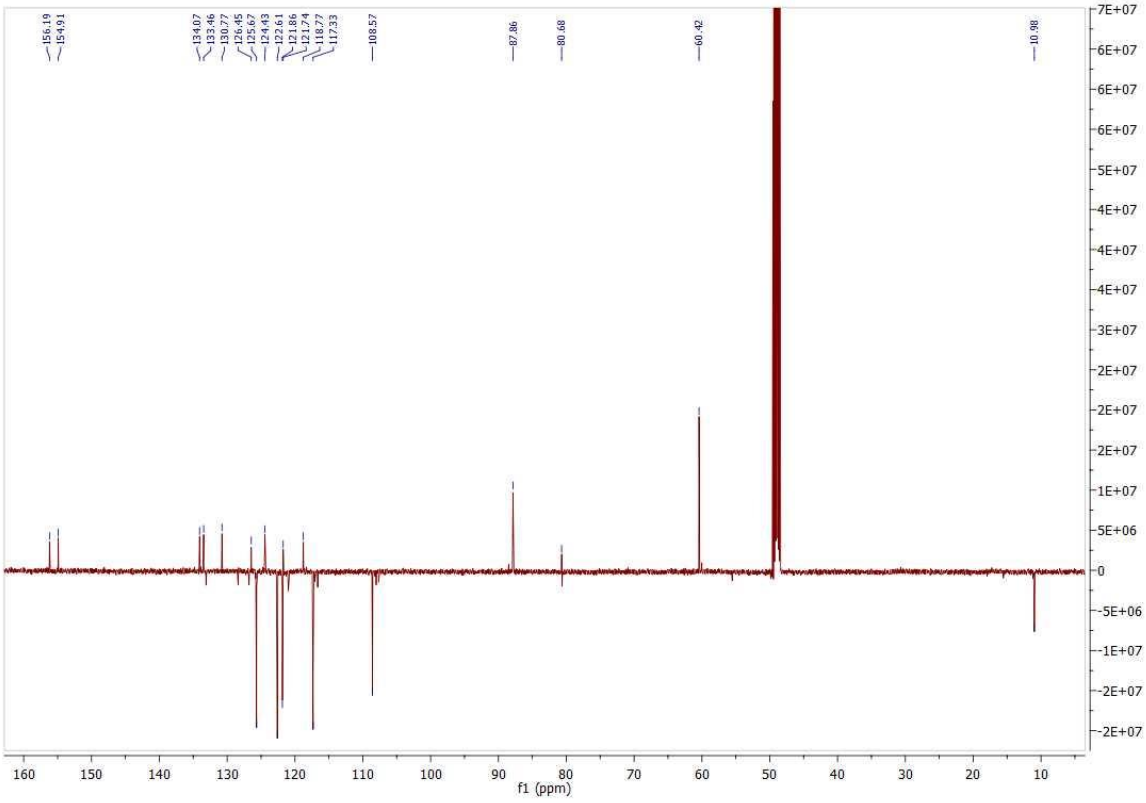

Figure S42. ^{13}C (JMOD) NMR spectrum of compound **6** (125 MHz, in CD_3OD).

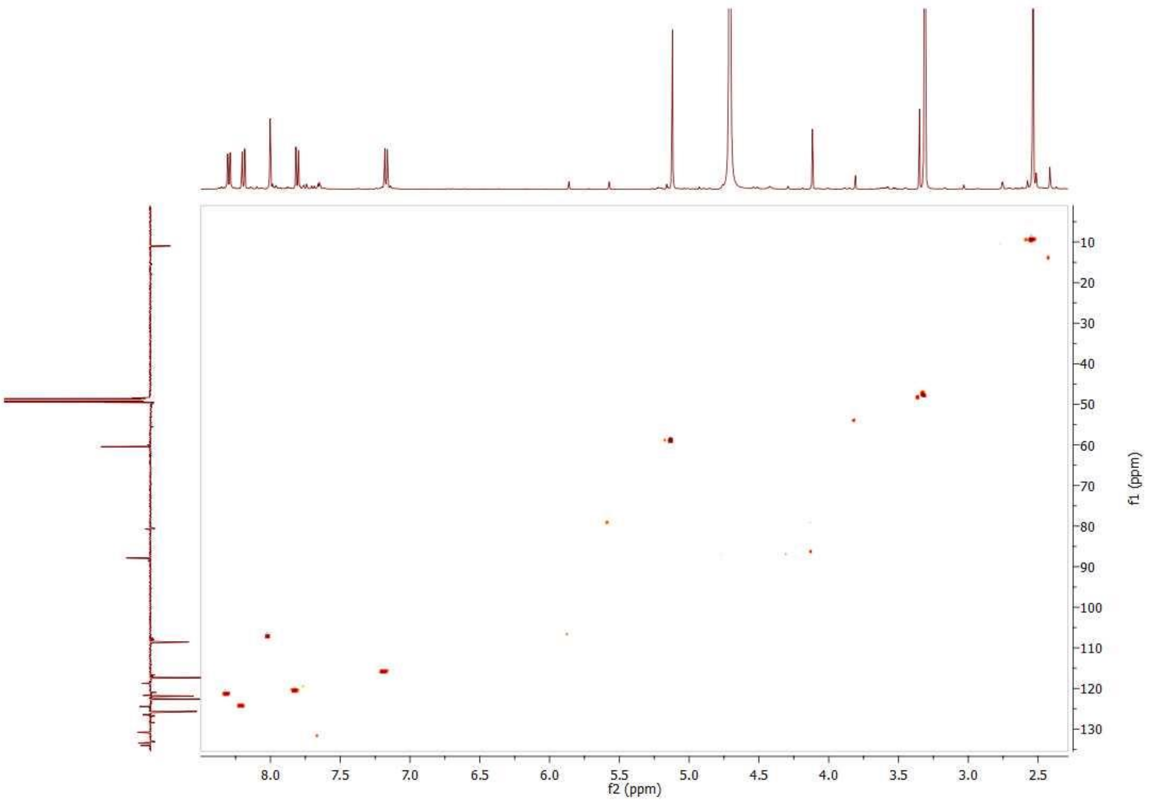

Figure S43. HSQC spectrum of compound **6** (in CD_3OD).

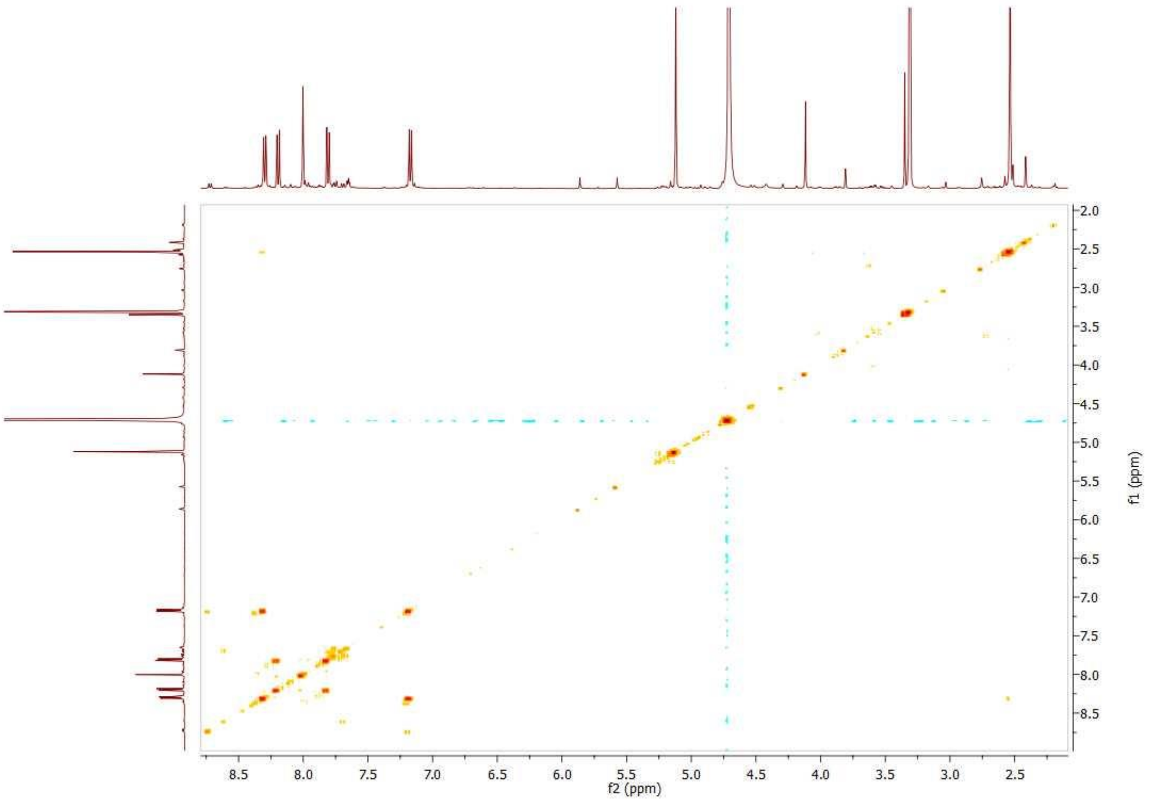

Figure S44. ^1H-^1H COSY spectrum of compound **6** (in CD_3OD).

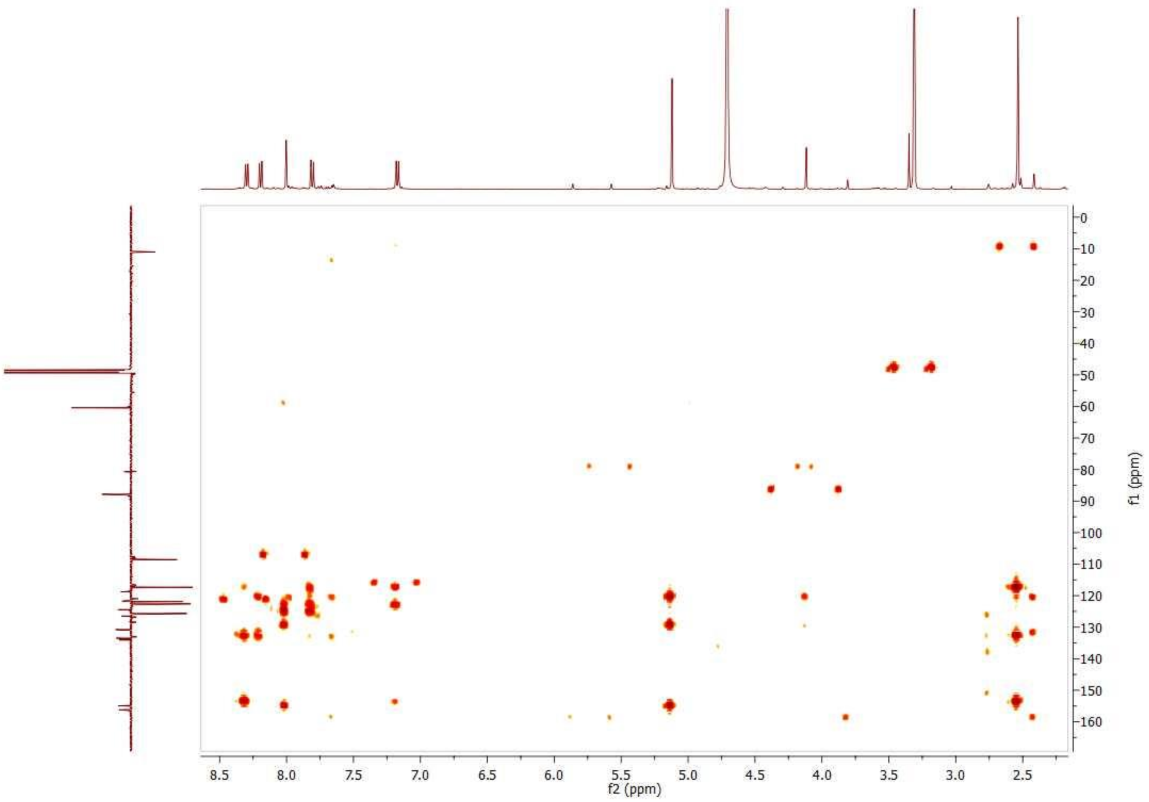

Figure S45. HMBC spectrum of compound **6** (in CD_3OD).

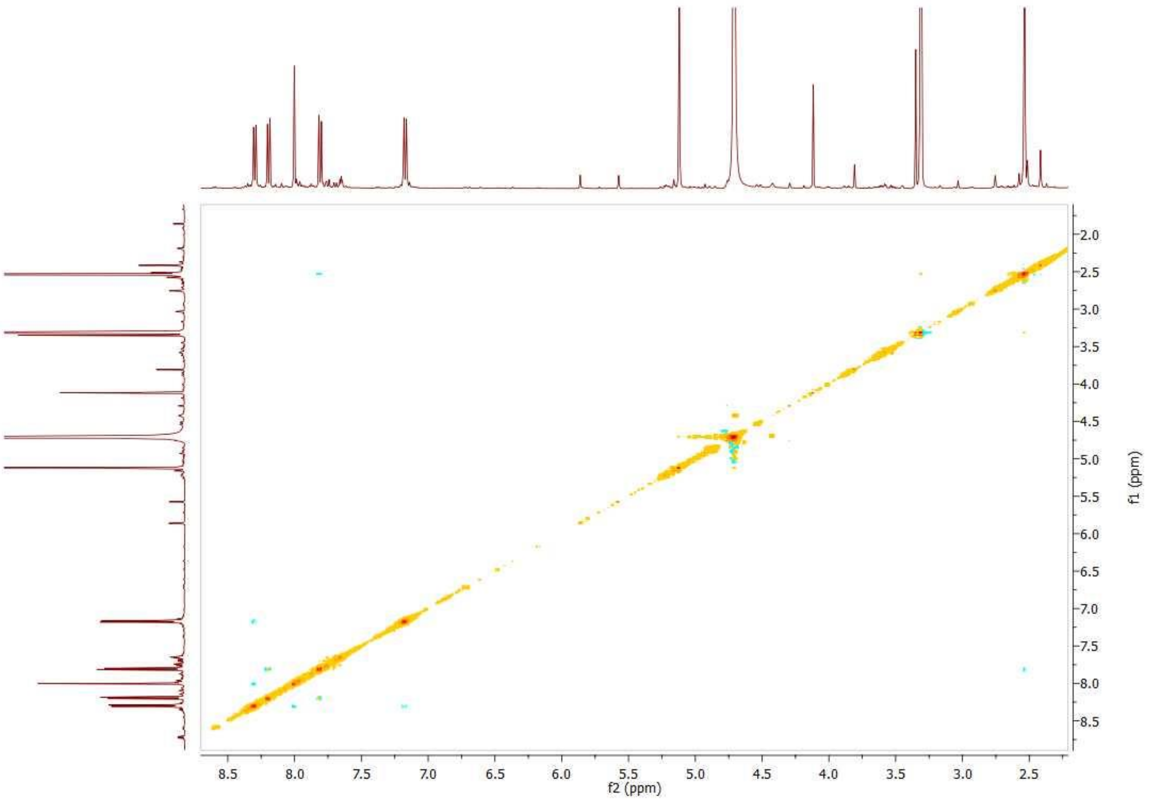

Figure S46. NOESY spectrum of compound **6** (in CD_3OD).

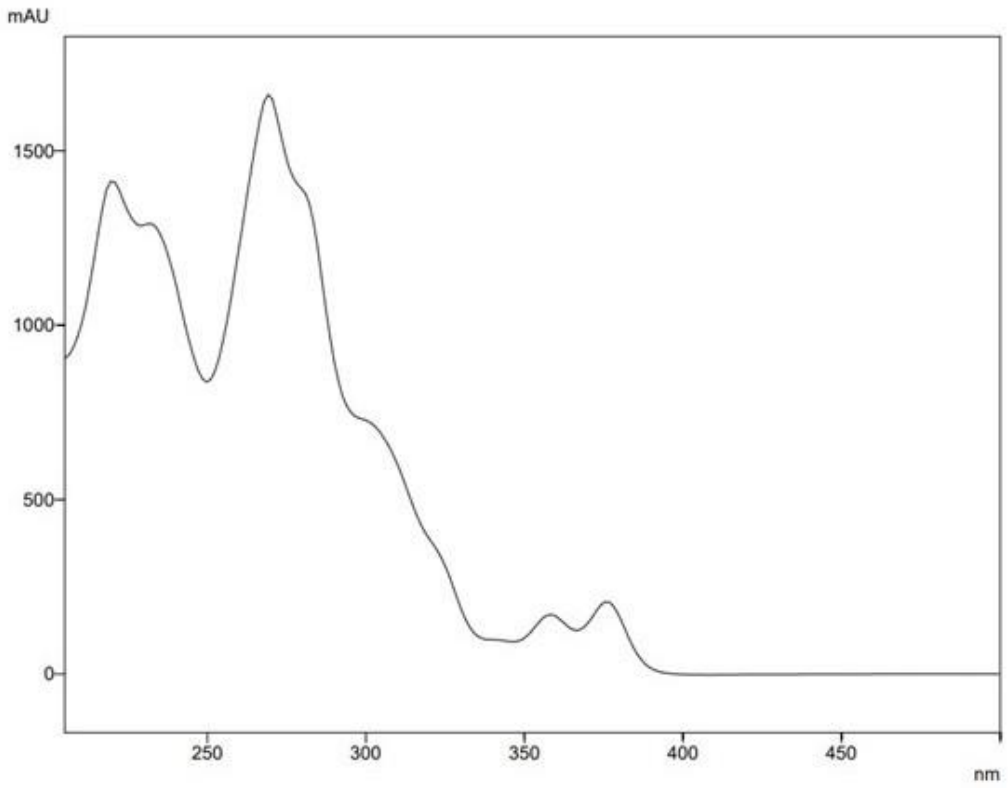

Figure S47. UV spectrum of compound **6** in MeOH.

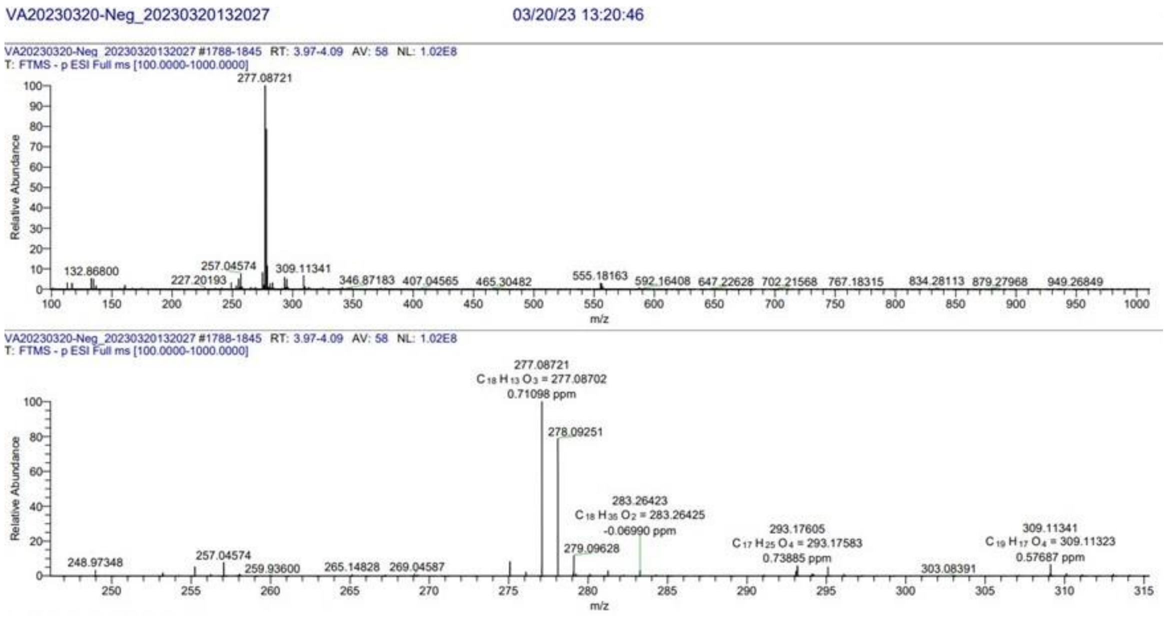

Figure S48. (–)-HRESIMS spectrum of compound **6**.

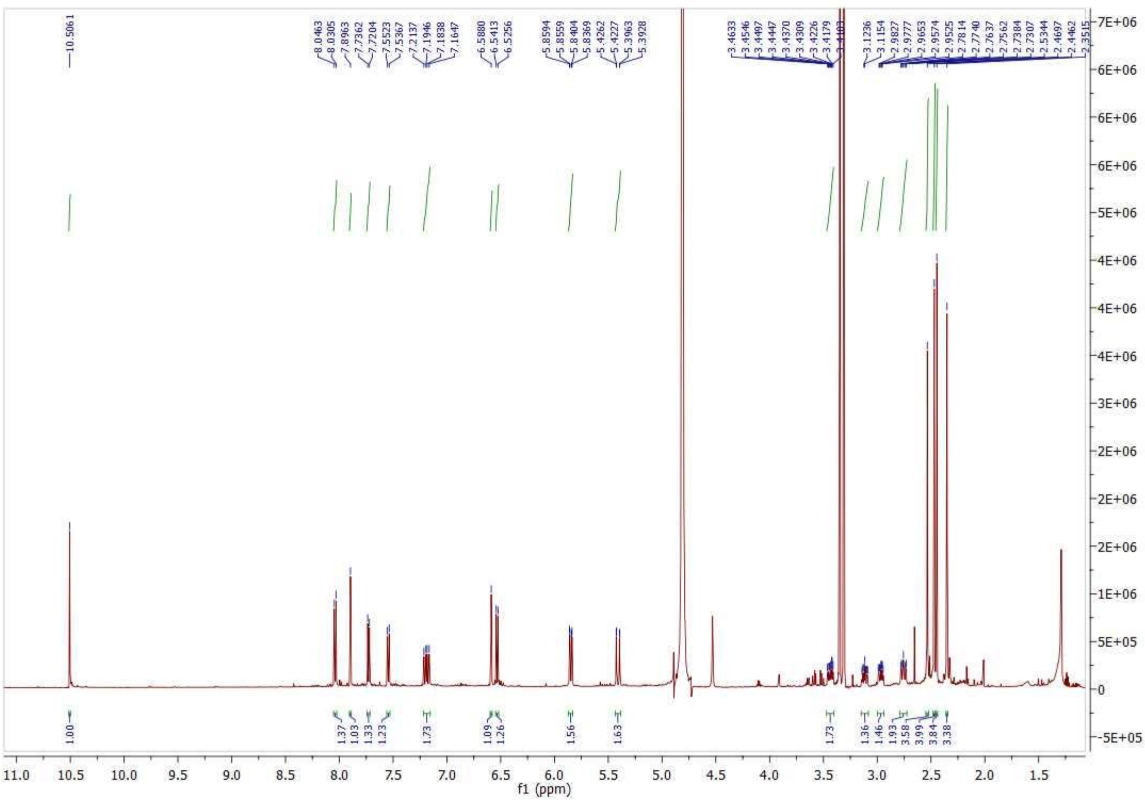

Figure S49. ^{1}H NMR spectrum of compound **7** (600 MHz, in CD_3OD).

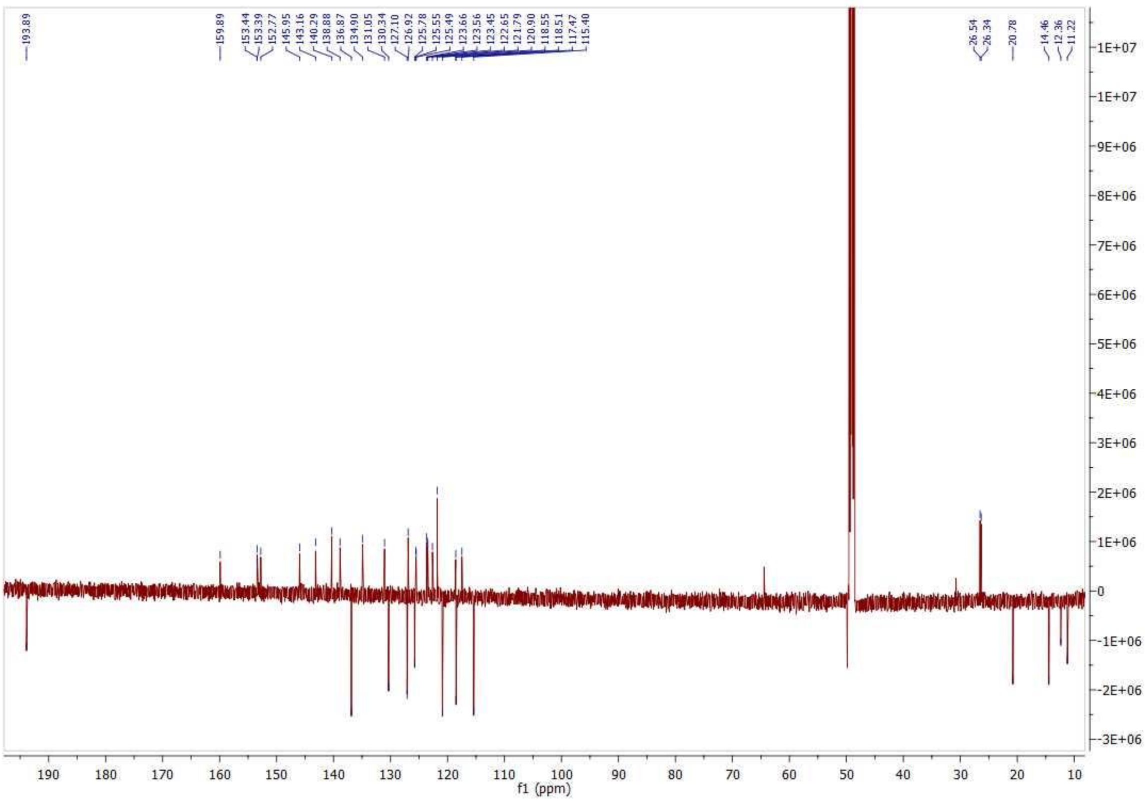

Figure S50. ^{13}C (JMOD) NMR spectrum of compound **7** (150 MHz, in CD_3OD).

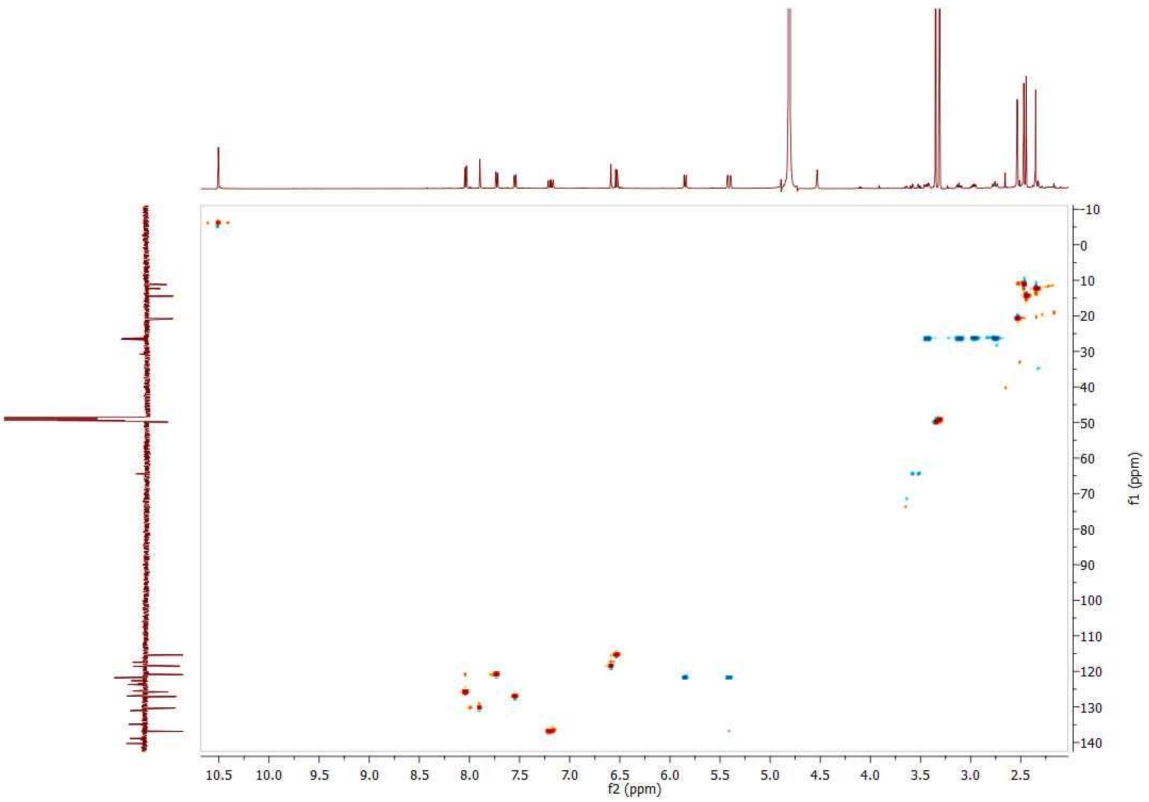

Figure S51. HSQC spectrum of compound **7** (in CD_3OD).

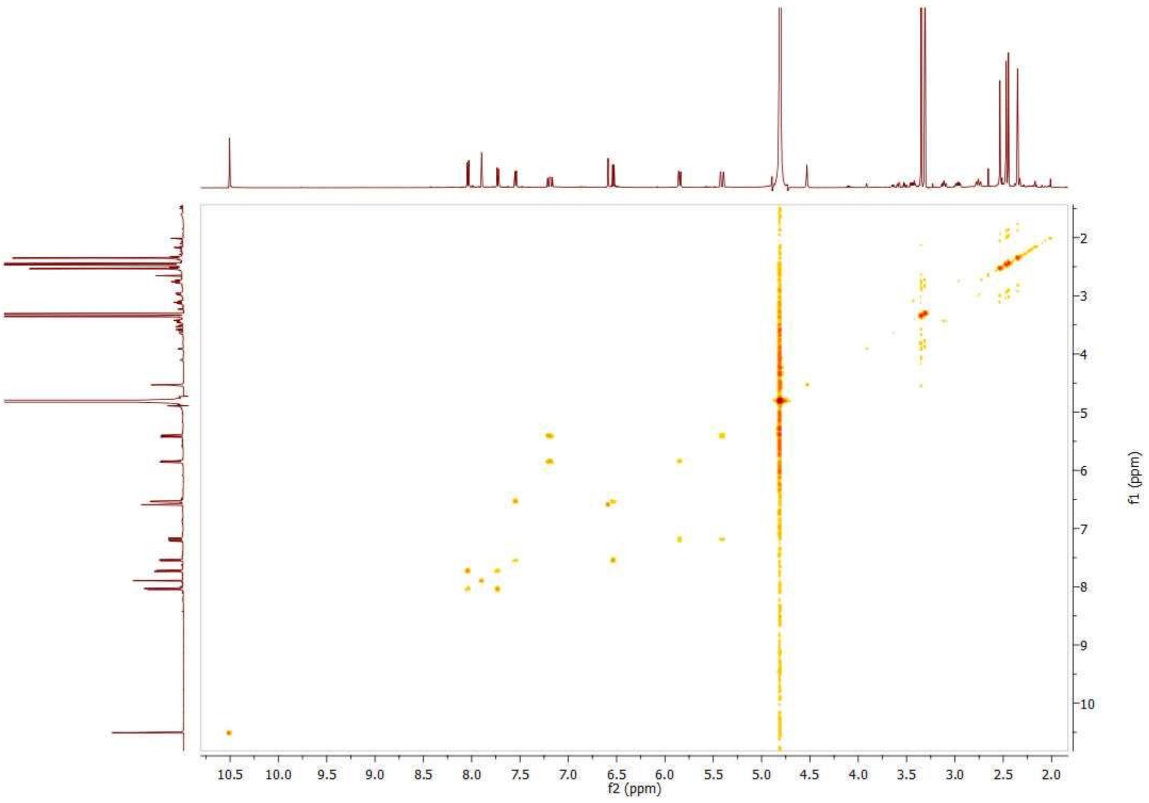

Figure S52. ^1H-^1H COSY spectrum of compound **7** (in CD_3OD).

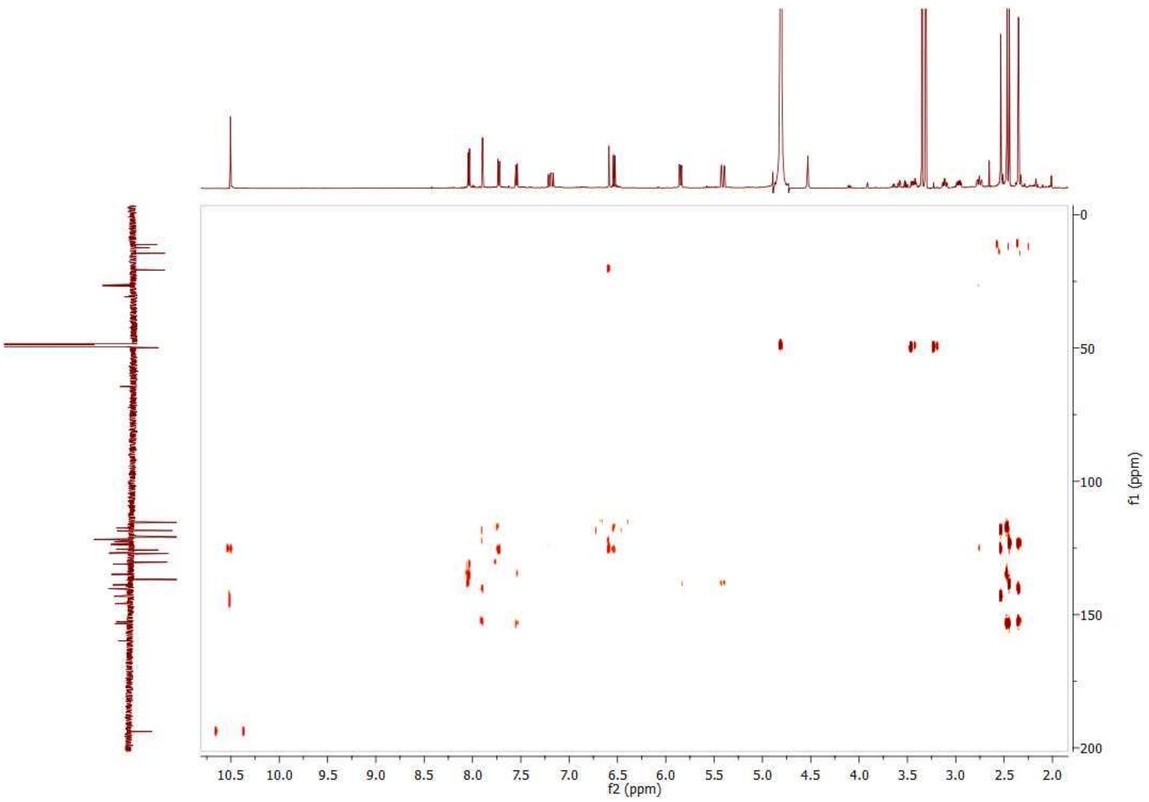

Figure S53. HMBC spectrum of compound **7** (in CD_3OD).

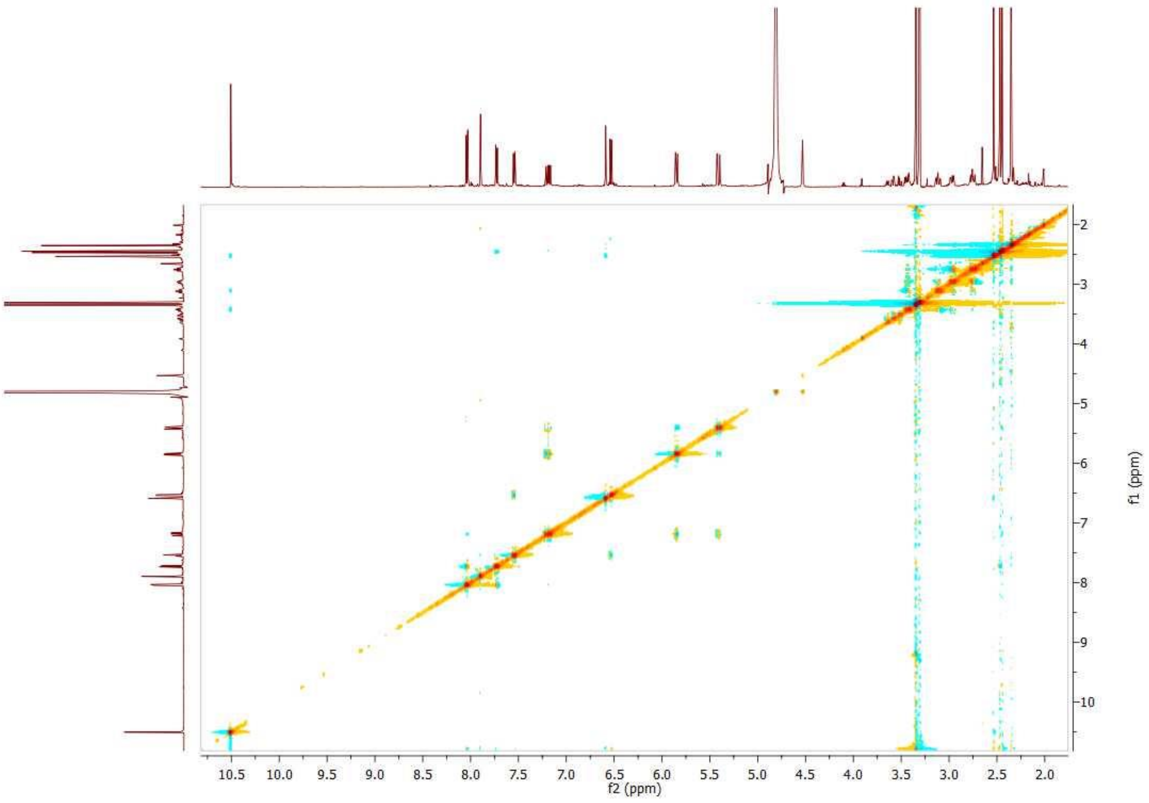

Figure S54. NOESY spectrum of compound **7** (in CD_3OD).

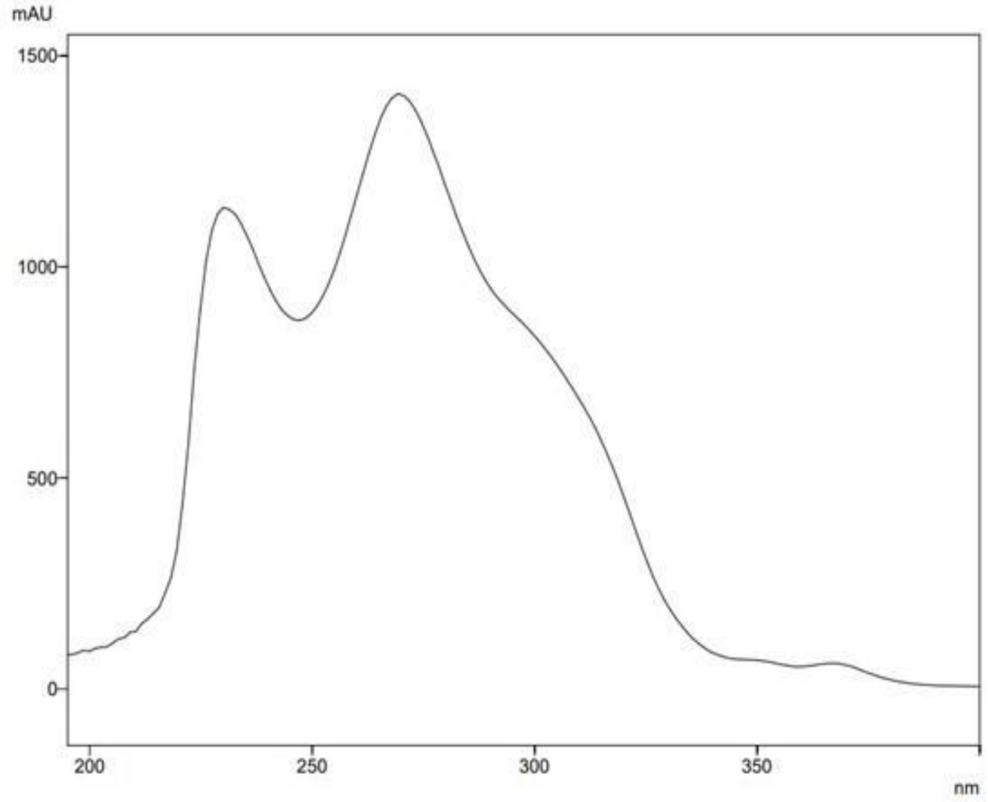

Figure S55. UV spectrum of compound **7** in MeOH.

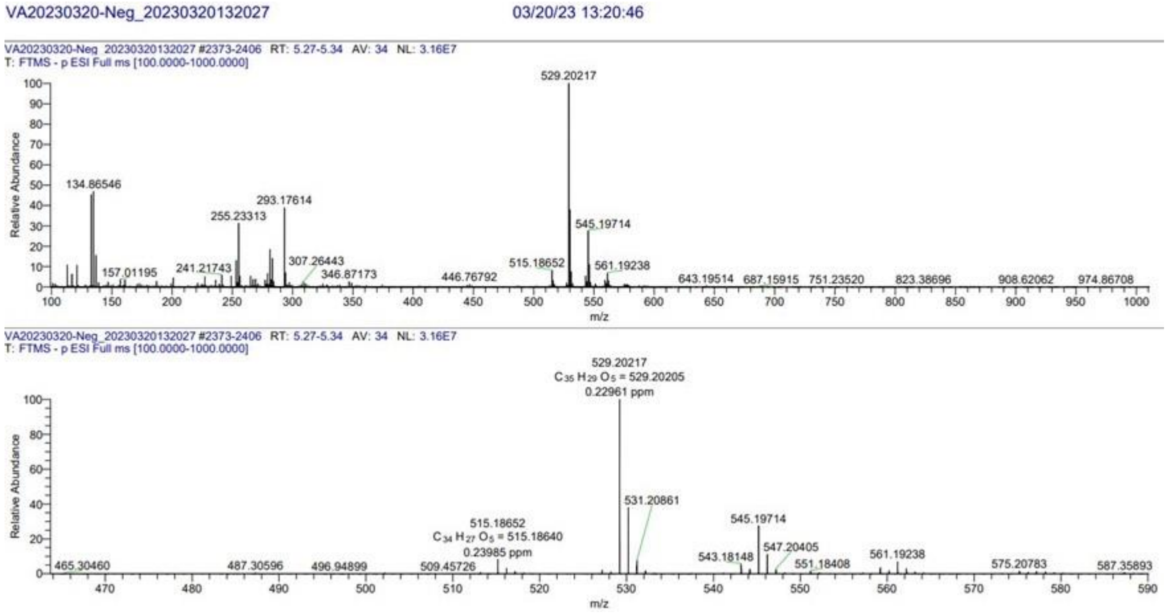

Figure S56. (−)-HRESIMS spectrum of compound **7**.

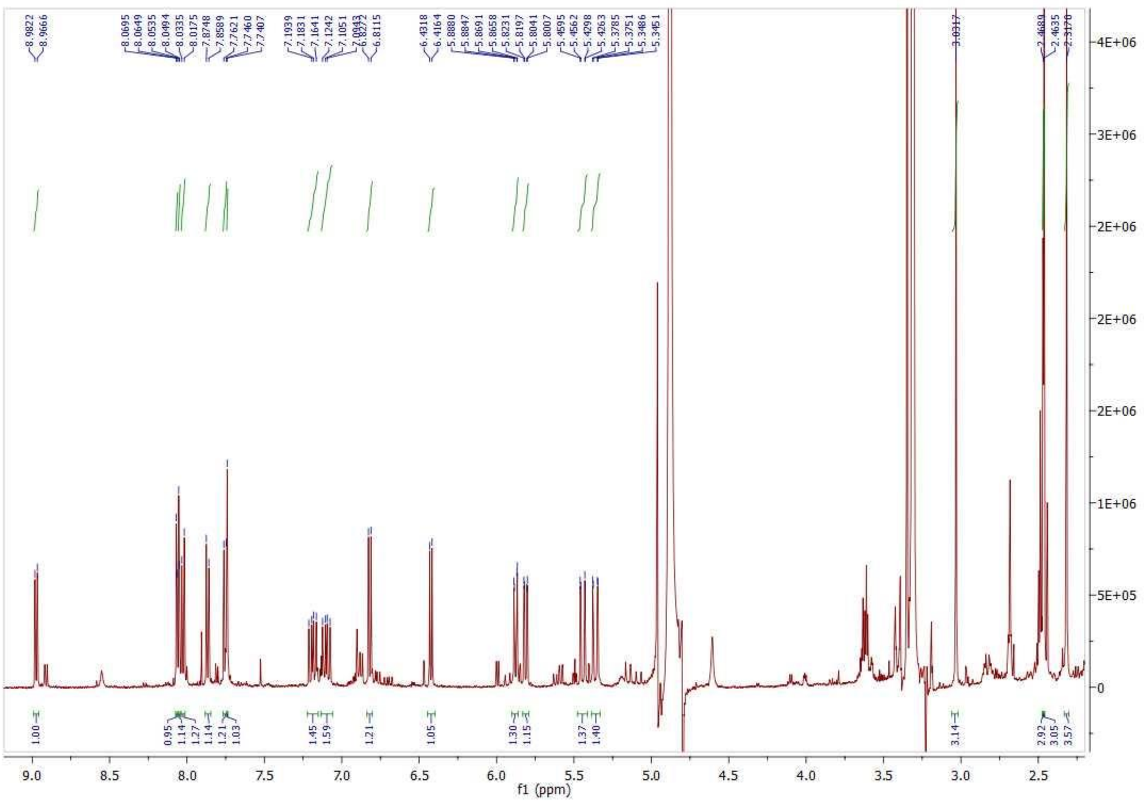

Figure S57. ^{1}H NMR spectrum of compound **8** (600 MHz, in CD_3OD).

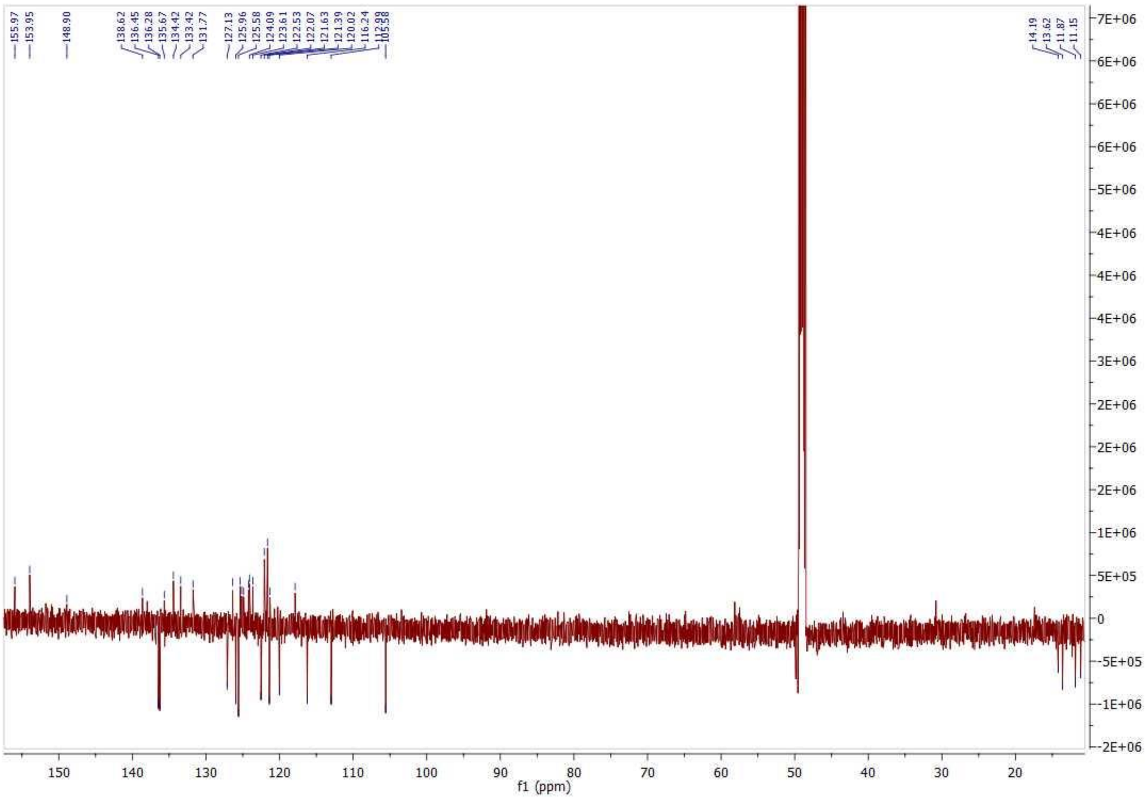

Figure S58. ^{13}C (JMOD) NMR spectrum of compound **8** (150 MHz, in CD_3OD).

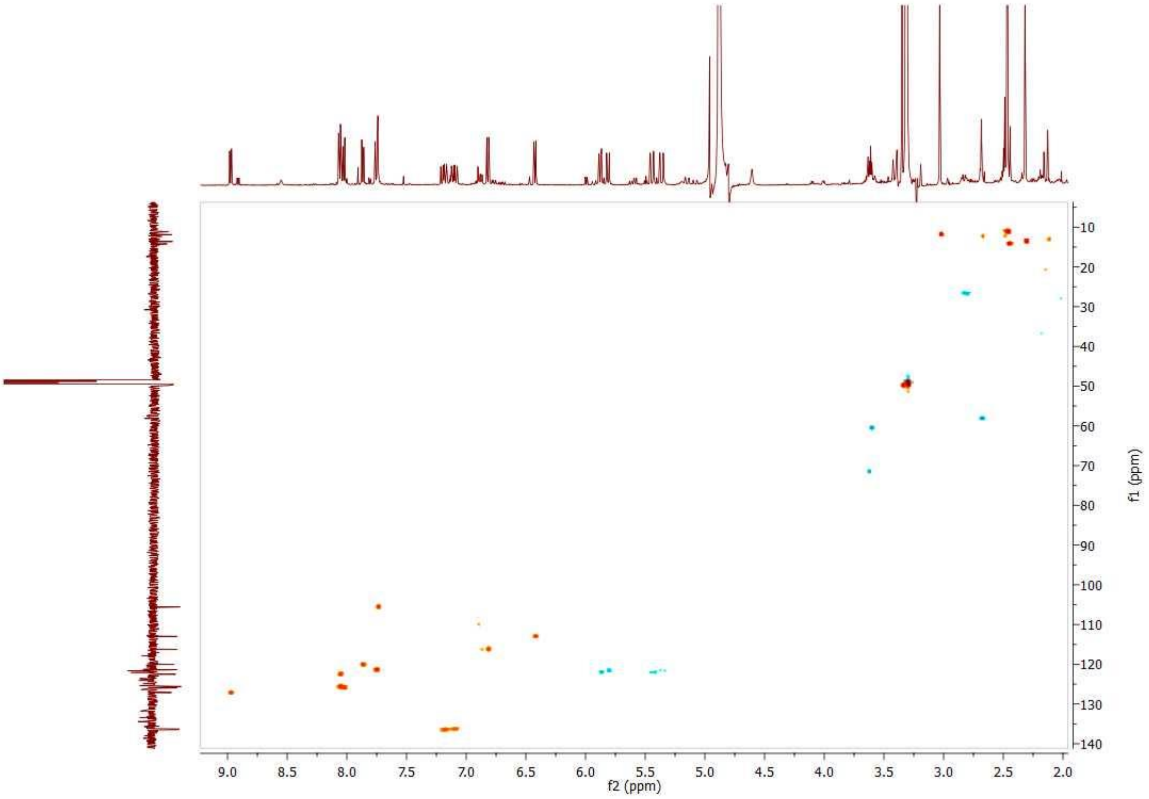

Figure S59. HSQC spectrum of compound **8** (in CD_3OD).

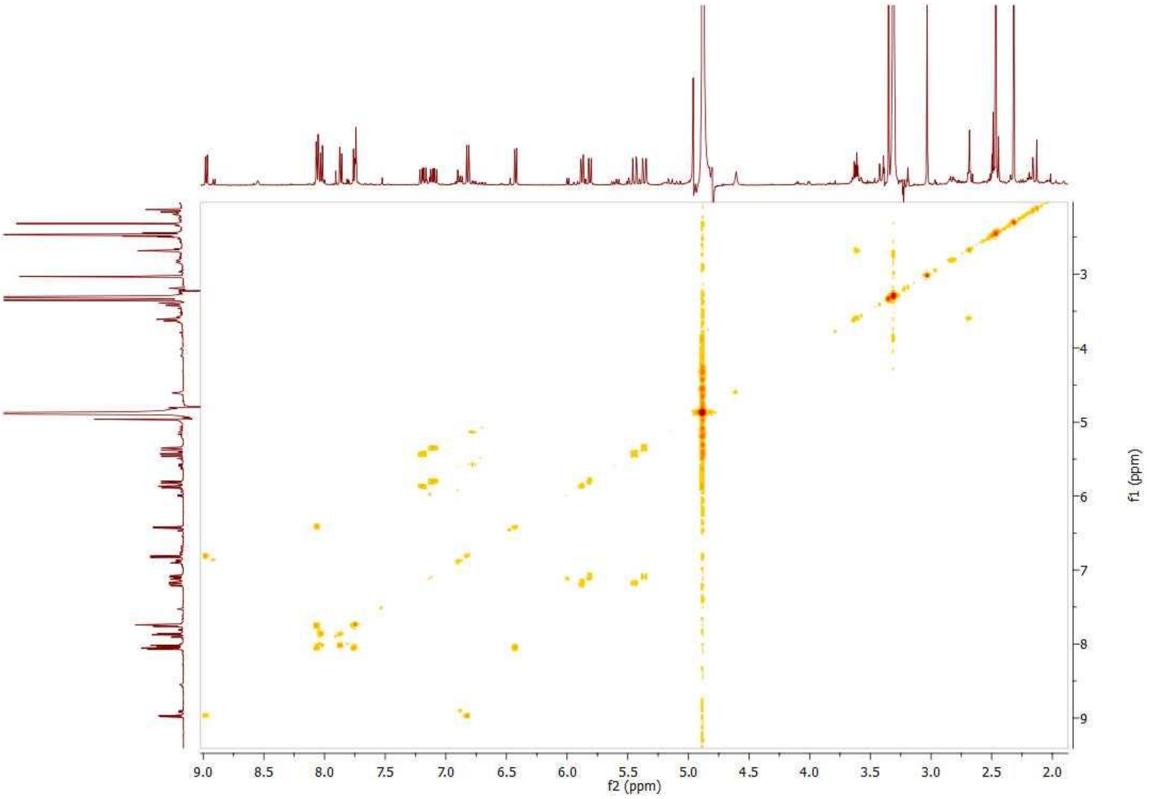

Figure S60. ^1H-^1H COSY spectrum of compound **8** (in CD_3OD).

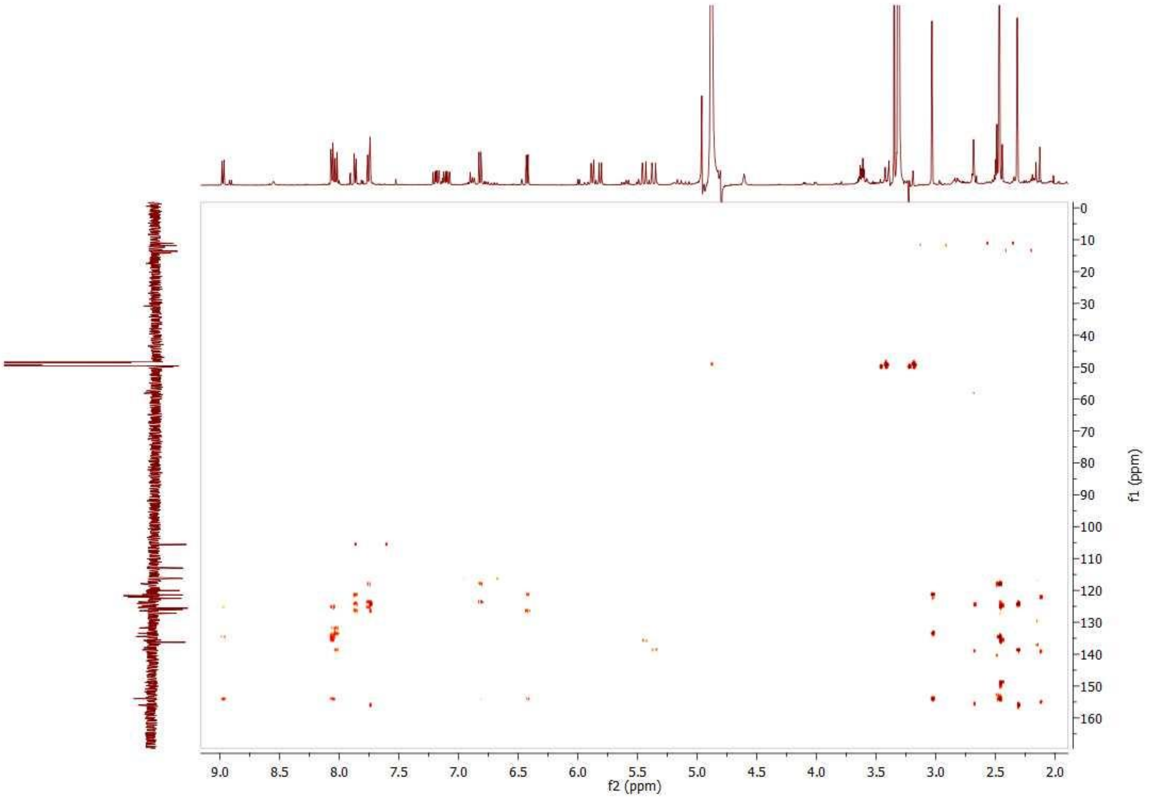

Figure S61. HMBC spectrum of compound **8** (in CD_3OD).

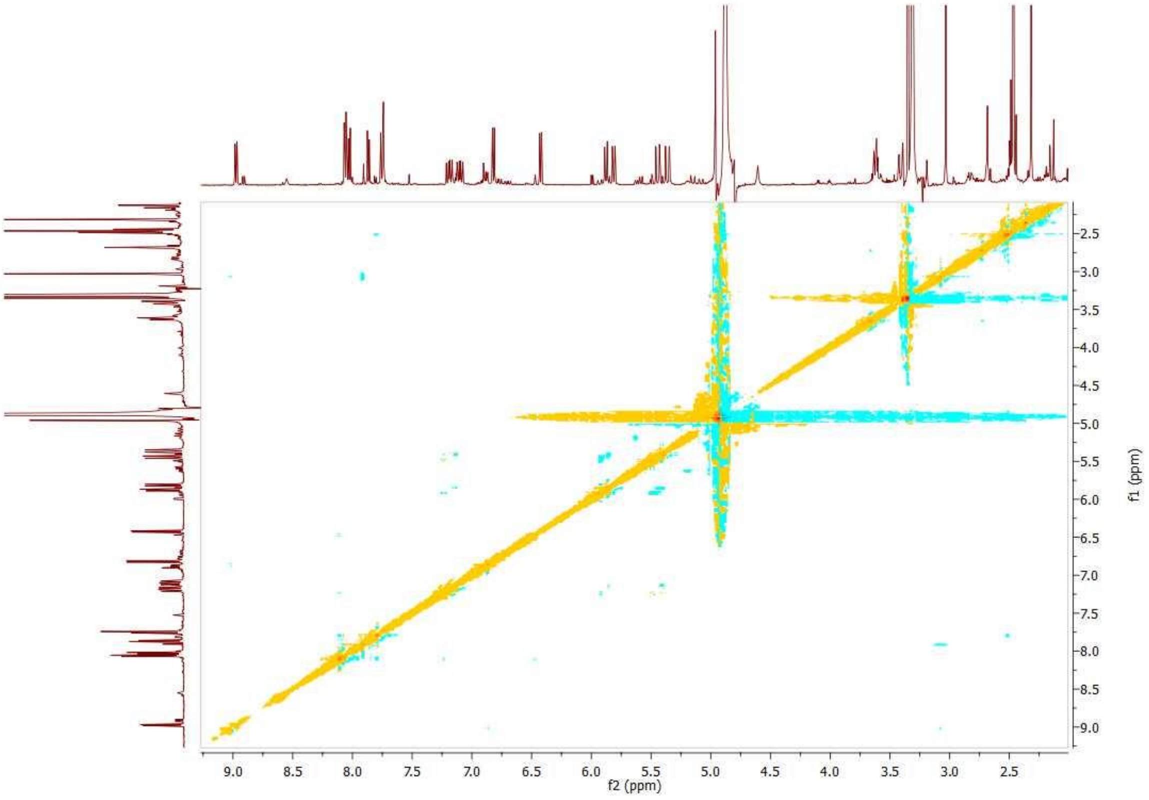

Figure S62. NOESY spectrum of compound **8** (in CD_3OD).

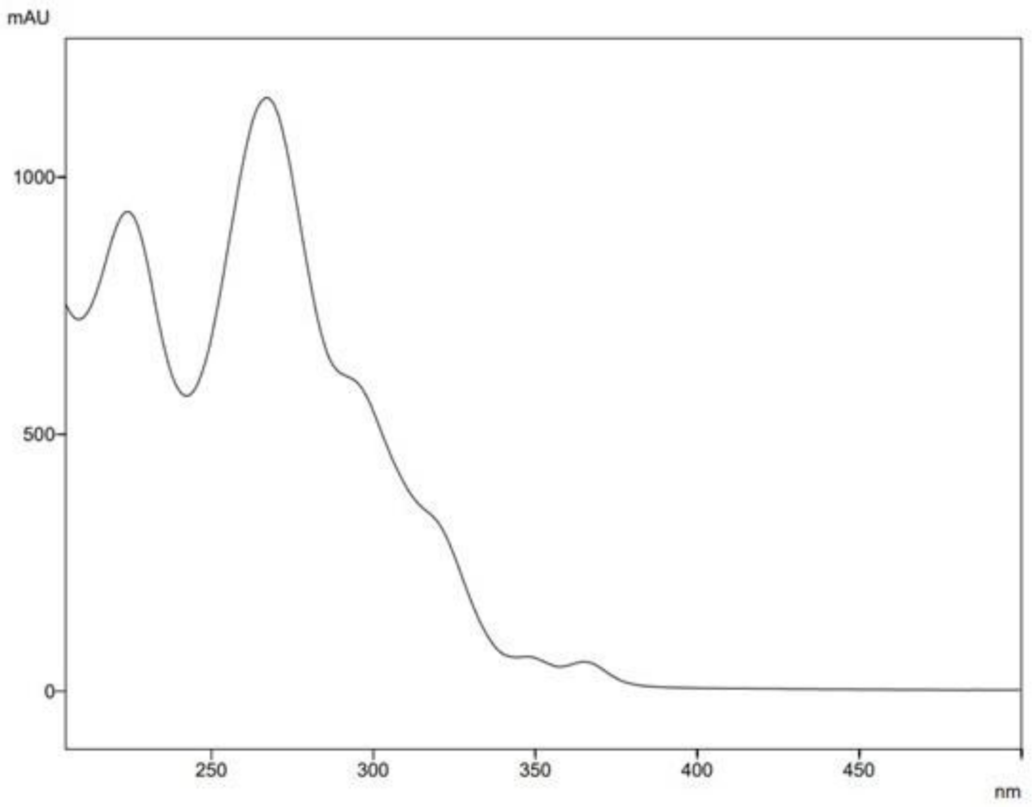

Figure S63. UV spectrum of compound **8** in MeOH.

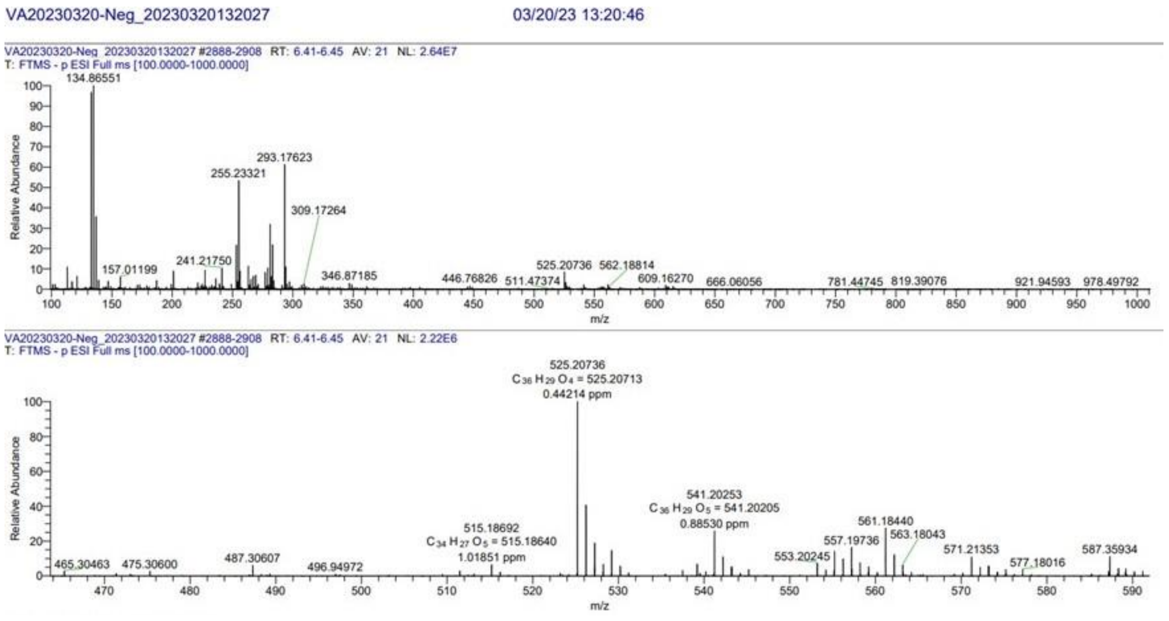

Figure S64. (−)-HRESIMS spectrum of compound **8**.

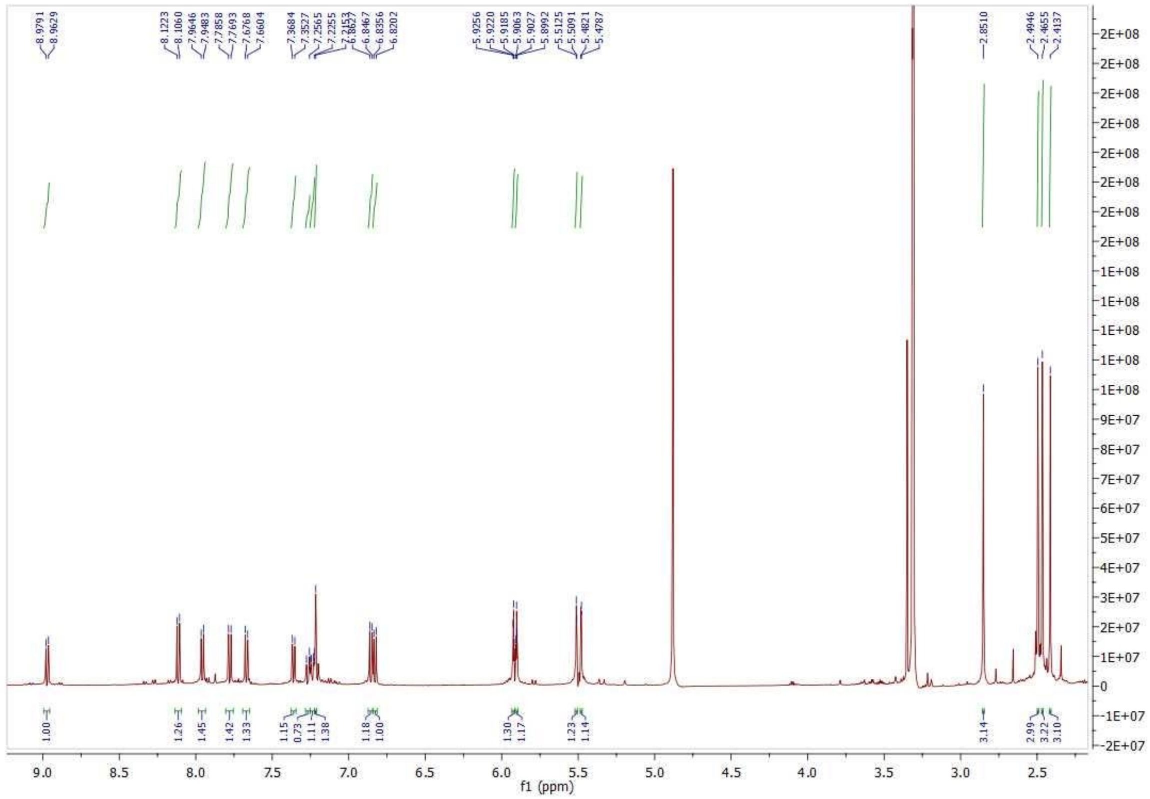

Figure S65. ^{1}H NMR spectrum of compound **9** (600 MHz, in CD_3OD).

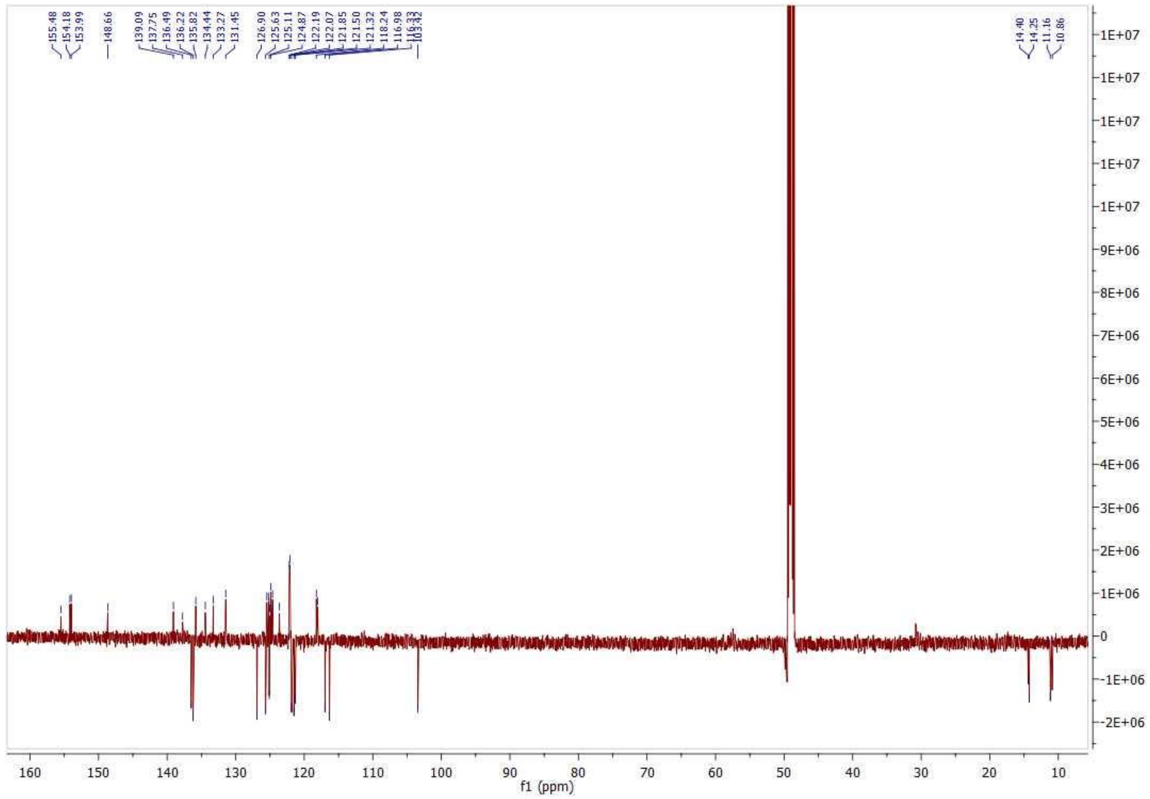

Figure S66. ^{13}C (JMOD) NMR spectrum of compound **9** (150 MHz, in CD_3OD).

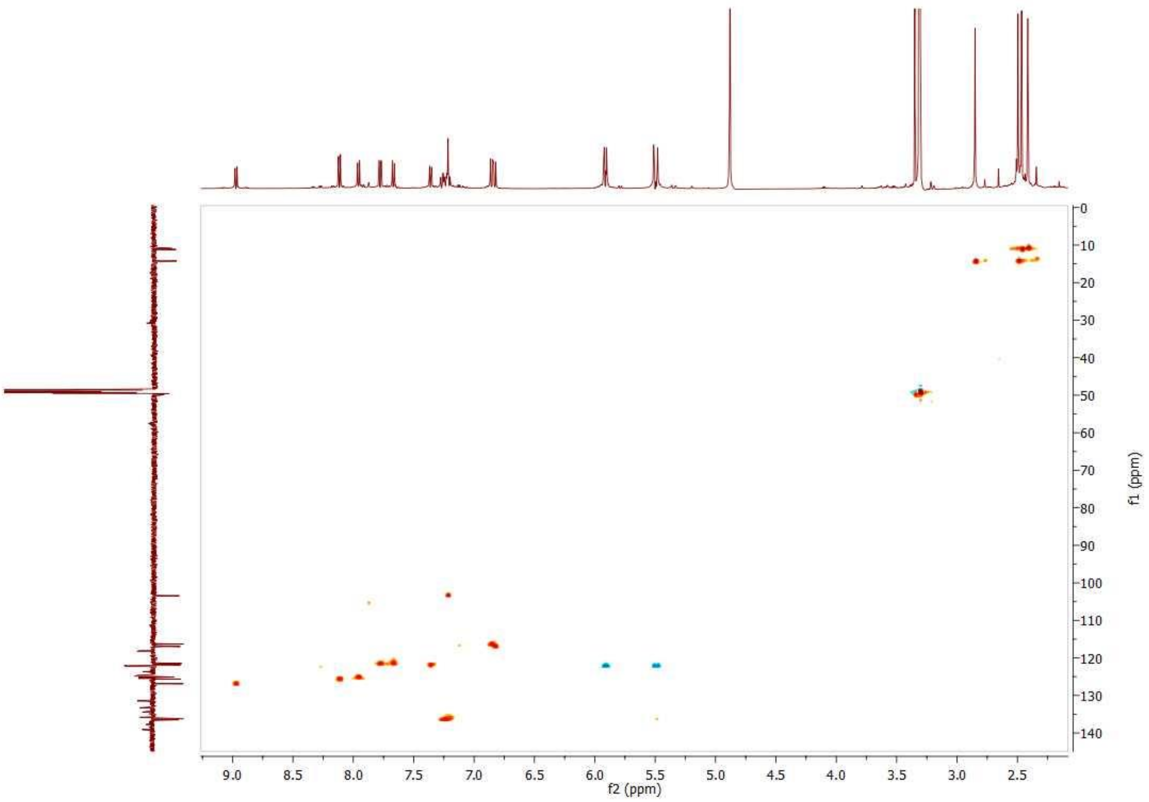

Figure S67. HSQC spectrum of compound **9** (in CD_3OD).

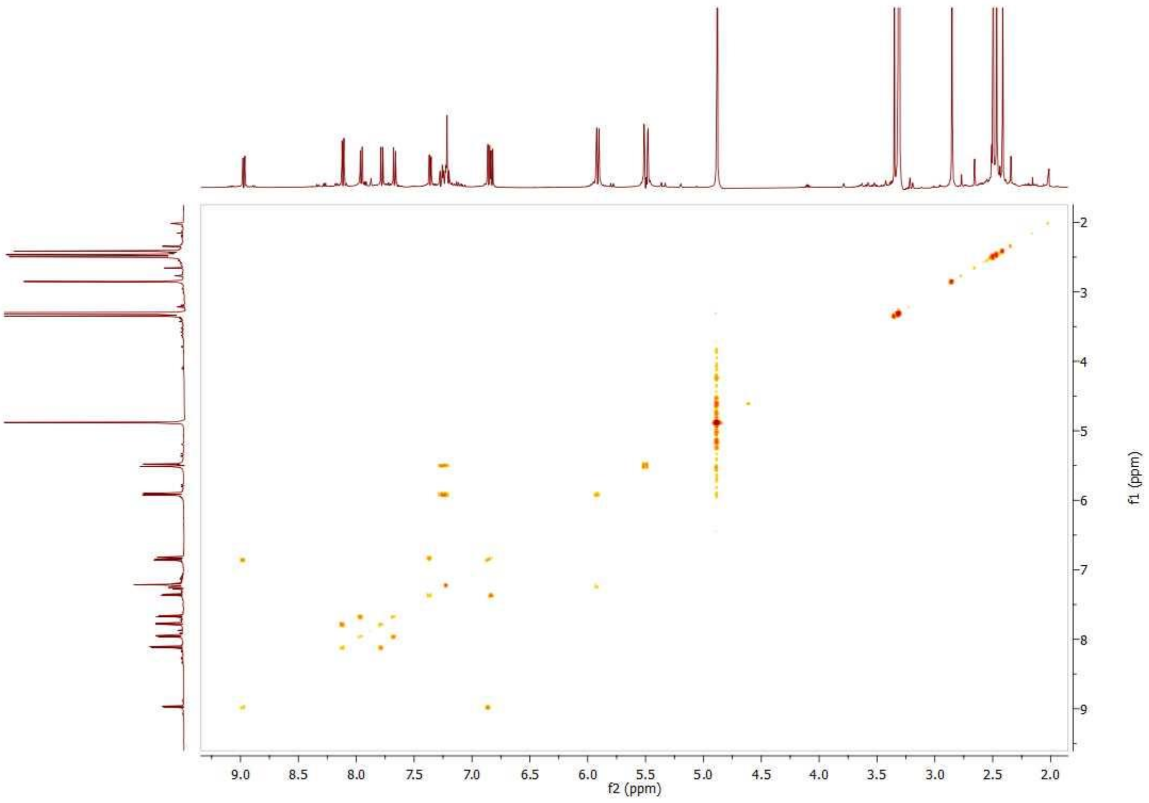

Figure S68. ^1H-^1H COSY spectrum of compound **9** (in CD_3OD).

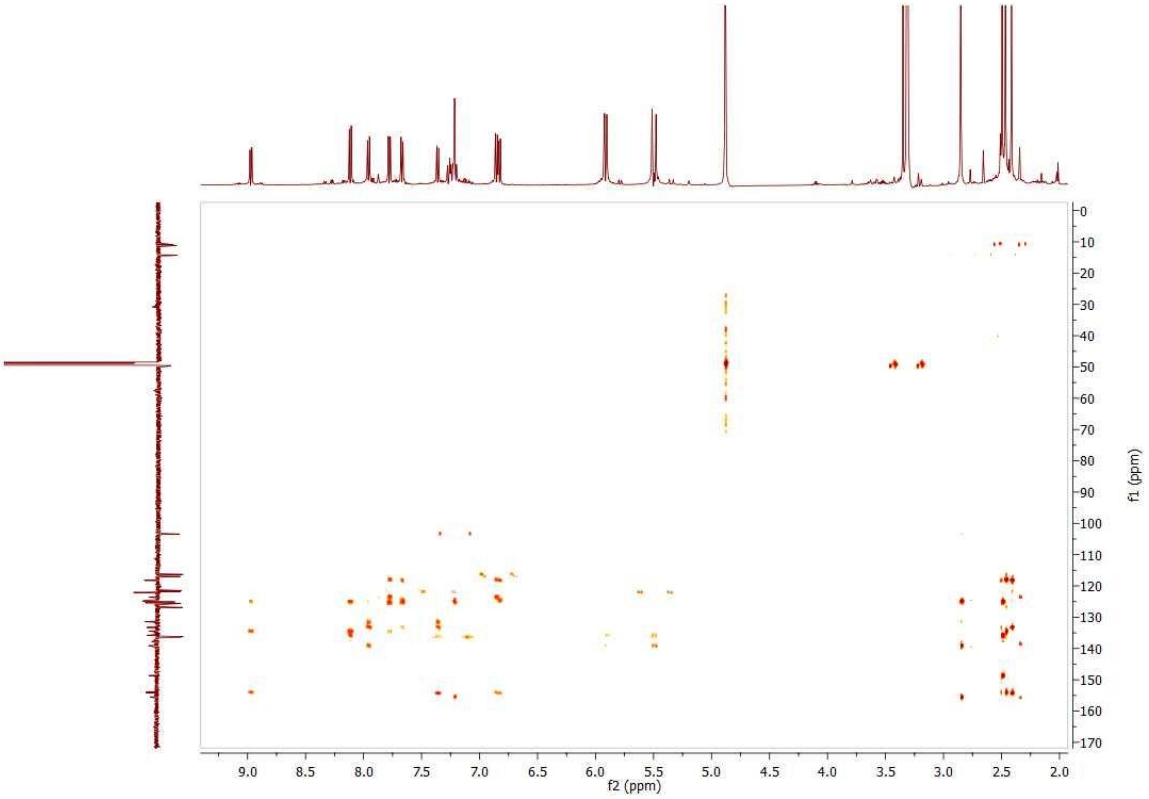

Figure S69. HMBC spectrum of compound **9** (in CD_3OD).

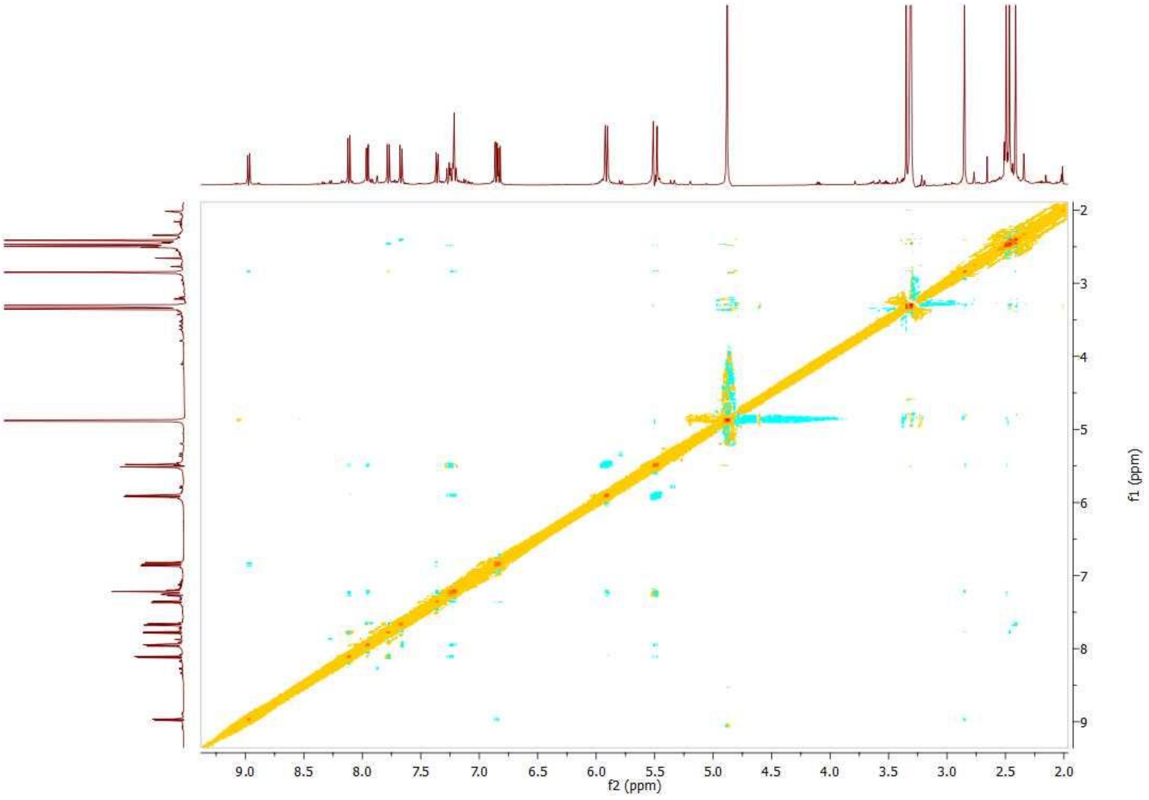

Figure S70. NOESY spectrum of compound **9** (in CD_3OD).

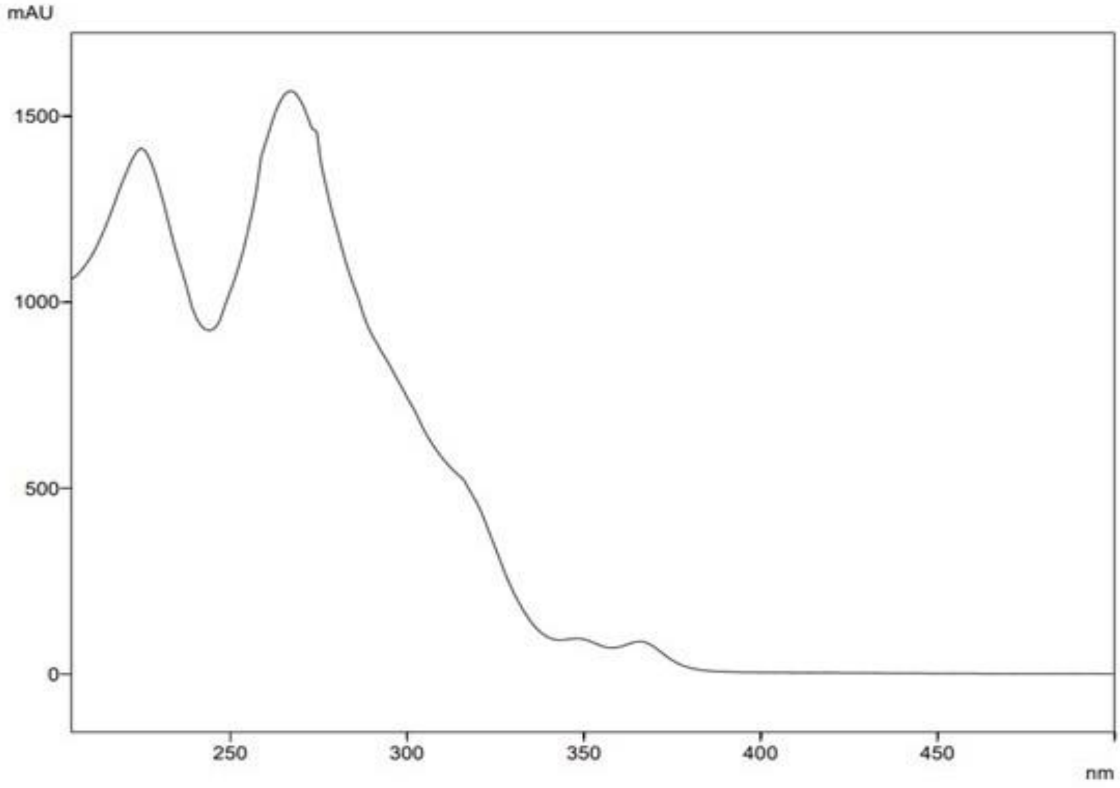

Figure S71. UV spectrum of compound **9** in MeOH.

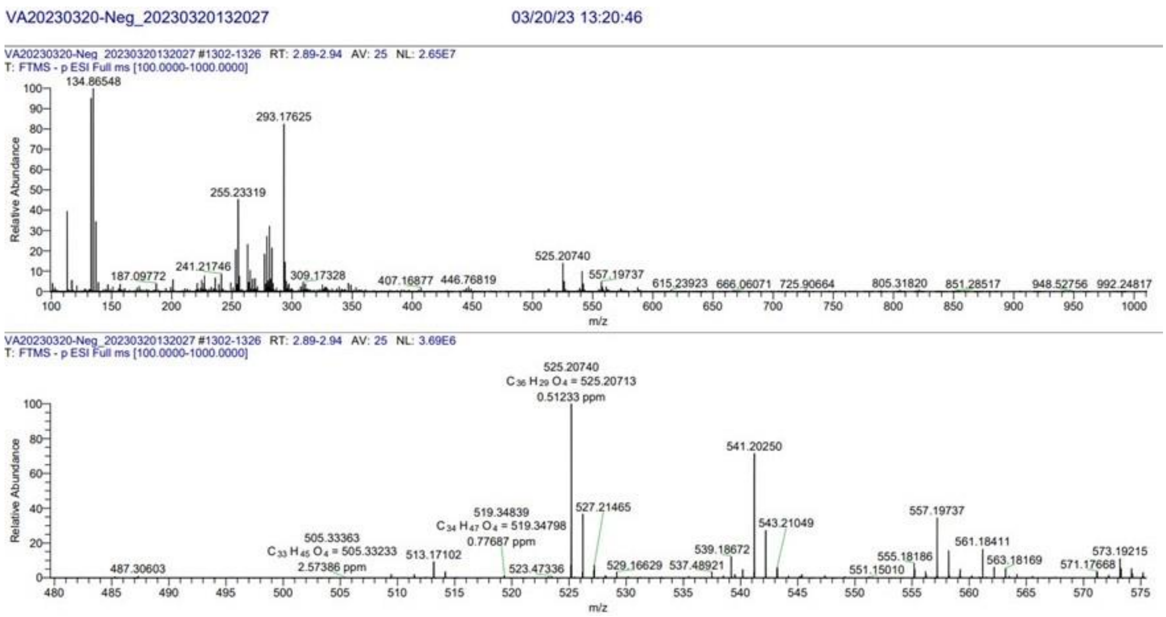

Figure S72. (−)-HRESIMS spectrum of compound **9**.

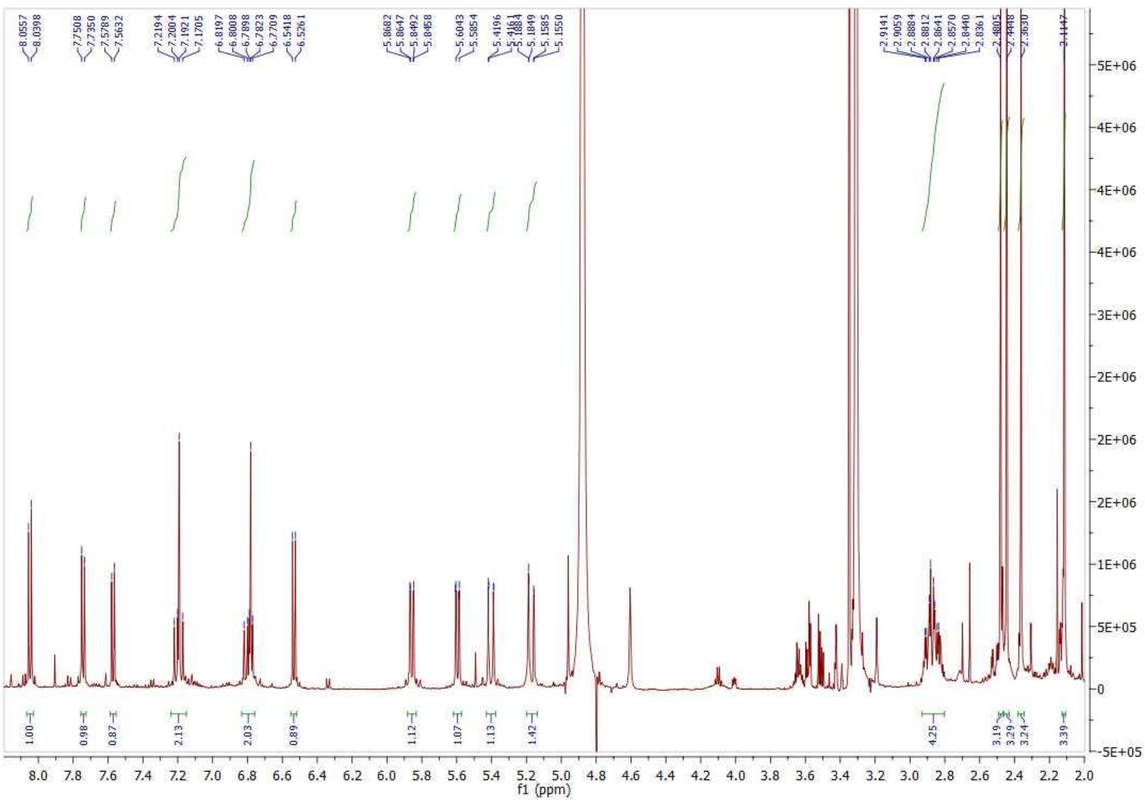

Figure S73. ^{1}H NMR spectrum of compound **10** (600 MHz, in CD_3OD).

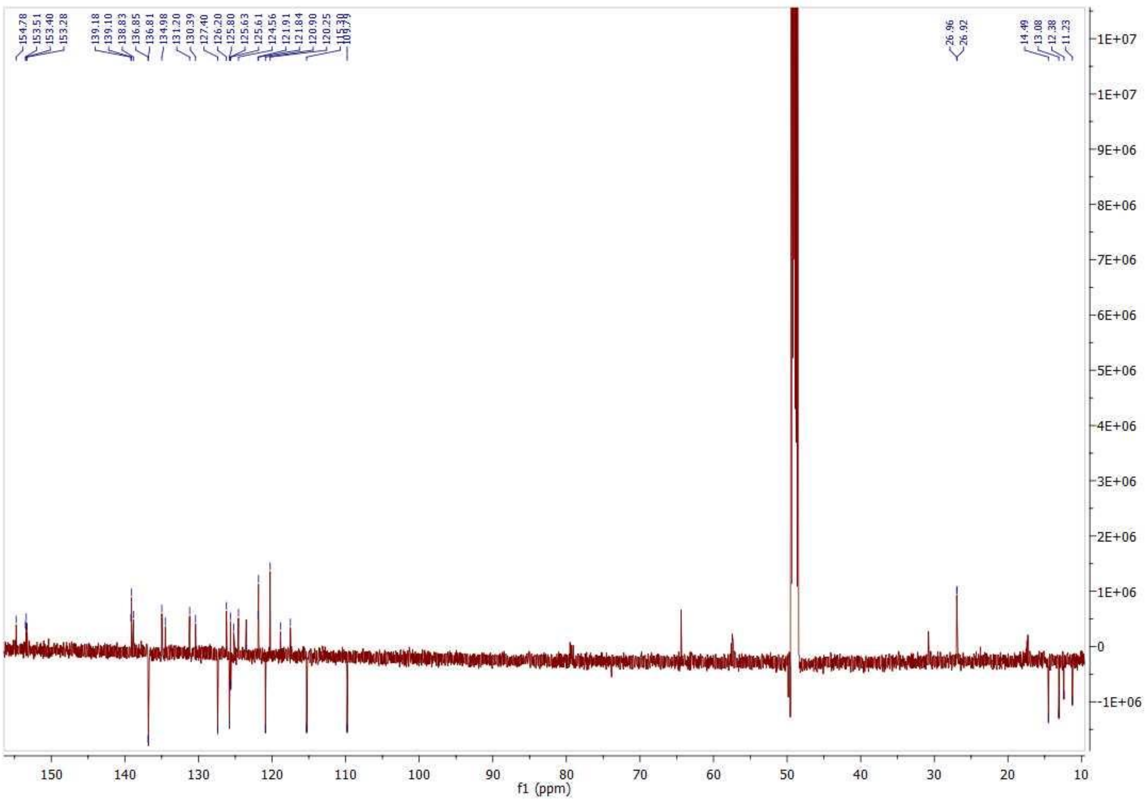

Figure S74. ^{13}C (JMOD) NMR spectrum of compound **10** (150 MHz, in CD_3OD).

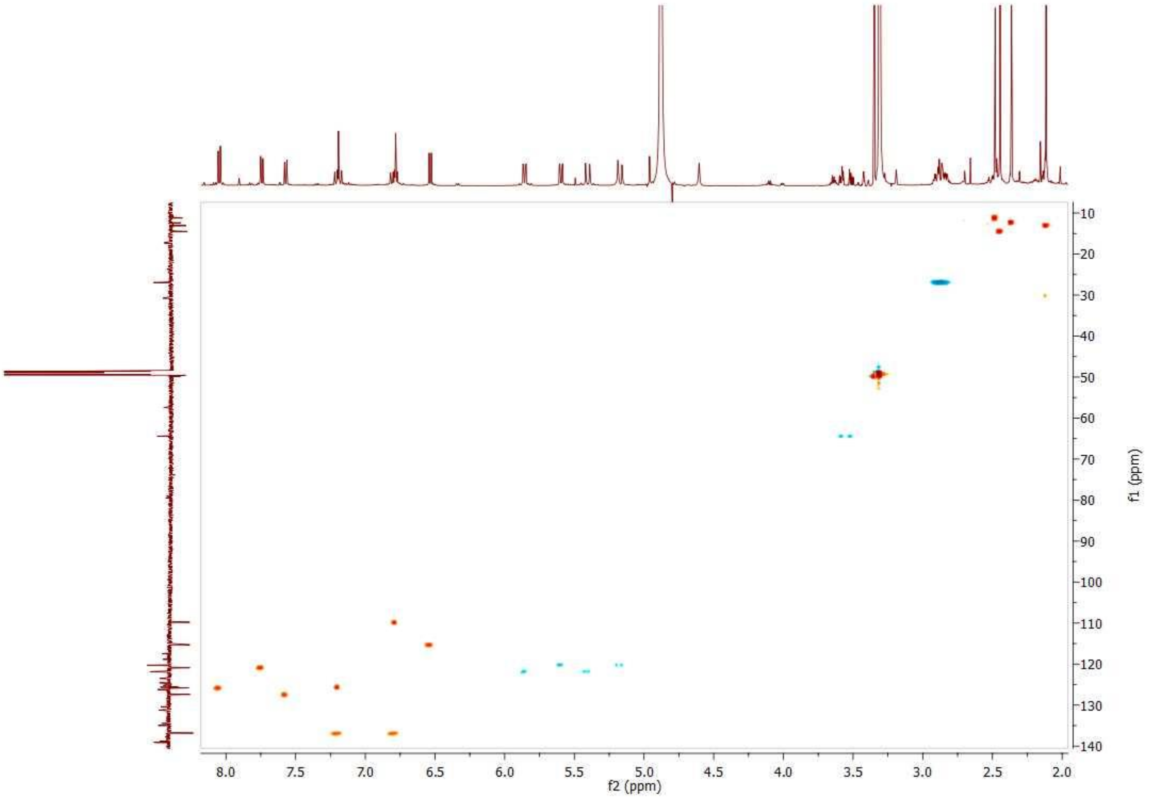

Figure S75. HSQC spectrum of compound **10** (in CD_3OD).

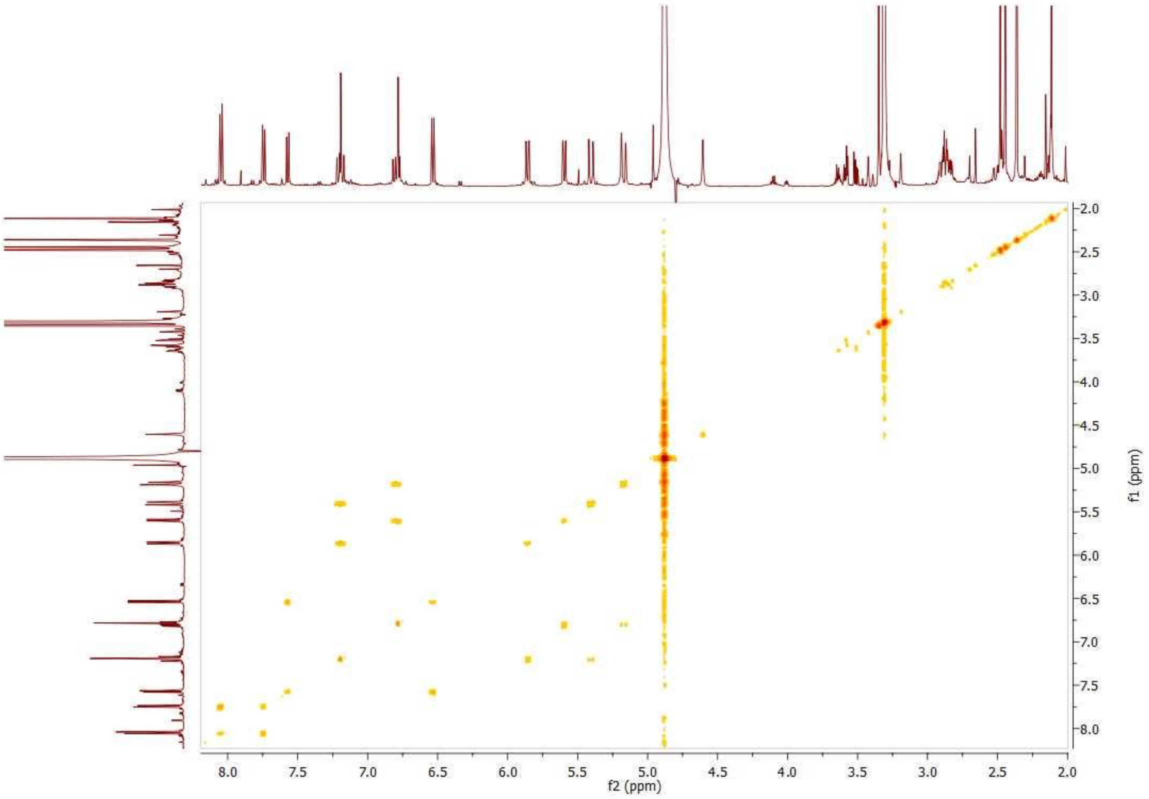

Figure S76. ^1H-^1H COSY spectrum of compound **10** (in CD_3OD).

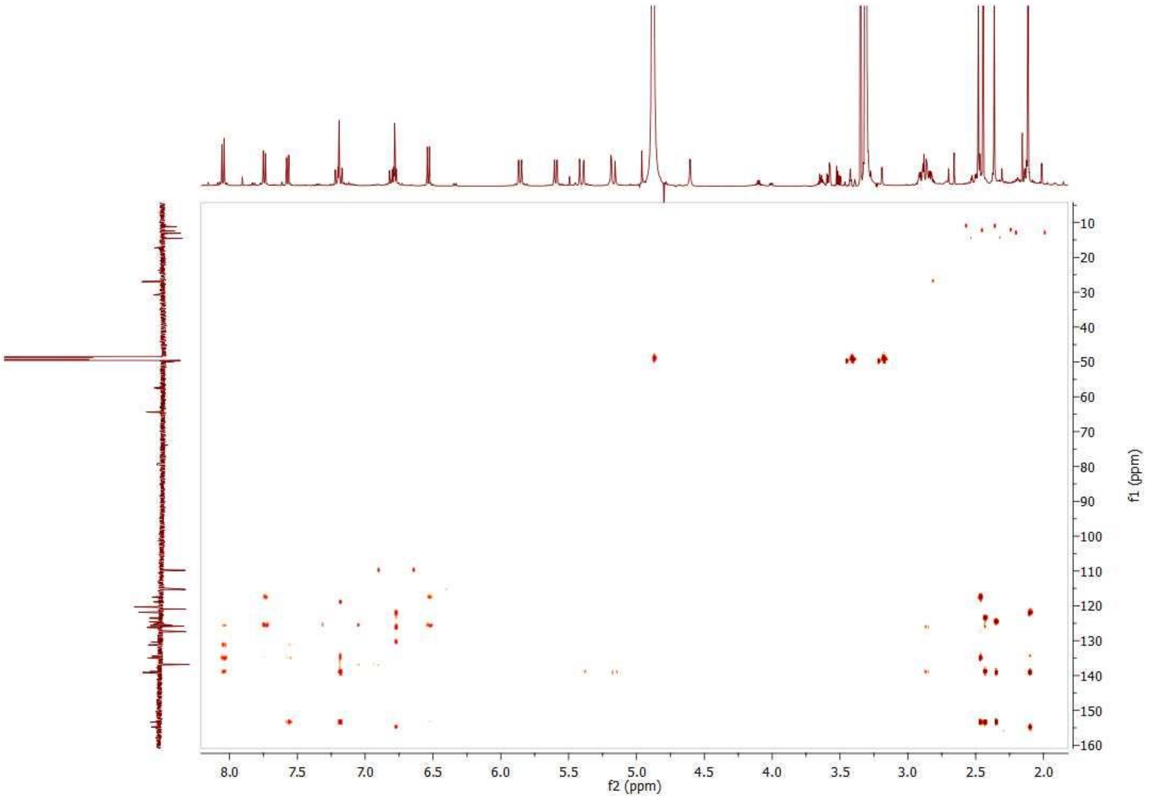

Figure S77. HMBC spectrum of compound **10** (in CD_3OD).

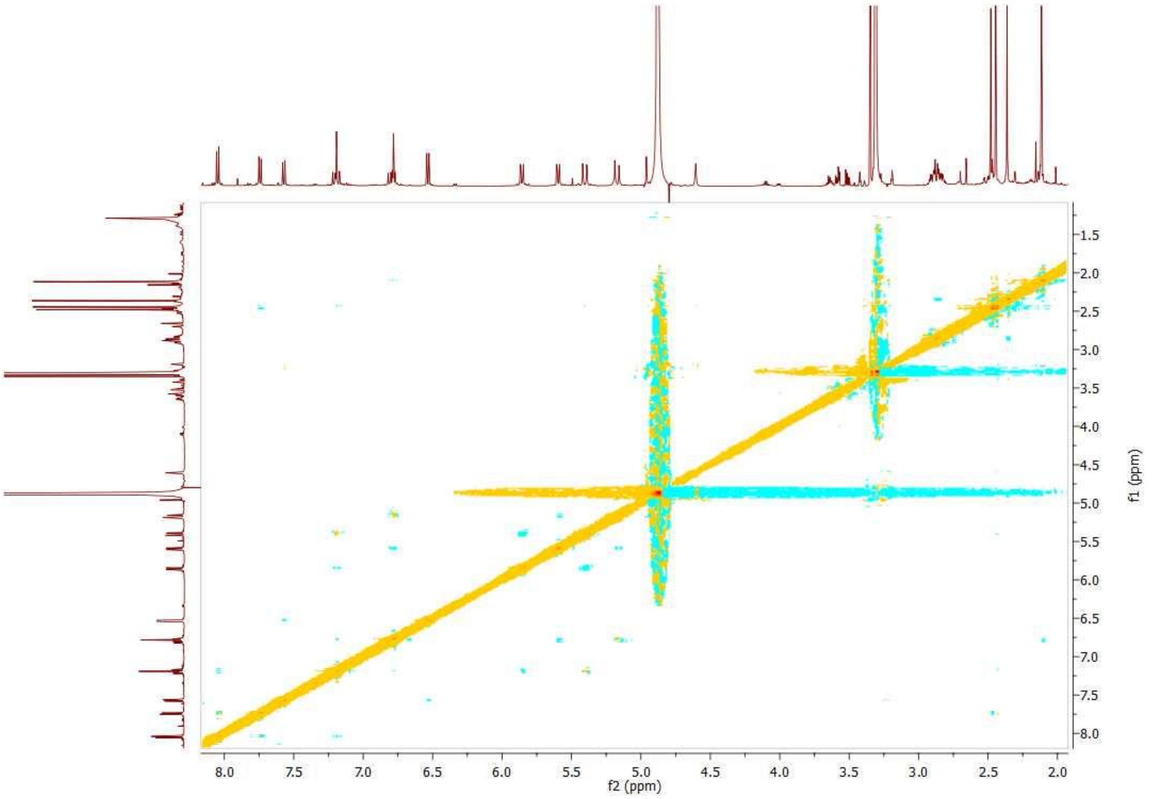

Figure S78. NOESY spectrum of compound **10** (in CD_3OD).

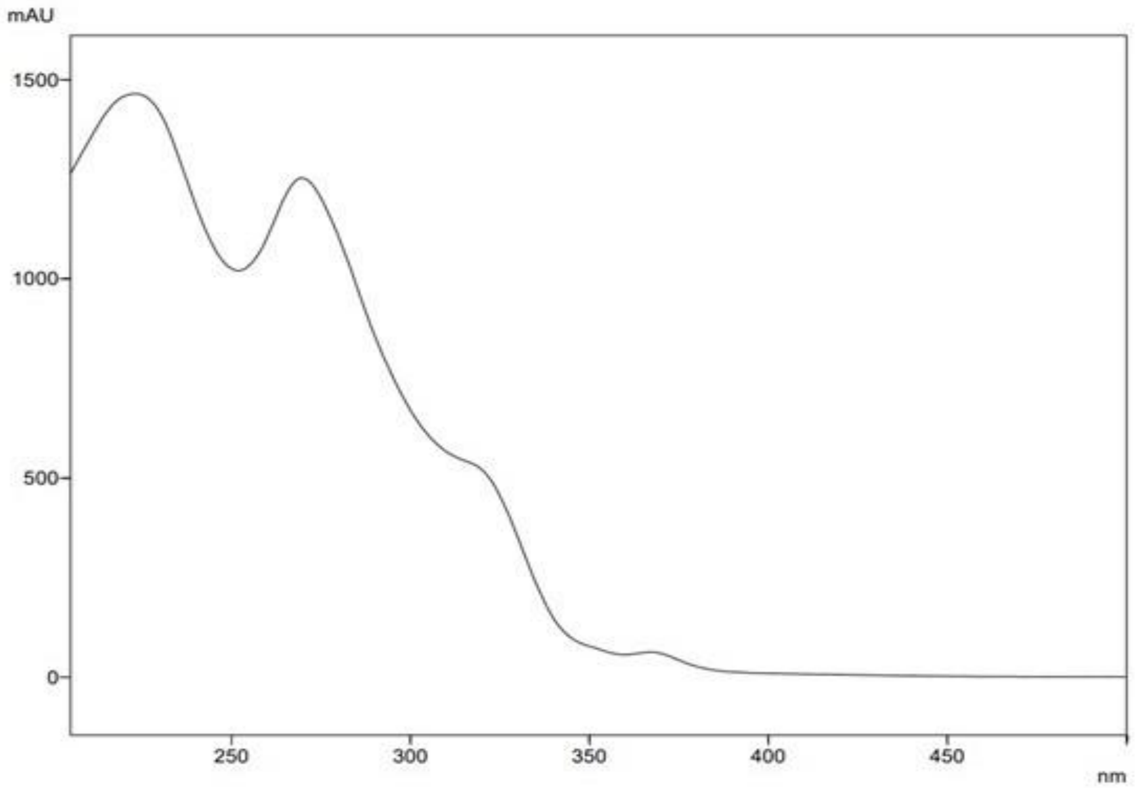

Figure S79. UV spectrum of compound **10** in MeOH.

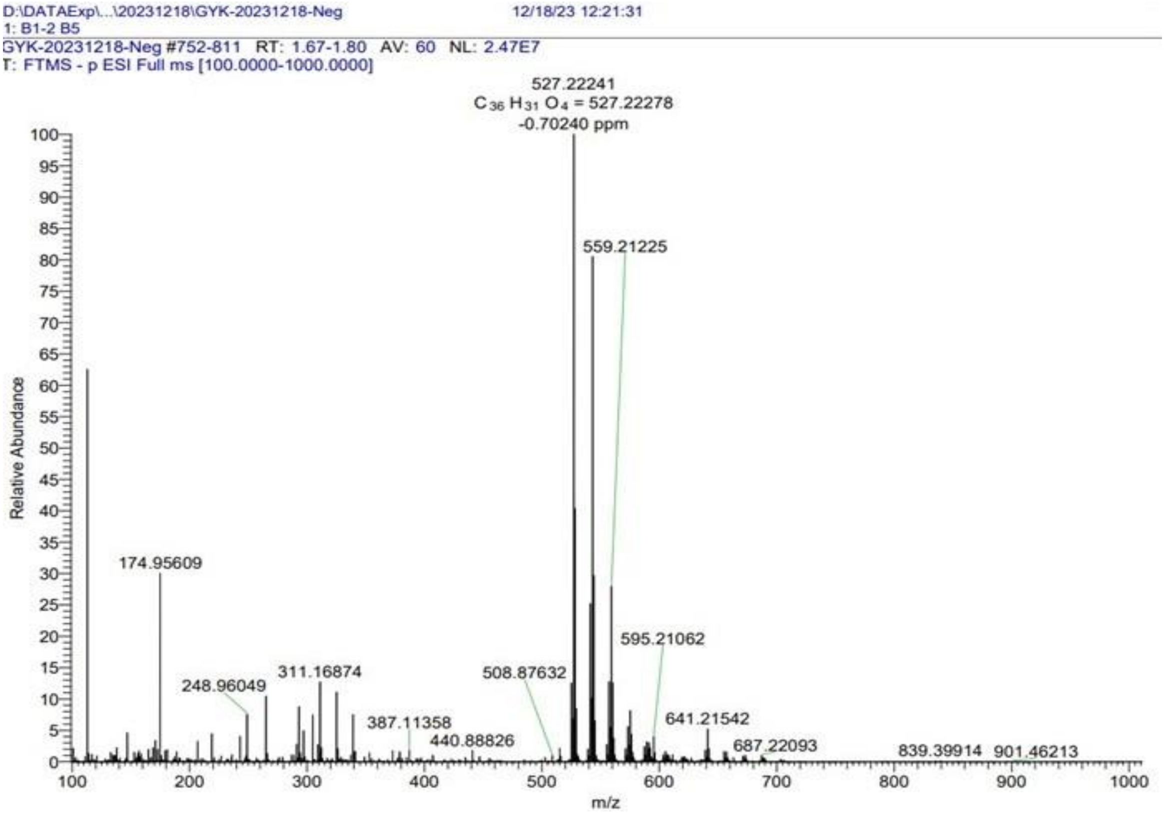

Figure S80. (–)-HRESIMS spectrum of compound **10**.
